# The Naucoridae (Heteroptera: Nepomorpha) of Madagascar, with revisions of *Temnocoris* and *Tsingala* (Laccocorinae)

**DOI:** 10.1371/journal.pone.0272965

**Published:** 2022-09-28

**Authors:** Robert W. Sites, Johannes Bergsten

**Affiliations:** 1 Division of Plant Sciences, Enns Entomology Museum, University of Missouri, Columbia, Missouri, United States of America; 2 Department of Zoology, Swedish Museum of Natural History, Stockholm, Sweden; Nanjing Agricultural University, CHINA

## Abstract

The island nation of Madagascar was surveyed extensively through a series of expeditions to determine the fauna of Naucoridae. Previously, 17 species in four genera had been reported from the country. All taxa previously recorded from Madagascar were re-collected, with the exception of three species, *Macrocoris flavicollis* Signoret, *Temnocoris starmuhlneri* Poisson, and *Tsingala nossibeanus* (Bergroth). *Macrocoris flavicollis* is removed from the list of species occurring in Madagascar. Within Laccocorini (Laccocorinae), a new genus, *Gonioathrix* **n.gen.** is described; *Temnocoris* and *Tsingala* are revised; three new species are described in *Temnocoris* (*T*. *leachi* **n.sp.**, *T*. *montandoni* **n.sp**., *T*. *poissoni* **n.sp.**) and four in *Tsingala* (*T*. *angulata* **n.sp.**, *T*. *latiforma* **n.sp.**, *T*. *spatulata* **n.sp.**, *T*. *trilobata* **n.sp.**). Lectotypes are designated for *Afronaucoris madagascariensis* (Montandon), *Tsingala humeralis* (Signoret), and *T*. *naucoroides* (Montandon). In Macrocorinae, a new species of *Macrocoris*, *M*. *namorona* **n.sp.**, from Ranomafana National Park is described. These taxonomic actions bring the total for the country to five genera and 25 species. Distributions, habitat associations, and a key to the species are presented.

## Introduction

Madagascar is well-known for its high biotic diversity and high endemism at both species and higher taxonomic levels, as well as the ongoing loss of taxa to environmental degradation [1,2]. It has been categorized as one of the eight most important biodiversity hotspots based on richness and endemism of vertebrates and plants and loss of natural vegetation [3]. It has also been considered a hotspot for freshwater biodiversity because of its unique fish and invertebrate communities [4,5]. Endemism levels in various freshwater groups range from 50 to 100% [6,7] but among stream insect taxa, more than 95% are endemic to Madagascar [8]. Notably, this endemism includes many species radiations from *in situ* speciation processes on the island [9]. For most groups, ancestors arrived from mainland Africa in the Cenozoic Era [10–12], with some notable more ancient exceptions [13]. While various charismatic terrestrial vertebrate lineages like lemurs, amphibians, day geckos and chameleons are among the best known examples, invertebrate inhabitants of freshwater ecosystems exhibit equal or greater endemism and diversification patterns in Madagascar [14]. However, habitat degradation from unsustainable agricultural practices such as the slash and burn approach (‘tavy’), leading to deforestation and drainage of wetlands, together with overfishing, and exotic species introductions have had catastrophic effects on the aquatic biota [6,14]. Over 40% of freshwater species across six representative groups are threatened with extinction according to a recent assessment [6]. The insect communities of streams draining forests in Madagascar were shown to be dramatically richer and more diverse in terms of broad taxonomic groupings and functional feeding groups than streams in nearby agricultural areas [15]. The most diverse biome in Madagascar is the eastern rainforest [16] where large portions of the diversity, including aquatic insects, remain to be charted and described [7,8]. However, with last decade’s rate of deforestation, undisturbed rainforests on Madagascar are predicted to be gone in fewer than 30 years [17]. Already, about half of Madagascar’s forests are less than 100 m from a forest edge [18]. Such increasing fragmentation of forested habitats have profound implications for streams, which are affected by upstream catchment conditions. This situation of high richness and endemism and large knowledge-gaps, combined with accelerating habitat loss and degradation [17–19], has created a heightened sense of urgency to study the aquatic insect fauna of Madagascar.

One of the more common groups of aquatic insects in Madagascar is the true bug family Naucoridae (Heteroptera: Nepomorpha), or saucer bugs. All species of Naucoridae and the related family Aphelocheiridae known from Madagascar are endemic to the island. Despite a series of taxonomic papers by Poisson in the middle 1900s, the fauna of Naucoridae is still not well-understood. The earliest work on the Naucoridae of Madagascar began with Signoret’s description of the common *Heleocoris humeralis* in 1860 [20], which is now in the genus *Tsingala* [21]. At the end of the 19th century, several species were described by E. Bergroth and A.L. Montandon. A half century passed until Poisson’s numerous species descriptions during the 1950s and 1960s. Since then, nothing further has been published concerning the saucer bugs of Madagascar other than their inclusion in a catalog and supplements of the world fauna [22–24]. Thus, after another half century of inactivity, presented here is an exhaustive work detailing the fauna of the Naucoridae of Madagascar following multiple collecting expeditions and the examination of available types. Included are revisions of *Temnocoris* and *Tsingala*, one new genus and nine new species descriptions, and lectotype designations. In addition, montage photographs, diagnoses, habitat associations, and a key to the species are provided.

## Materials and methods

Specimens of Naucoridae were collected during multiple expeditions from 2006 to 2019 by JB and collaborators to survey the Adephaga water beetle fauna in all regions of the country. Collecting was also conducted by RWS in 2014 in the eastern forests from Ranomafana National Park in the south to Marojejy National Park in the north. Photographs of collecting sites in 2014 identified as L-numbers are available in a Locality Image Database via a link from the internet site of the Enns Entomology Museum, University of Missouri. All measurements are given in millimeters (mm). Abdominal segment numbers are expressed as Roman numerals. Male abdominal segment VIII is represented by paired medial and lateral lobes; the medial lobes are here referred to as pseudoparameres. Images were obtained by use of a Leica MZ16 stereomicroscope coupled with the Leica Application Suite V4.10 Extend Depth of Focus module, followed by image preparation with Adobe Photoshop V21.2.1. In the Material examined sections, a question mark (?) indicates an illegible label, brackets [] indicate inferred details from existing data, and a slash (/) indicates separate labels of types and select other old specimens. Specimens are deposited in the museums and collections corresponding with the following acronyms. Specimens will also be shared with Parc Botanique et Zoologique de Tsimbazaza/Madagascar Biodiversity Centre, Antananarivo, Madagascar.

### Museum and collection acronyms

MCSN Museo Civico di Storia Naturale (Genoa)

MNHN Muséum National d’Histoire Naturelle (Paris)

NHMUK Natural History Museum (London)

NHMW Naturhistorisches Museum (Vienna)

SEMC Snow Entomological Museum Collection (Lawrence)

NHRS Swedish Museum of Natural History (Stockholm)

UMC University of Missouri (Columbia)

USNM United States National Museum of Natural History (Washington, D.C.)

### Nomenclatural acts

The electronic edition of this article conforms to the requirements of the amended International Code of Zoological Nomenclature, and hence the new names contained herein are available under that Code from the electronic edition of this article. This published work and the nomenclatural acts it contains have been registered in ZooBank, the online registration system for the ICZN. The ZooBank LSIDs (Life Science Identifiers) can be resolved and the associated information viewed through any standard web browser by appending the LSID to the prefix ""http://zoobank.org/"". The LSID for this publication is: urn:lsid:zoobank.org:pub:58B7E709-8498-487B-B292-2F81E3767F8A. The electronic edition of this work was published in a journal with an ISSN, and has been archived and is available from the following digital repositories: PubMed Central, LOCKSS.

### Ethics statement

Collections and exports were was conducted over the years based on permits issued by the Madagascar Ministère de l’Environment, d’Ecologie et des Forêts: No. 175 MINENVEF/SG/DGEF/DPB/SCBLF, No. 82/06/MINENV.EF/SG/DGEF/DPB/SCBLF/RECH, No. 250/06/MINENV.EF/SG/DGEF/DPB/SCBLF/RECH, No 266/09/MEF/SG/DGF/DCB.SAP/SLRSE, No 251/09/MEF/SG/DGF/DCB.SAP/SLRSE, No 250/11/MEF/SG/DGF/DCB.SAP/SCB, No 280/12/MEF/SG/DGF/DCB.SAP/SCB, No 288/13/MEF/SG/DGF/DCB.SAP/SCB, No 281/14/MEEF/SG/DGF/DCB.SAP/SCB, No 43/16/MEEMF/SG/DGF/DAPT/SCBT.Re, No 24/16/MEEMF/SG/DGF/DAPT/SCBT.Re, No 011/18/MEEF/SG/DGF/DSAP/SCB.Re, No 004/18/MEEF/SG/DGF/DSAP/SCB.Re, No.186/19/MEDD/SG/DGEF/DGRNE.

### Systematics

The saucer bug fauna of Madagascar consists of distinct clades within three subfamilies with all species endemic and restricted to Madagascar [21]. The probable origins of these clades are from mainland Africa. Unfortunately, the Bergroth and some Montandon type specimens have been lost over the years.

### Checklist

#### Laccocorinae

Laccocorini

*Gonioathrix temnocoroides* **new genus, new species**

*Temnocoris ambositrae ambositrae* Poisson, 1951

*Temnocoris ambositrae betiokyi* Poisson, 1951

*Temnocoris ambositrae magnus* Poisson, 1956

*Temnocoris andringitrae* Poisson, 1952

*Temnocoris dubius* Poisson, 1951

*Temnocoris hungerfordi* Poisson, 1952

*Temnocoris leachi* **new species**

*Temnocoris montandoni* **new species**

*Temnocoris perplexus* Poisson, 1951

*Temnocoris poissoni* **new species**

*Temnocoris scarletti* Poisson, 1941

*Temnocoris starmuhlneri* Poisson, 1962

*Temnocoris translucidus* Montandon, 1897

*Tsingala angulata* **new species**

*Tsingala humeralis* (Signoret, 1860)

*Tsingala latiforma* **new species**

*Tsingala naucoroides ambigua* (Poisson, 1962)

*Tsingala naucoroides naucoroides* (Montandon, 1897)

*Tsingala nossibeanus* (Bergroth, 1893)

*Tsingala spatulata* **new species**

*Tsingala trilobata* **new species**

### Naucorinae

Afronaucorini

*Afronaucoris madagascariensis* (Montandon, 1899)

*Afronaucoris parvulus* (Signoret, 1860)

### Macrocorinae

*Macrocoris distinctus* Bergroth, 1893

*Macrocoris namorona* **new species**

*Macrocoris rhantoides* Bergroth, 1893

*Macrocoris sikorae* Bergroth, 1893

### Annotated list of taxa

Species are presented below in alphabetical sequence by subfamily, genus, and specific epithet. New genus and new species descriptions follow the same sequence.

### Subfamily Laccocorinae Stål, 1876

#### Tribe Laccocorini Stål, 1876

**Fig 1:**
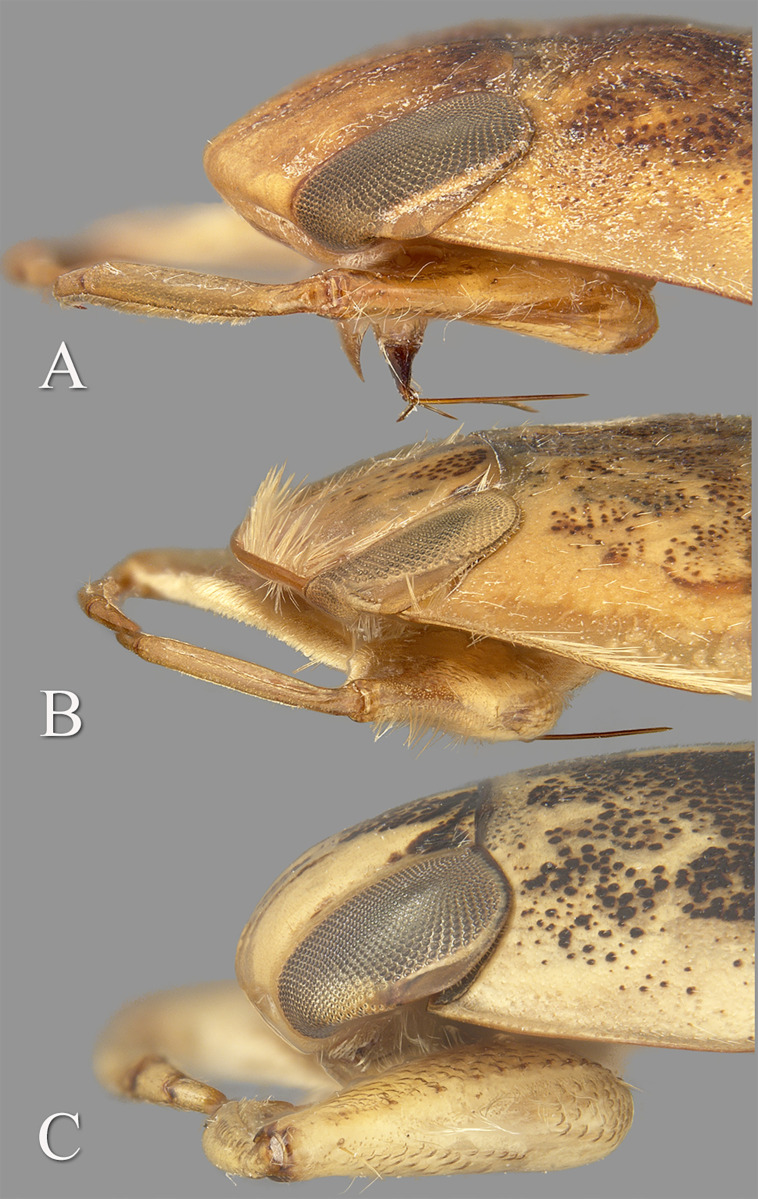
Three genera of Laccocorinae known from Madagascar. Dorsolateral view of head and pronotum of (A) *Gonioathrix* **n.gen**., (B) *Temnocoris*, (C) *Tsingala*.

In Madagascar, *Gonioathrix* **n.gen.**, *Temnocoris*, and *Tsingala* are the only genera of the subfamily Laccocorinae and together can be distinguished from the other two naucorid genera (*Afronaucoris* and *Macrocoris*) by the front of the head folded posteroventrally such that the labrum is set back from the anterior margin of the head (Fig 1). Other features associated with the legs, some of which are sexually dimorphic, also distinguish this subfamily [21].

### *Gonioathrix* NEW GENUS

urn:lsid:zoobank.org:act:53A3B0C6-6067-4A6E-BB87-212FD10580B1

(Figs 1A, 2 and 3)

Type species—*Gonioathrix temnocoroides* NEW SPECIES

#### Hindwing brachypterous male

**Fig 2:**
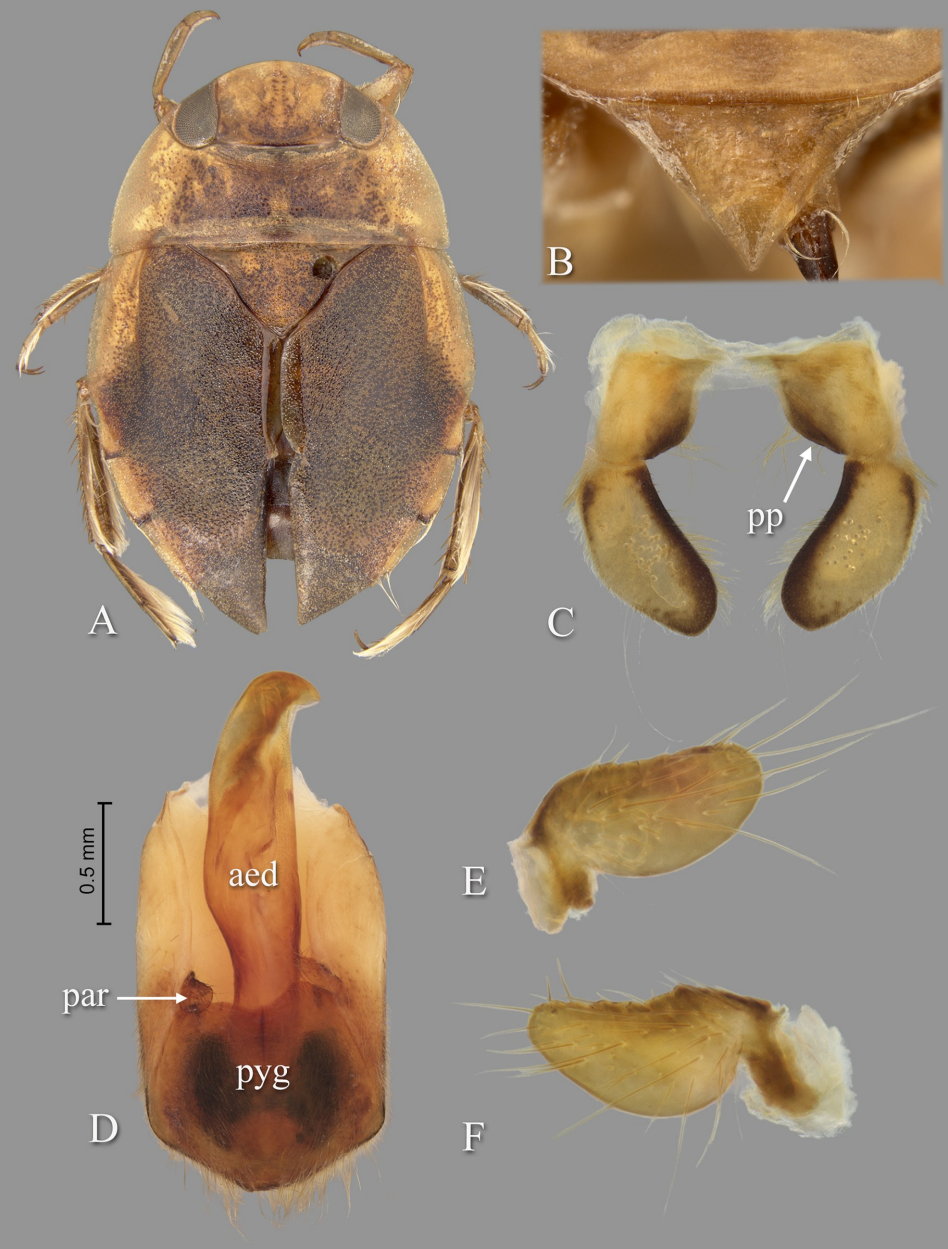
*Gonioathrix temnocoroides* n.gen, n.sp. (A) Submacropterous male holotype, (B) labrum (C) 8^th^ abdominal tergum, (D) male genital capsule with proctiger and tergum IX removed, (E) left paramere, (F) right paramere. aed = aedeagus, par = paramere, pp = pseudoparamere, pyg = pygophore. Size bar pertains only to Fig D.

Holotype: length 9.68; maximum width across embolia 6.56. Overall shape elongate-oval (Fig 2A).

*Head*. Length 1.88, maximum width 4.00, inner margin of eyes divergent anteriorly. Cuticle laterad of eye expanded and subtriangular. Anterior margin of head bluntly emarginated and without fringe of erect setae between eyes (Fig 1A). Row of setae emanating from row of pits or sulcus paralleling inner margin of eyes. Labrum triangular, acuminate distally (Fig 2B). Antenna with three visible segments, first segment probably concealed inside head capsule.

*Thorax*. Pronotum broad, 3.60× wider than long; coarse punctures in anterior 3/4, punctures brown in middle half, diminishing in apparency laterally, becoming concolorous with yellow ground color; transverse band across posterior 1/4 devoid of coarse punctures; lateral margin evenly convex and without fringe of long setae (although several elongate setae in posterior 1/3), posterolateral corners bluntly acute, posterior and anterior margins straight; ventrally, propleuron yellowish, mostly pruinose but with anterior and median lighter area, anterolateral lobe extending posteriorly approximately to middle of procoxa. Scutellum triangular, surface irregular, lateral margins distinctly sinuate, 1.90× wider than long. Hemelytra with lateral margin of embolium almost straight to shallowly convex for most of length, with degree of curvature greater near posterior end and humeral angle, with row of short spines but without fringe of long setae. Clavus with claval and intraclaval sutures present, but suppressed. Claval commissure longer than scutellum. Membrane reduced, narrowly overlapping at midline, broadly V-shaped, extending anteriorly to claval commissure and along costal margin almost to embolium. Hindwing extending almost to posterior margin of tergum III. Metaxyphus broad, apex obtusely pointed. All leg segments yellowish, darkening through tibiae, tarsi, and pretarsi. Profemur elongate, wide, anterior margin with dorsal and ventral rows of golden hairs sandwiching one row of shorter, wide, brown setae. Distal 3/4 of protibia and two-segmented protarsus with dense ventral pad of setae. Propretarsus with short, stout, paired, movable claws. Mesofemur with anteroventral and mid-ventral rows of elongate setae; metafemur with anteroventral row and mid-ventral row of short, spinose setae, and posteroventral surface set with short spinules. Mesotrochanter and mesofemur with profuse brush of light colored setae on posterior margin. Metatrochanter with brush of setae less prevalent and with posterior margin evenly convex. Elongate, narrow, profuse pad of setae on mesotibia and mesotarsomeres 2 and 3. Long golden swimming hairs sparse on mesotibia and tarsus, profuse on metatibia and tarsus. Middle and hind legs with tarsomere 1 extending beneath tarsomere 2. Meso- and metathoracic pretarsal claws long, straight, with slight curvature.

*Abdomen*. Dorsally, terga II–VII brownish-yellow and lined with black on posterior margins; lateral margins of III–VII with regular row of short, stout, yellow spines and tuft of elongate setae at posterolateral corners. Ventrally with mid-ventral longitudinal band of elongate setae. Sternum II entire, sterna III–VII divided into medio- and laterosternites.

#### Diagnosis

*Gonioathrix* shares features of both *Temnocoris* and *Tsingala*. Its facies is similar to that of *Temnocoris*, but without the fringe of setae along the anterior margin between the eyes and laterally on the pronotum. The front of the head is bluntly angled (Fig 1A), rather than sharply as in *Temnocoris* (Fig 1B), but not bullnosed as in *Tsingala* (Fig 1C).

#### Discussion

This new genus has attributes consistent with the subfamily Laccocorinae, including the well-developed pad of hairs on the pro- and mesotibiae and -tarsi in males (present but reduced in females), and paired, articulated propretarsal claws. Further, we assign *Gonioathrix* to the tribe Laccocorini, which in Madagascar previously included only the endemic genera *Temnocoris* and *Tsingala*. Because this new genus shares features of both *Temnocoris* and *Tsingala* (Table 1), its exact phylogenetic position is unclear. However, we consider it to be closer to *Temnocoris* primarily because of male genitalic similarity with apical modifications of the aedeagus.

**Table 1:** Character comparison of *Gonioathrix* with *Temnocoris* and *Tsingala*, the three genera of Laccocorinae known from Madagascar.

Character	*Gonioathrix*	*Temnocoris*	*Tsingala*
compound eyes	slightly divergent	strongly divergent	parallel or slightly convergent
labrum shape	acuminate	rounded	rounded
front of head	bluntly margined	sharply margined	bullnosed
front of head fringe	none	profuse	none
anterior production of head	strong	strong	weak
anterior production of pronotum corners	strong	strong	weak
meso/metaleg segment proportions^a^	1.43	1.17	1.42
metafemur anteroventral seta row	short	mixed sizes	short
hindwing condition of submacropterous forewing form	brachypterous	micropterous	micropterous
sterna III–V	divided	entire	divided
sternum V symmetry	symmetrical	asymmetrical	asymmetrical
pseudoparameres	reduced	well-developed	well-developed
habitat	lentic	lotic	mixed
aedeagus	distinctly modified	distinctly modified	tapered

^a^Proportional lengths of the mesofemur to mesotibia compared to those of the hindleg.

#### Habitat

**Fig 3:**
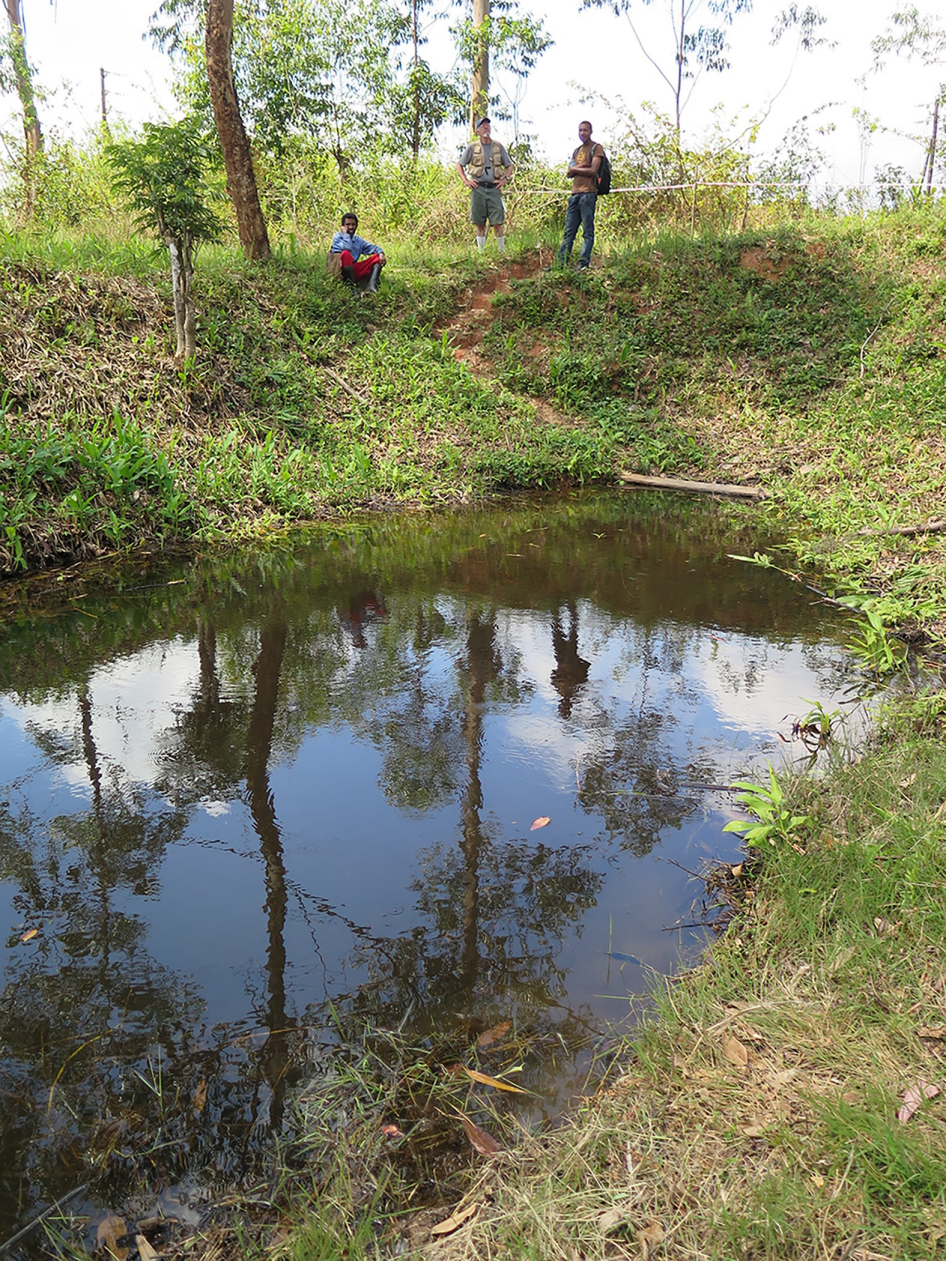
Gonioathrix temnocoroides n.gen, n.sp. Type locality at Toamasina Province, Centmetre, near northwest border of Analamazaotra National Park, Andasibe Village.

This species was collected at only one locality–a pond with steep banks and margins with submerged grasses, other vegetation, and dead branches (Fig 3) near the northwest border of Analamazaotra National Park, Andasibe Village. Other saucer bugs collected with it were *Afronaucoris parvulus* and *Tsingala humeralis*.

#### Etymology

The genus name is a combination of *Gonio* (= angle or corner, Greek) and *athrix* (= hairless, Greek), which is in reference to the angled, hairless anterior margin of the head as opposed to the bullnosed anterior margin of *Tsingala* and the fringe of hair in *Temnocoris*.

#### Material examined

See Type material examined.

### *Gonioathrix temnocoroides* NEW SPECIES

urn:lsid:zoobank.org:act:16FBF1BB-AE89-4C32-85B0-ABAC7FB54145

(Figs 1A, 2 and 3)

#### Hindwing brachypterous male

See generic description; only additional details given here. Dorsally, overall coloration dark-yellow with profuse black speckling on hemelytra (Fig 2A). Coarse punctation on pronotum. Ventrally, head, thorax, and legs dark yellow; abdomen light-brown. Synthlipsis at posterior margin 2.00. Vertex with coarse, brown punctation on each side of midline, continuing as paired lines of punctures anteriorly. Labrum 1.81× wider than long. Antenna length 0.60, proportions 11, 12, 7. Pronotum dark-yellow, length at midline 1.60; maximum width at posterolateral corners 5.76. Scutellum yellow with black paramedian punctures, width 3.20, length 1.68. Hemelytra orangish-brown and with profuse black speckling throughout corium, clavus, membrane, and posterior third of embolium. Membrane with fine, white punctures. Embolium length 3.20, greatest width 1.04. Claval commissure length 2.00. Leg measurements as follows: foreleg, femur 2.48, tibia 1.62, tarsomere 1 0.36, tarsomere 2 0.32; middle leg, femur 2.56, tibia 1.70, tarsomeres 1–3 0.12, 0.34, 0.44, pretarsal claws 0.44; hind leg, femur 3.04, tibia 2.88, tarsomeres 1–3 0.20, 1.14, 1.86, pretarsal claws 0.54. Abdominal terga II–VII brownish-yellow and lined with black on posterior margins; lateral margins of III–VII with regular row of short, stout, yellow spines and tuft of elongate setae at posterolateral corners. Tergum VIII with medial lobes (pseudoparameres) reduced (Fig 2C). Posterior margins of sterna III–VII symmetrical. Posterolateral corners of II–III right angled, IV–V acute. Aedeagus narrow basally, abruptly widening on left side near mid-length, right side straight until apex, left side gradually curving to right, apex hooklike, acute, and directed to right (Fig 2D). Pygophore with anterior margin broadly and asymmetrically concave between parameres, brush of long setae most prominent posteriorly (Fig 2D). Parameres asymmetrical, directed mesad, and slightly arcuate to match aedeagus curvature; left paramere with anterior margin nearly straight, posterior margin evenly convex, and apex broadly rounded; right paramere with anterior margin shallowly concave, posterior margin convex in basal half and straight in distal half, apex rounded; both parameres with exceptionally elongate setae on dorsal surfaces, length of longest setae exceeding width of paramere (Fig 2E and 2F).

#### Female and macropterous form

Unknown.

#### Diagnosis

See genus diagnosis. This is the only known species of the genus.

#### Etymology

The specific epithet *temnocoroides* is in reference to the similar dorsal habitus appearance to that of species of *Temnocoris*.

#### Type material examined

HOLOTYPE hindwing brachypterous ♂: Madagascar: **Toamasina:** Centmetre, 18°55.626’S, 48°25.219’E, elev. 935 m, 15-XI-2014, R.W. Sites, pond with grasses, L-1869 (UMC).

### Genus *Temnocoris* Montandon, 1897

*Temnocoris* Montandon 1897b: Verh. Zool. Bot. Ges. Wien. 47: 437–438. Type species: *Temnocoris translucidus* (Montandon, 1897), by monotypy.

This Madagascar endemic genus was erected by Montandon [25] to contain the species *T*. *translucidus* Montandon; all other currently recognized species and subspecies were described by Poisson in the middle of the 20th century. The genus currently contains eight species, two of which are further apportioned into subspecies, one of which is synonymized here. *Temnocoris* can easily be distinguished from the other two genera of Laccocorinae in Madagascar by the anterior margin of the head, which is sharply margined and with a fringe of hairs in *Temnocoris* (Fig 1B), bullnosed and hairless in *Tsingala* (Fig 1C), and bluntly angled and hairless in *Gonioathrix* (Fig 1A).

*Temnocoris* is a relatively straight-forward genus taxonomically because readily apparent characters are available separately for males and females of most species. The phallosoma and parameres are the most reliable distinguishing features in the males, with additional characters in the shape of the pseudoparameres (medial lobes of tergum VIII). The shape of the female subgenital plate is diagnostic for many of the species, although Poisson did not describe this structure for his new species. Unfortunately, females of some species were not available for us to examine; thus, not all subgenital plates are given here. Few other characters presented by Poisson are reliably consistent within species; thus, these also are not presented here. Body size and dorsal coloration are intraspecifically variable and generally not taxonomically reliable. Three new species are reported here. The subgenital plates of some species are similar and of other species are unknown. Thus, determinations of females for some species might require association with identifiable males. This genus is not commonly collected, and when it is found, usually only a few specimens are taken. It is known only from lotic systems; we have collected it mostly on sandy substrates.

**Fig 4:**
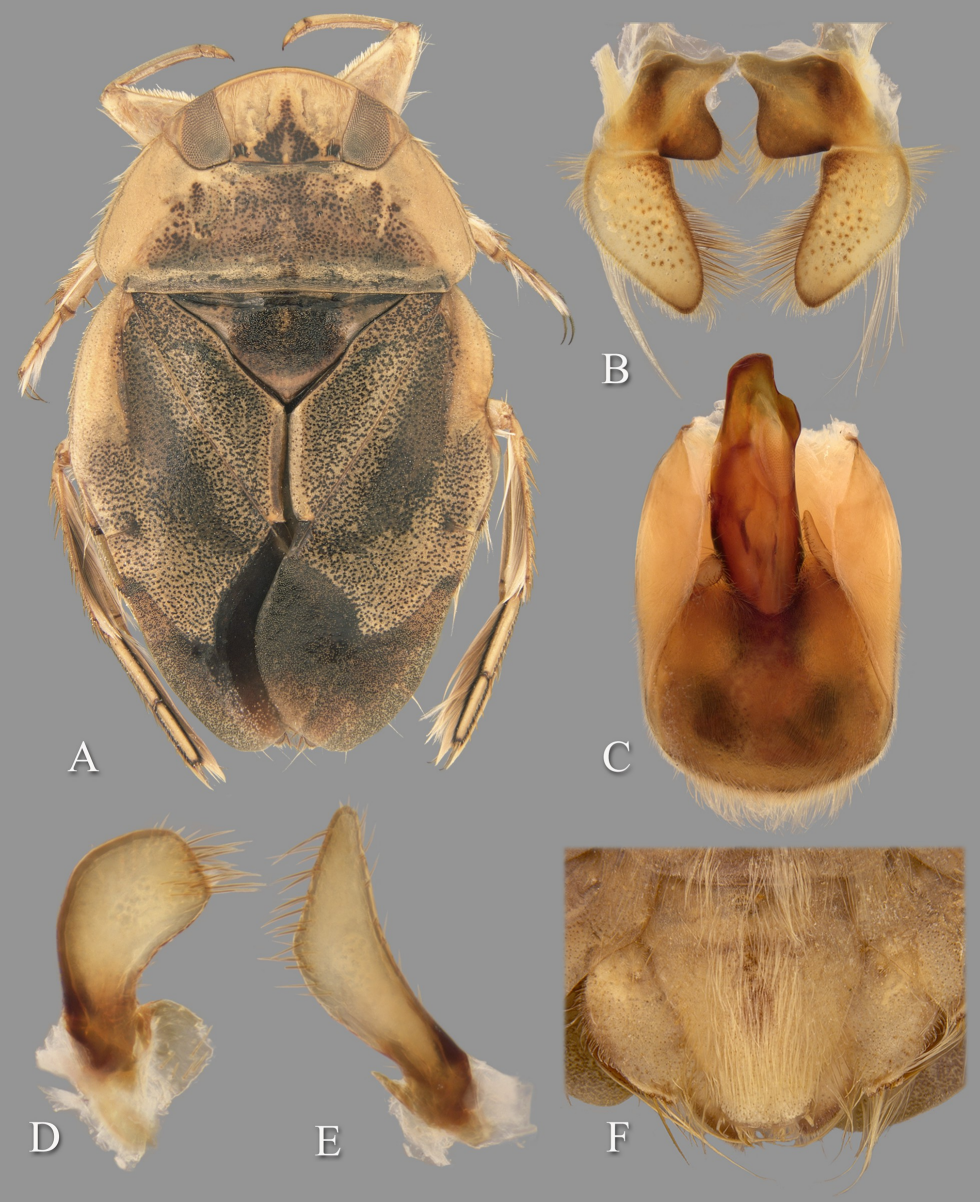
Temnocoris ambositrae. (A) Macropterous male, (B) male 8^th^ abdominal tergum, (C) male genital capsule with proctiger and tergum IX removed, (D) left paramere, (E) right paramere, (F) female subgenital plate.

**Fig 5:**
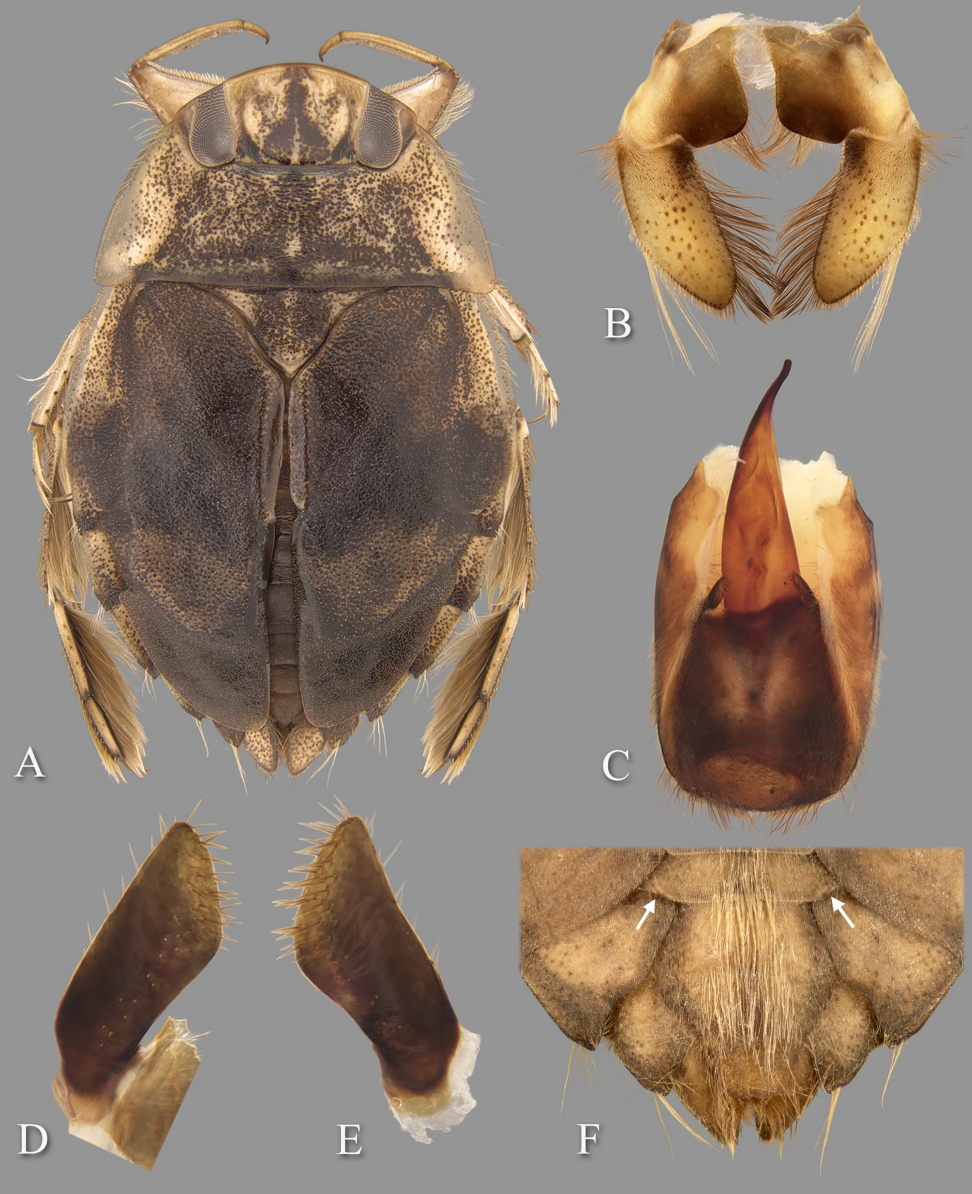
Temnocoris andringitrae. (A) Submacropterous female, (B) male 8th abdominal tergum, (C) male genital capsule with proctiger and tergum IX removed, (D) left paramere, (E) right paramere, (F) female terminal abdominal sterna with subgenital plate. White arrows indicate elevated posterolateral angles of mediosternite VI forming pockets.

Many of Poisson’s descriptions referenced "subbrachypterous" specimens. This alary condition consists of forewings with non-overlapping membranes, in which each membrane extends from the wing apex forward along the lateral margin almost to the equivalent level of the tip of the hemelytral commissure (e.g., Fig 5A), claval sutures that are suppressed and only faintly visible, and hindwings barely achieve abdominal tergum II. We refer to this condition with submacropterous forewings and micropterous hindwings as submacropterous, in reference to the visible forewings. In contrast, some conspecific specimens are macropterous with overlapping membranes of the forewings, typical of adult Heteroptera, and fully developed hindwings extending approximately to abdominal tergum VII (e.g., Fig 4A).

### *Temnocoris ambositrae* Poisson, 1951

(Fig 4)

*Temnocoris ambositrae* Poisson 1951: Mem. Inst. Sci. Madag. A5: 104–106 (original description).

*Temnocoris ambositrae*: La Rivers 1971, Biol. Soc. Nev. Mem. 2: 79 (catalog incl. all subspecies).

*Temnocoris a*. *ambositrae*: Poisson 1963, Bull. Inst. Fr. Afr. Noire 25: 1181 (distribution).

*Temnocoris a*. *ambositrae*: La Rivers 1974, Biol. Soc. Nev. Occas. Pap. 38: 13 (catalog).

*Temnocoris ambositrae betiokyi* Poisson 1951: Mem. Inst. Sci. Madag. A5: 105–106 (original description).

*Temnocoris ambositrae betiokyi*: Poisson 1952, Mem. Inst. Sci. Madag. E1: 59 (figures).

*Temnocoris ambositrae magnus* Poisson 1956: Mem. Inst. Sci. Madag. E7: 257–259 (original description).

#### Discussion

Two subspecies were described by Poisson: *T*. *ambositrae betiokyi* Poisson [26] and *T*. *ambositrae magnus* Poisson [27]; thus including the nominate subspecies, three subspecies are known. *Temnocoris a*. *betiokyi* was described based on only four characters of one male specimen: smaller size, pronotum 3× wider than long, scutellum twice as long as wide, and right lobe of segment VIII as shown in his Fig 27B. The *T*. *a*. *magnus* description was based on three characters of both sexes from Ikopa River at Marovoay-Majunga: larger than the nominate subspecies, two small sublinear markings on the pronotum and a rounded spot on the anterior margin of the pronotum that are more reddish than other markings, and the ventrolateral subapical lamellar expansion of the aedeagus is more pronounced. We do not have enough comparative material from throughout the range of the species from which to assess the validity of these subspecies designations; however, we suspect that this is simply intraspecific variation that does not warrant recognition as subspecies.

#### Diagnosis

The aedeagus combined with the right paramere is diagnostic for males; the apex of the aedeagus is rounded on the left side and hooked on the right, and the subapical flap on the right side is reflexed dorsad (Fig 4C). Although one other species (*T*. *montandoni* **n.sp.**) has a similar aedeagus shape, *T*. *ambositrae* can be distinguished from it by the right paramere, which is elongate and angled near the middle (Fig 4E). The subgenital plate shape can distinguish the females, although other species have a similar shape and the conditions for females of some species are unknown. More specifically, the lateral margins are straight to slightly concave and convergent to a roundedly truncate apex (Fig 4F).

#### Published records

*Temnocoris a*. *ambositrae*: Ambositra, Centre-Sud [26]; Tananarive: sud de col des Tapia; Fianarantsoa: Zomandao, west of Ihosy [28]. *Temnocoris a*. *betiokyi*: Betioky [29]. *Temnocoris a*. *magnus*: Ikopa River at Marovoay-Majunga [27].

#### Type material examined

HOLOTYPE ♂: *T*. *a*. *ambositrae*: Unknown province: centre sud, 11 mm, type, Poisson to Drake Coll 1979, Temnocoris ambositrae n.sp. (USNM). HOLOTYPE ♂: *T*. *ambositrae magnus*: Marovoay Western Madagascar Province: Majunga River, Ikopa, 1927 & 28 (SEMC); Syntypes: same data, Poisson to Drake Coll 1979, ♂, R. Poisson det. 1955, Temnocoris ambositrae f. magnus nov. (USNM); same locality, but with / ♂ / R. Poisson det., Temnocoris ambositrae Poiss. 1951, f. magnus nov. (1♂ SEMC); same locality, but also Temnocoris det. H.B. Hungerford / ♂ / R. Poisson det., Temnocoris ambositrae Poiss. 1951, f. magnus nov. (1♂ SEMC).

#### Material examined

**Antsiranana:** ANJI: Amilobe, Antsabe, Antsaba River, N-13.67840, E48.75670, 400 m, leg. Monaghan et al., P25MD14 (2♂, 1 nymph NHRS). **Fianarantsoa**: Namorona River near Vohiparara, 1200 m, 22-III-1990, W.E. Steiner, C. Kremen, V. Razafimahatra collectors (7♂, 3♀ USNM); N of Ambohimanjaka, Antanavierna River, 20°10.260’S, 47°5.437’E, elev. 1355 m, 5-XI-2014, R.W. Sites, shallow, sandy w/ veg. margins, L-1846 (2♂, 2♀, 16 nymphs UMC); Isalo Menamaty R., N-22°29.359, E45°23.505,715 m, sandy/stony bottom with marginal vegetation, leg. Bergsten et al., BMNH(E)742417 (1♂ NHRS); N of Ambohimanjaka, Antanavierna River, 20°10.260’S, 47°5.437’E, elev. 1355 m, 1-XI-2014, R.W. Sites, sandy bottom, irrigation channel, L-1828 (1♂, 3♀, 6 nymphs UMC); Isalo, Canyon de Rats, S-22.47971, E 45.37697, 742 m, 14-XI-2012, leg. Bergsten Bukontaite, Ranarilalatiana, Randriamihaja, MAD12-07 (1♂ NHRS). **Mahajanga**: Melaky, btw. Morafenobe-Ambohijanahary, S18.20675, E045.31783, 711 m, 19-XII-2009, leg. J. Bergsten, N. Jönsson, T. Ranarilalatiana, HJ. Randriamihaja, MAD09-75 (2♂, 1 nymph NHRS); 16 km SE of Andriba, 8 Nov. 1986, JT & DA Polhemus, CL2271 (1♂, 1♀ UMC); Melaky/Menabe, Ambohijanahary NP, S 8.26849, E45.46346, 906 m, 19-XII-2009, leg. J. Bergsten, N. Jönsson, T. Ranarilalatiana, HJ. Randriamihaja, MAD09-76 (2♂, 1♀ NHRS); Marovoay Western Madagascar Prov.: Majunga River, Ikopa, 1927 & 28 (4♂ SEMC). **Toamasina**: Alaotra, Mangoro, RN2, 2 km E Anevoko, "La Cascade" hotel, 13-XI-2011, stony river, 18.94427, E048.47992, 880 m, MAD11-43 (1♂, 1♀ UMC, 1♀ - DNA extracted UMC). **Toliara**: 85 km E of Tulear, 5-XII-1986, DA Polhemus, CL2298 (1♂ UMC); 3.5 km N Betroka, 758 m, 23°14’00"S, 46°05’07"E, 20-XI-1994, pool in wash, M.A. Ivie & D.A. Pollock (2♂ UMC). **Unknown province**: N.W. Madagascar, 30-X-[19]07, J.J.Lloyd, 1908–193 (1♂ BMNH).

### *Temnocoris andringitrae* Poisson, 1952

(Fig 5)

*Temnocoris andringitrae* Poisson 1952: Mem. Inst. Sci. Madag. E1: 58, 60–61 (original description).

*Temnocoris andringitrae*: La Rivers 1971, Biol. Soc. Nev. Mem. 2: 79 (catalog).

#### Discussion

This species was based on one male and two female "subbrachypterous" specimens. Nothing further has been published concerning this species other than its inclusion in the La Rivers catalog [22]. This species is little known and poorly understood. Poisson [29] characterized the species based on color, the vertex 1.12× wider at base of the eyes than length at midline, the pronotum with posterolateral corners slightly curved posteriorly and 2.8–3.2 times wider at the base than long at the middle, and the scutellum 2.20× wider at base than long. He gave the lengths of two specimens as 11.5 and 11.8 mm, but did not specify the sexes. Our two adult males measure 11.8 and 12.0 mm.

#### Diagnosis

This is among the largest species of *Temnocoris*. The aedeagus is diagnostic with the slender apex with recurved tip (Fig 5C). The pygophore is asymmetrical with the right side extending further forward to approximately half the distance of the right paramere (Fig 5C). The pseudoparameres have truncate posterior margins and broadly rounded posteromedial corners (Fig 5B), and the parameres are similarly shaped (Fig 5D and 5E). The female subgenital plate lateral margins are convergent to a roundedly truncate apex, and the posterolateral angles of mediosternite VI are elevated and forming pockets above laterosternite VII (Fig 5F).

#### Published records

Andringitra [29].

#### Type material examined

HOLOTYPE ♂: [**Fianarantsoa]**: Andringitra, m. 49., ♂, 11.5–11.8 mm, type, Poisson to Drake Coll 1979, Temnocoris andringitrae n.sp. (USNM).

#### Material examined

**Fianarantsoa**: N of Ambohimanjaka, 20°14.019’S, 47°5.611’E, elev. 1461 m, 5-XI-2014, R.W. Sites, cascade w/ pools, marginal grasses, L-1845 (1♂, 15 nymphs UMC); Haute Matsiatra, PN Andringitra, stream Riampotsy, S22°10’56.9", E046°53’53.9", 2087 m, 2- XII-2013, pools on rocks at side of river, leg. T. Ranarilalatiana & J.H. Randriamihaja, MAD13-32 (1♂ NHRS); Sendrisoa, Ambilavao, N-22°0.585, E46°57.024, 1165 m, 7-V-2006, standing water with vegetation, leg. Bergsten et al, BMNH(E)742186, DNA extracted (1♀ UMC).

### *Temnocoris dubius* Poisson, 1951

(Fig 6)

*Temnocoris dubius* Poisson 1951: Mem. Inst. Sci. Madag. A5: 108–109 (original description).

*Temnocoris dubius*: Poisson 1956, Mem. Inst. Sci. Madag. E7: 257 (new record and figures).

*Temnocoris dubius*: La Rivers 1971, Biol. Soc. Nev. Mem. 2: 79 (catalog).

**Fig 6:**
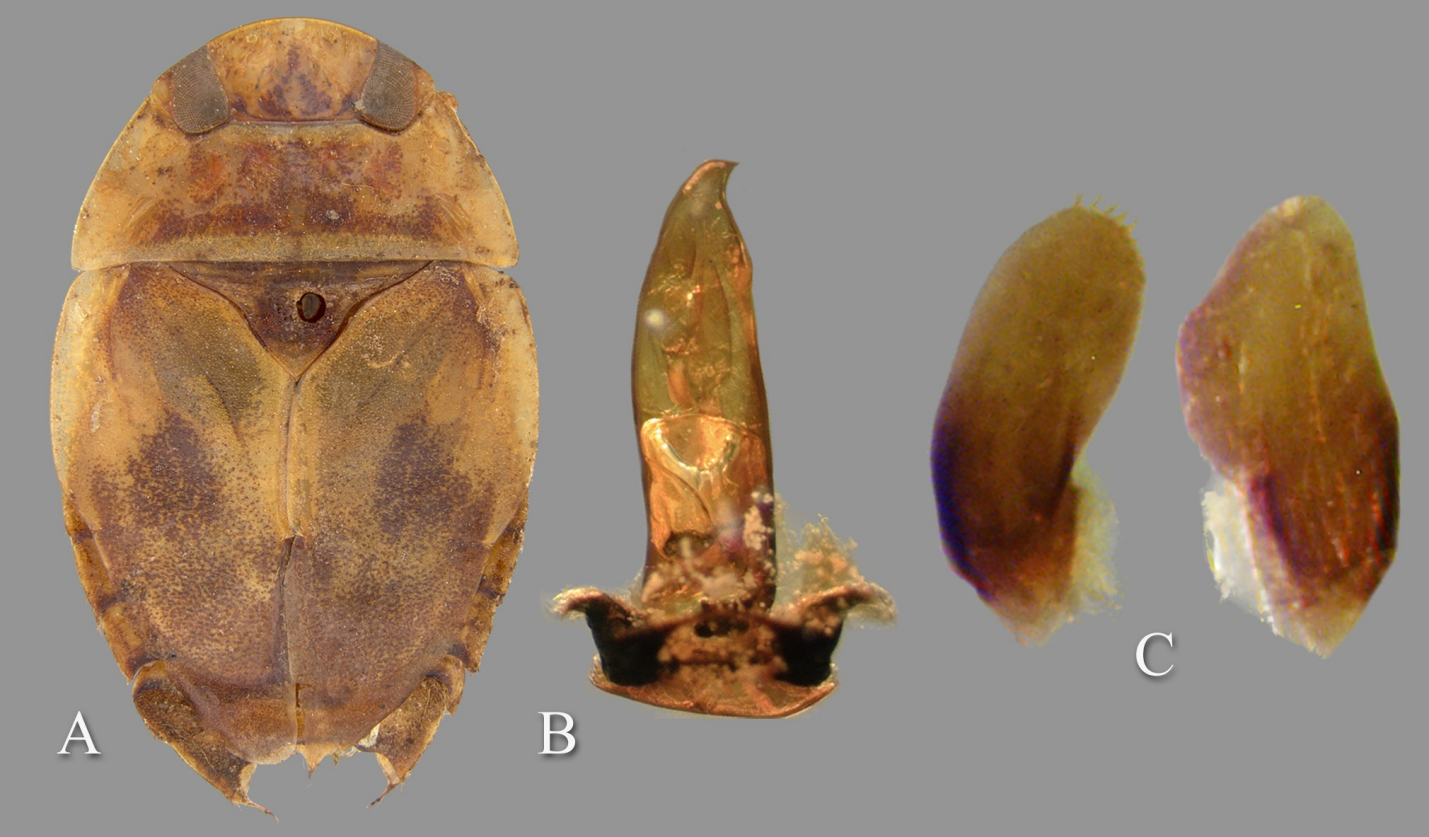
Temnocoris dubius. Specimen from Majunga River, Ikopa, determined by Poisson. A) Submacropterous male (SEMC), B) aedeagus (USNM), C) left and right parameres (USNM). B and C were photographed from slides in the Poisson slide collection.

#### Discussion

Poisson [26] described the species based on a single male specimen that was mutilated (“trés mutilé”) from centre-sud Madagascar, and stated that the pigmentation is similar to that of *T*. *scarletti* and *T*. *translucidus*. He characterized the specimen mostly based on color (Fig 6A) and shape of the aedeagus. He referred to the specimen as brachypterous, subbrachelytrous, and micropterous and that it is the largest of the genus at 13 mm length. A subsequent record gave the length of a second specimen as 12.5 mm [27]. A species described later, *T*. *starmuhlneri* Poisson, was considered to have a similar appearance to that of *T*. *dubius* [30].

#### Diagnosis

The apex of the aedeagus (Fig 6B) is unique among known species of *Temnocoris*; however, Poisson’s Fig 35c was reversed and the hook should be directed to the right. Poisson is known to have presented other figures in reverse (e.g., *T*. *perplexus* aedeagus). The shapes of the parameres (Fig 6C) and pseudoparameres (Poisson [26], Fig 34A) are also diagnostic. Females are not known.

#### Published records

Centre-sud [26].

#### Type material examined

HOLOTYPE ♂: [no locality label], type, Temnocoris dubius n.sp., Poisson to Drake Coll 1979 (USNM).

#### Material examined

Marovoay Western Madagascar Prov. Majunga River, Ikopa, 1927 & 28 / R. Poisson det. 1955, Temnocoris dubius Poiss./R. Poisson det 1955, Type no II. / 5310 Mixed lot!, det. H.B. Hungerford / SEMC1127407, KUNHM-ENT (1♂ SEMC); same collection data / R. Poisson det. 1955, Temnocoris dubius Poiss. 1951 / Type 2 / SEMC1127408, KUNHM-ENT (1♂ SEMC).

### *Temnocoris hungerfordi* Poisson, 1952

(Fig 7)

*Temnocoris hungerfordi* Poisson 1952: Mem. Inst. Sci. Madag. E1: 60–62 (original description).

*Temnocoris hungerfordi*: La Rivers 1971, Biol. Soc. Nev. Mem. 2: 79 (catalog).

**Fig 7:**
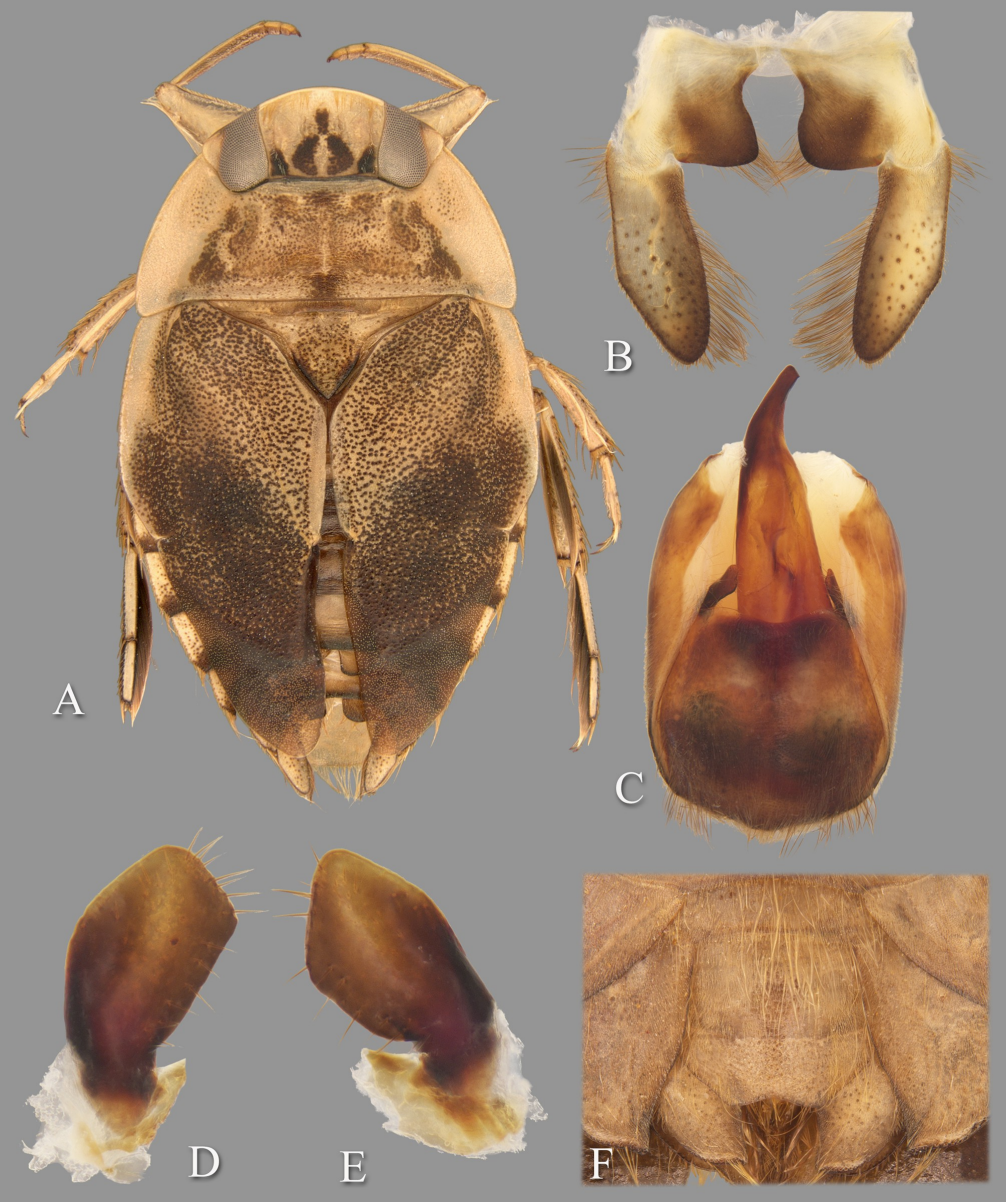
Temnocoris hungerfordi. (A) Submacropterous male, (B) male 8^th^ abdominal tergum, (C) male genital capsule with proctiger and tergum IX removed, (D) left paramere, (E) right paramere, (F) female terminal abdominal sterna with subgenital plate.

### Discussion

This is one of the more common species of *Temnocoris*, although after the original description, nothing has been published other than its inclusion in the La Rivers catalog [22].

### Diagnosis

The male can be easily recognized by the aedeagus, with the short, narrow distal end that angles to the right with a truncate apex (Fig 7C). The lateral lobes of tergum VIII are also diagnostic with the straight lateral margin that angles 30 degrees at midlength to continue straight to the posterior end (Fig 7B). The parameres are short and similar to each other in size, but differ in the lengths of the distal margins and degree of angle of the anteriormost corners (Fig 7D and 7E). The female subgenital plate is unique among the species for which it is known; it is about as wide as long and the lateral margins angle convergently at ~45 degrees to the wide, distinctly shallowly concave distal margin (Fig 7F).

### Published records

Tananarive [29].

### Type material examined

HOLOTYPE ♂: Madagascar, Tananarive, Bought, 1937, C. Lamberton / R. Poisson det., 1951: Temnocoris hungerfordi n.sp. / B Det. H.B. Hungerford / ♂ / HOLOTYPE Temnocoris hungerfordi Poisson (SEMC). PARATYPES: Tananarive, Bought, 1937, C. Lamberton / R. Poisson det., 1951: Temnocoris hungerfordi n.sp. / ALLOTYPE Temnocoris hungerfordi Poisson (SEMC). same data label as holotype / cotype / Poisson to Drake Coll 1979 (USNM); Tananarive, Bought, 1937, C. Lamberton / Temnocoris hungerfordi n.sp. / Paratype, Temnocoris hungerfordi, R. Poisson (1♂, 1♀ SEMC); same data, but parts of seven specimens on four cards on one pin / Paratype, Temnocoris hungerfordi, Poisson (unknown sexes, SEMC).

### Material examined

**Antananarivo**: Analamanga, Ambohitantely special reserve, 18.1808S, 47.2901E, 1340 m, 22-XI-2014, forest stream with waterfalls and over bedrock, leg. J. Bergsten, R. Bukontaite, J.H. Randriamihaja, T. Ranarilalatiana, S. Holmgren, MAD14-76 (1♂ NHRS). **Antsiranana**: Anjanaribe Sud NP, River Marolakan, 14.7623S, 14.7623S, 920 m, 15-XI-2014, large rocky river, leg. J. Bergsten, R. Bukontaite, J.H. Randriamihaja, T. Ranarilalatiana, S. Holmgren, MAD14-64 (1♂ NHRS); ANJI: Amilobe, Antsabe, Antsaba River, N-13.67840, E48.75670, 400 m, leg. Monaghan et al., P25MD14, DNA extracted (1♂ UMC); Vohémar, Madagascar / Temnocoris translucidus, 1946, L. Hoberlandt det. (1♀, 3 nymphs SEMC); Madagascar, Vohémar / Temnocoris translucidus, 1946, L. Hoberlandt det. (1♂, 1♀ SEMC); same locality, 5312 det by Hoberlandt, det. H.B. Hungerford / ♂ / R. Poisson det. Temnocoris hungerfordi Poiss. 1952 (1♂ SEMC). **Fianarantsoa**: Ranomafana national park, Fompononona, S21°17’S, 47°26’E, 18-V-1996, coll. J.P. Benstead (1♂, 1♀ UMC); 7 km W Ranomafana, 1100 m, 8–21 October 1988, W.E. Steiner, from stream with mossy rocks and sandy bottom, montane rainforest (4♂, 2♀ USNM); 7 km W Ranomafana, 900 m, 20–24-III-1990, W.E. Steiner (1♂, 1-5th instar USNM); 7 km SW Ranomafana, 1200 m, 23-X-1988, W.E. Steiner, C. Kremen, R. Van Epps, collrs., from stream with mossy rocks and sandy bottom, montane rainforest (1♂, 1♀ USNM); 8 km SW Ranomafana, 1040 m, Valahoaka camp, 21°19’S, 47°24’E, 8-IX-1993, netted from small sandy stream in montane rainforest, W.E. Steiner & J.E. Cadle, collrs. (1♂, 3♀ USNM). **Toamasina**: Alaotra-Mangoro, Zahamena NP Antanandava Sect., Manambato River by Camp Cascade, 17.5450S, 48.7237E, 1290 m, 10-III-2018, leg. J. Bergsten & T. Ranarilalatiana, MAD18-112 (1♂ NHRS); Alaotra-Mangoro, Zahamena NP Antanandava Sect., Manambato River by Camp Bemoara, 17.5126S, 48.7267E, 1050 m, 8-III-2018, leg. J. Bergsten & T. Ranarilalatiana, MAD18-94 (1♂ NHRS); Alaotra-Mangoro, Zahamena NP, Antanandava Sect., tributary stream (Sahavalanina) to Manambato River, 0.7 km NE Camp Bemoara, 17.5074S, 48.7310E, 1060 m, 7-III-2018, leg. J. Bergsten & T. Ranarilalatiana, MAD18-88 (2♂, 2♀ UMC; 4♂, 2♀, 6 nymphs NHRS); Analanjirofo Masoala NP, rainforest, pristine forest stream, 3 hours walk E of Andranobe camp, 15.6735S, 49.9886E, 500 m, 16-II-2018, leg. J. Bergsten & T. Ranarilalatiana, MAD18-44 (1♀, 4 nymphs NHRS).

## *Temnocoris leachi* NEW SPECIES

urn:lsid:zoobank.org:act:DA8491EC-9528-472B-9A97-8406A17E2745

(Fig 8)

### Forewing submacropterous male

**Fig 8:**
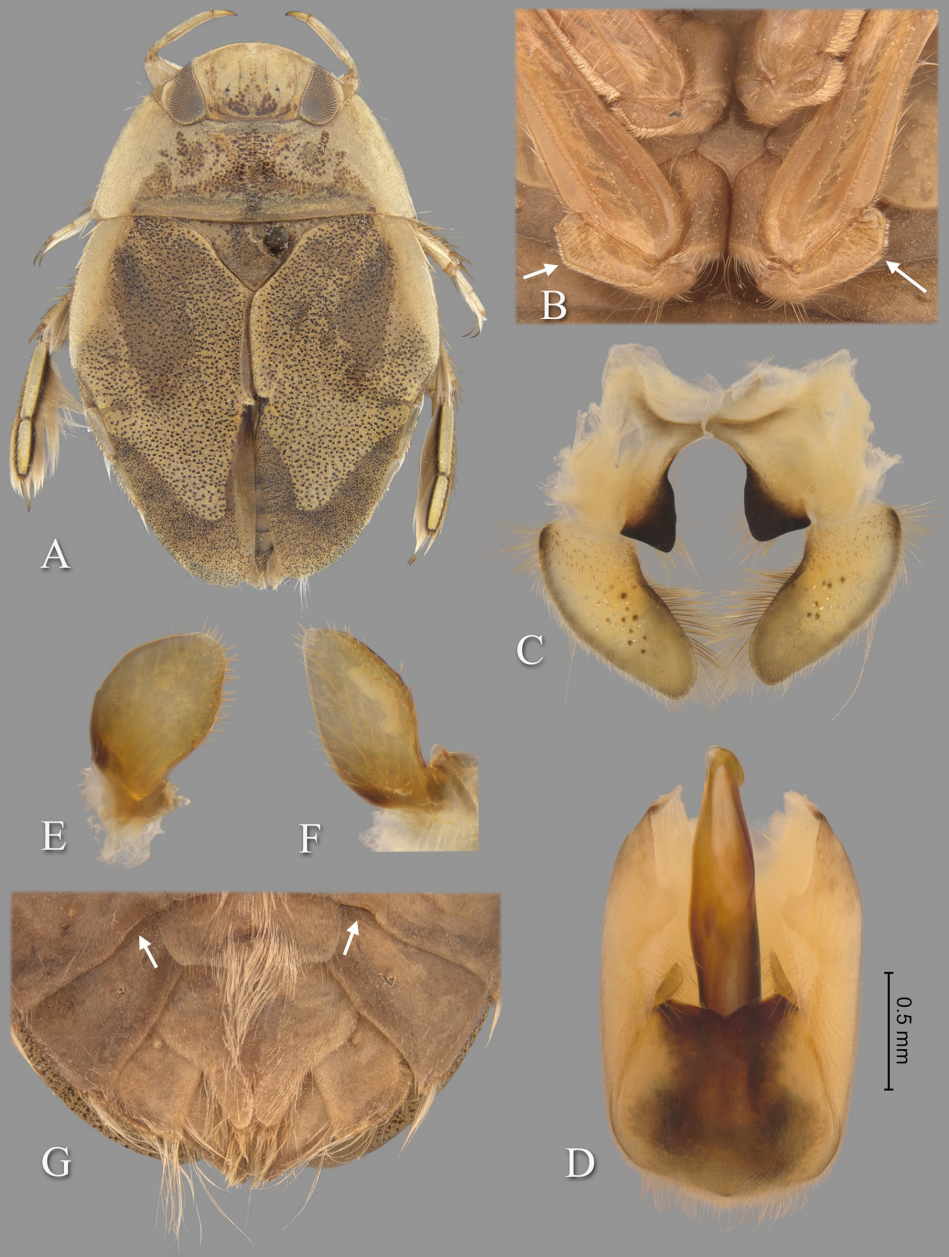
Temnocoris leachi n.sp. (A) Submacropterous female paratype, (B) bases of meso- and metathoracic legs, arrows indicate angled posterior margins, (C) male 8^th^ abdominal tergum, (D) male genital capsule with proctiger and tergum IX removed, (E) left paramere, (F) right paramere, (G) female subgenital plate, arrows indicate elevated posterior margins of laterosternites V forming pockets. Size bar pertains only to Fig D.

Holotype, length 10.24; maximum width across embolia 6.32. Paratypes (n = 1), length 9.52; maximum width 6.24. Overall shape elongate-oval. Dorsally, overall coloration light-brown with profuse black speckling on scutellum and hemelytra (Fig 8A); coarse punctation on pronotum. Ventrally, head, thorax, and legs pale-yellow; abdomen light-brown.

*Head*. length 1.72, maximum width 3.64, inner margin of eyes strongly divergent anteriorly. Synthlipsis at posterior margin 1.68; cuticle laterad of eye expanded and subtriangular. Vertex with coarse, brown punctation forming broad triangles on each side of midline, becoming confluent anteriorly; punctation flanked by black arcuate lines that are convergent anteriorly; dark-brown line of pigmentation extending from near posteromedian corner of eye to posteromedian margin of head. Anterior margin of head sharply emarginated and with fringe of erect setae between eyes; length of setae ca. 0.25× length of eye. Row of setae emanating from row of pits or sulcus paralleling inner margin of eyes. Labrum broadly triangular, distal margin broadly rounded, 1.96× wider than long. Antenna four-segmented, length 0.60, proportions 3, 12, 13, 6.

*Thorax*. Pronotum yellow, broad, 3.07× wider than long, length at midline 1.80; maximum width at posterolateral corners 5.52; coarse punctures in anterior 3/4, punctures brown in middle half, concolorous with yellow ground color in lateral 1/4; transverse band across posterior 1/4 devoid of coarse punctures; lateral margin evenly convex and with fringe of long setae, posterolateral corners bluntly acute, posterior margin nearly straight, anterior margin straight; ventrally, propleuron pale-yellow, mostly pruinose but with anterior and median lighter yellow area, anterior lighter yellow lobe extending posteriorly approximately to middle of procoxa. Scutellum surface irregular, triangular, yellow with black speckling, lateral margins distinctly sinuate, 2.02× wider than long, width 3.40, length 1.68. Hemelytra yellow with profuse black speckling throughout corium, clavus, membrane and posterior third of embolium; with sparse, fine, recumbent, pale setae. Embolium length 3.48, greatest width 1.00; lateral margin unevenly convex with degree of curvature greater near posterior end, with row of short spines and fringe of long setae. Claval suture present, but suppressed; intraclaval suture absent; claval commissure length 2.08. Membrane reduced, narrowly overlapping at midline, broadly V-shaped, extending anteriorly to claval commissure and along costal margin almost to embolium. Hindwing extending to tergum II. Metaxyphus broad, with acuminate apex. All leg segments pale yellow, protarsomere 2 and all pretarsi darker. Profemur elongate, wide, anterior margin with dorsal and ventral rows of golden hairs sandwiching one row of shorter, wide, brown setae. Distal 3/4 of protibia and two-segmented protarsus with dense ventral pad of setae. Propretarsus with short, stout, paired, movable claws. Mesofemur with anteroventral and mid-ventral rows of elongate setae; metafemur with anteroventral row of mixed short and long setae, a mid-ventral row of short, spinose setae, and posteroventral surface set with short spinules. Mesotrochanter and mesofemur with profuse brush of light colored setae on posterior margin; Metatrochanter with brush of setae less prevalent and with distinctly rounded angle on posterior margin (Fig 8B). Elongate, narrow, profuse pad of setae on mesotibia and mesotarsomeres 2 and 3. Long golden swimming hairs sparse on mesotibia and tarsus, profuse on metatibia and tarsus. Middle and hind legs with tarsomere 1 extending beneath tarsomere 2. Meso- and metathoracic pretarsal claws long, straight, with slight curvature apically. Leg measurements as follows: foreleg, femur 2.12, tibia 1.56, tarsomere 1 0.20, tarsomere 2 0.30; middle leg, femur 2.44, tibia 1.64, tarsomeres 1–3 0.16, 0.52, 0.52, pretarsal claws 0.58; hind leg, femur 2.96, tibia 2.56, tarsomeres 1–3 0.26, 1.42, 0.92, pretarsal claws 0.62.

*Abdomen*. Dorsally, terga II–VII yellow and lined with black on posterior margins; lateral margins of III–VII with regular row of short, stout, yellow spines and tuft of elongate setae at posterolateral corners. Tergum VIII with medial lobes (pseudoparameres) black, quadrate in appearance, with mesal margins convex, posteromedial corners narrowly rounded and posterolateral corners square or nearly so, width ca. 0.67× width of lateral lobes (Fig 8C). Ventrally with mid-ventral longitudinal band of elongate setae. Sterna II–V entire, VI–VII divided into medio- and laterosternites. Posterior margins of sterna III–IV symmetrical and nearly straight. Posterior margin of sternum V with median convexity directed to right, VI–VII symmetrical. Posterolateral corners of II–IV right angled, V acute. Aedeagus elongate, stout, widest near middle, gradually tapering apically, with apex rounded and deflected to right side (Fig 8D). Pygophore with anterior margin concave and broadly V-shaped between parameres, brush of long setae most prominent posteriorly (Fig 8D). Parameres asymmetrical; left paramere with all margins convex except posteromesal corner concave, elongate setae on distal half of dorsal surface and along mesal margin and anterior corner (Fig 8E); right paramere with mesal margin straight, anterior corner narrowly rounded, posteromesal corner convex, stout setae on most of dorsal surface (Fig 8F).

### Forewing submacropterous female

Paratypes (n = 4), length 8.32–8.96 (mean = 8.62); maximum width 5.52–5.76 (mean = 5.60). Similar to submacropterous male in general structure and coloration with following exceptions: Protarsus one-segmented. Pads of setae less pronounced on pro- and mesotibiae and tarsi. Sternum V divided into medio- and laterosternites. Laterosternite V with posterior margin elevated forming a pocket near mediosternite (Fig 8G). Posterior margins of all sterna symmetrical. Subgenital plate (mediosternite VII) lateral margins convex in basal half, becoming concave in apical half, terminating in a pair of apical flap-like lobes deeply cleft medially (lobes can be adjacent or separated), 1.21× wider than long, width 1.48, length 1.22 (measured to tip of apical lobe) (Fig 8G).

### Macropterous male

Paratypes (n = 2), length 10.32–10.40 (mean = 10.36); maximum width 6.04–6.08 (mean = 6.06). Similar to submacropterous male in general structure and coloration with following exceptions: Pronotum with posterolateral corners rounded. Hemelytra with membrane expanded and rounded distally, right broadly overlapping left. Claval suture distinct, intraclaval suture present. Hindwing extending to near middle of tergum VII.

### Macropterous female

Paratypes (n = 2), length 8.40–8.88 (mean = 8.64); maximum width 5.36–5.60 (mean = 5.48). Similar to submacropterous female in general structure and coloration with following exceptions: Pronotum with posterolateral corners rounded. Hemelytra with membrane expanded and rounded distally, right broadly overlapping left. Hindwing extending to near middle of tergum VII.

### Diagnosis

The angulate posterior margin of the metatrochanter in both sexes is similar to that of *Temnocoris poissoni* **n.sp.** The female subgenital plate with the distinct apical lobes also is similar to that of *Temnocoris poissoni* **n.sp.**, but the lobes are flap-like and the lateral margins are convergent at a much less severe angle (Fig 8G), and the right parameres of males of the two species are dramatically different. Also in the male, the shape of the aedeagus is distinct in that it tapers in the apical half to a rounded apex which appears deflected to the right (Fig 8D).

### Discussion

This species co-occurred with *Tsingala humeralis* in Mahajanga Province. We collected this species in slower water in two stream systems, including at the margin in pools.

### Etymology

The specific epithet honors William Elford Leach who worked in the Natural History Department of the British Museum in the early 1800s and is credited as the systematic authority for the family Naucoridae.

### Type material examined

HOLOTYPE hindwing brachypterous ♂: Madagascar: **Mahajanga:** Maropapango, River Maropapango under bridge RN6, -14.35419S, 48.01984E, 13 m, 18-XI-2012, leg. Bergsten, Bukontaite, Ranarilalatiana, Randriamihaja, MAD12-10 (NHRS). PARATYPES: same data (1 macropterous ♂, 1 brachypterous ♀ NHRS; 1 brachypterous ♂, 2 brachypterous ♀ UMC). **Fianarantsoa:** Ihorombe, R.S. Pic d’Ivohibe, Anefitany, Anefitany stream, S22°28’38.9", E046°57’49.4", 937 m, 8-XII-2013, pools and hole at river, leg. T. Ranarilalatiana & JH Randriamihaja MAD13-48 (1 brachypterous ♀, 1 macropterous ♀ NHRS; 1 macropterous ♀ UMC). **Mahajanga:** 16 km SE of Andriba, 8-XI-1986, JT & DA Polhemus, CL2271 (1 macropterous ♀ USNM).

### Other material examined

**Mahajanga:** Melaky, Tsingy de Bemeraha NP, S18.75724, E044.71239, 72 m, 17-XII-2009, leg. J. Bergsten, N. Jönsson, T. Ranarilalatiana, H.J. Randriamihaja, MAD09-63 (1♂ UMC).

## *Temnocoris montandoni* NEW SPECIES

urn:lsid:zoobank.org:act:CFCC539C-EC09-495D-8A91-D5D5E9935BB5

(Fig 9)

### Forewing submacropterous male

**Fig 9:**
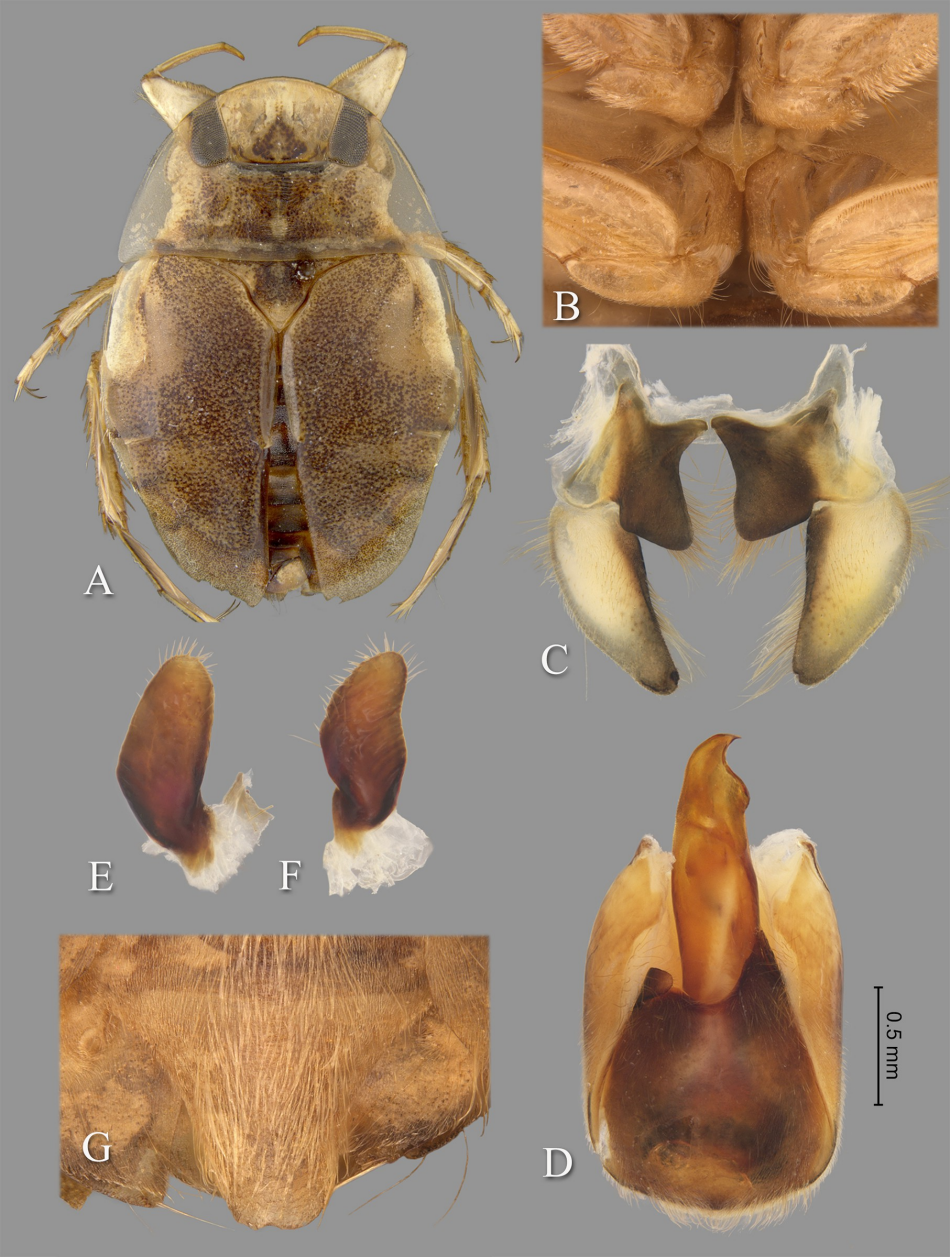
Temnocoris montandoni n.sp. (A) Macropterous female paratype, (B) bases of meso- and metathoracic legs, (C) male 8^th^ abdominal tergum, (D) male genital capsule with proctiger and tergum IX removed, (E) left paramere, (F) right paramere, (G) female subgenital plate. Size bar pertains only to Fig D.

Holotype, length 11.20; maximum width across embolia 6.96. Paratypes (n = 1), length 9.76, maximum width 6.80. Overall shape elongate-oval. Dorsally, overall coloration orange-yellow with profuse black speckling on hemelytra (Fig 9A); coarse punctation on pronotum. Ventrally, head, thorax, and legs yellowish; abdomen yellow with infuscation.

*Head*. length 1.84, maximum width 3.96, inner margin of eyes strongly divergent anteriorly. Synthlipsis at posterior margin 2.00; cuticle laterad of eye expanded and subtriangular. Vertex with coarse, brown confluent punctation forming broad, rounded triangles on each side of midline, triangular patches becoming confluent anteriorly; dark-brown markings near posteromedian corner of eye. Anterior margin of head sharply emarginated and with fringe of erect setae between eyes; length of setae ca. 0.25× length of eye. Row of setae emanating from row of pits or sulcus paralleling inner margin of eyes. Labrum broadly triangular, distal margin broadly rounded, 1.91× wider than long. Antenna apparently four-segmented, length 0.68, proportions 3, 13, 16, 7.

*Thorax*. Pronotum yellow, strongly tinged with orange in middle half, broad, 3.29× wider than long, length at midline 1.92; maximum width at posterolateral corners 6.32; coarse punctures in anterior 3/4 except anterolaterally, punctures brown in middle half, concolorous with yellow ground color in posterolateral 1/4; transverse band across posterior 1/4 devoid of coarse punctures; lateral margin evenly convex and with fringe of long setae, posterolateral corners bluntly acute and slightly extending posteriorly, posterior and anterior margins straight; ventrally, propleuron yellowish, mostly pruinose but with anterior and median lighter area, anterolateral lobe extending posteriorly approximately to middle of procoxa and infuscated posterolaterally. Scutellum surface irregular, triangular, yellow with black paramedian speckling, lateral margins distinctly sinuate, 2.61× wider than long, width 3.76, length 1.44. Hemelytra heavily suffused with brownish-orange and with profuse black speckling throughout corium, clavus, membrane and posterior third of embolium (speckling becoming confluent in some specimens); with sparse, fine, recumbent, pale setae. Membrane with profuse, fine, white punctures. Embolium length 3.40, greatest width 1.20; lateral margin almost straight to slightly convex for most of length, with degree of curvature greater near posterior end and humeral angle, with row of short spines and fringe of long setae. Claval suture indistinct; intraclaval suture absent; claval commissure length 2.20. Membrane reduced, narrowly overlapping at midline, broadly V-shaped, extending anteriorly to claval commissure and along costal margin almost to embolium. Hindwing extending to tergum I. Metaxyphus broad, with acuminate apex. All leg segments yellowish, protarsomere 2 and all pretarsi slightly darker. Profemur elongate, wide, anterior margin with dorsal and ventral rows of golden hairs sandwiching one row of shorter, wide, brown setae. Distal 3/4 of protibia and two-segmented protarsus with dense ventral pad of setae. Propretarsus with short, stout, paired, movable claws. Mesofemur with anteroventral and mid-ventral rows of elongate setae; metafemur with anteroventral row of mixed short and long setae, a mid-ventral row of short, spinose setae, and posteroventral surface set with short spinules. Mesotrochanter and mesofemur with profuse brush of light colored setae on posterior margin; Metatrochanter with brush of setae less prevalent and with posterior margin shallowly convex, without angle (Fig 9B). Elongate, narrow, profuse pad of setae on mesotibia and mesotarsomeres 2 and 3. Long golden swimming hairs sparse on mesotibia and tarsus, profuse on metatibia and tarsus. Middle and hind legs with tarsomere 1 extending beneath tarsomere 2. Meso- and metathoracic pretarsal claws long, straight, with slight curvature apically. Leg measurements as follows: foreleg, femur 2.64, tibia 1.78, tarsomere 1 0.30, tarsomere 2 0.30; middle leg, femur 2.72, tibia 2.04, tarsomeres 1–3 0.16, 0.66, 0.62, pretarsal claws 0.70; hind leg, femur 3.32, tibia 2.92, tarsomeres 1–3 0.26, 1.50, 1.08, pretarsal claws 0.70.

*Abdomen*. Dorsally, terga II–VII brownish-yellow and lined with black on posterior margins; lateral margins of III–VII with regular row of short, stout, yellow spines and tuft of elongate setae at posterolateral corners. Tergum VIII with medial lobes (pseudoparameres) black, with posteromedial corners narrowly rounded and posterolateral corners obtuse, width ca. 0.80× width of lateral lobes (Fig 9C). Ventrally with mid-ventral longitudinal band of elongate setae. Sterna II–V entire, VI–VII divided into medio- and laterosternites. Posterior margins of sterna III–IV symmetrical and nearly straight. Posterior margin of sternum V with median convexity directed to right with notch to left of midline, VI–VII symmetrical. Posterolateral corners of II–IV right angled, V acute. Aedeagus elongate and stout, left side slightly convex until broadly rounded apex, right side with subapical bulge basal to pronounced concavity, acuminate hook at apex (Fig 9D). Pygophore with anterior margin deeply, broadly, and asymmetrically U-shaped between parameres, brush of long setae most prominent posteriorly (Fig 9D). Parameres asymmetrical; left paramere with lateral and mesal margins nearly straight and shallowly convergent to broadly rounded apex, elongate setae near apex (Fig 9E); right paramere with lateral margin concave at middle, mesal margin convex, apex broadly rounded, elongate setae on mesal margin and apex (Fig 9F).

### Forewing submacropterous female

Paratypes (n = 3), length 9.20–10.08 (mean = 9.71); maximum width 6.15–6.56 (mean = 6.32). Similar to submacropterous male in general structure and coloration with following exceptions: Protarsus one-segmented. Pads of setae less pronounced on pro- and mesotibiae and tarsi. Sternum V divided into medio- and laterosternites. Posterior margins of all sterna symmetrical. Subgenital plate (mediosternite VII) lateral margins shallowly concave and convergent, terminating in a broadly rounded apex with small median notch, 1.36× wider than long, width 1.50, length 1.10 (Fig 9G).

### Macropterous male and female

Unknown.

### Diagnosis

The posterior margin of the metatrochanter is not angulate in either sex. In males, the shape of the aedeagus is distinct in that the apex is sharply hooked on the right side, which is immediately distal to a deep concavity and lateral bulge on the right (Fig 9D); the shape of the right paramere is also distinct (Fig 9F). The aedeagus and parameres are similar to those of *T*. *dubius*; however, the aedeagus is more sharply hooked and with a more pronounced subapical notch and bulge, and the paramere shapes are slightly different. The female subgenital plate lateral margins are shallowly concave and converge to a rounded apex with median notch (Fig 9G).

### Discussion

This species was collected at only one locality, Ambohitantely Special Reserve on the central highland plateau. Both males are distinctly brownish-orange in color, whereas females are more yellowish.

### Etymology

The specific epithet honors Arnold Lucien Montandon, the French naturalist who worked in the Grigore Antipa National Museum of Natural History in Bucharest, Romania, and described more than 400 species [31], including numerous species of Naucoridae.

### Type material examined

HOLOTYPE hindwing brachypterous ♂: Madagascar: **Antananarivo**: Analamanga, Ambohitantely special reserve, 18.1808S, 47.2901E, 1340 m, 22-XI-2014, forest stream with waterfalls and over bedrock, leg. J. Bergsten, R. Bukontaite, J.H. Randriamihaja, T. Ranarilalatiana, S. Holmgren, MAD14-76 (NHRS). PARATYPES: same data as holotype (1♂, 1♀ NHRS; 1♂, 1♀ UMC).

## *Temnocoris perplexus* Poisson, 1951

(Fig 10)

*Temnocoris perplexus* Poisson 1951: Mem. Inst. Sci. Madag. A5: 106–108 (original description).

*Temnocoris perplexus*: Poisson 1952, Mem. Inst. Sci. Madag. E1: 57–58, 60 (supplemental description, figures).

*Temnocoris perplexis* [sic]: La Rivers 1971, Biol. Soc. Nev. Mem. 2: 79 (catalog).

**Fig 10:**
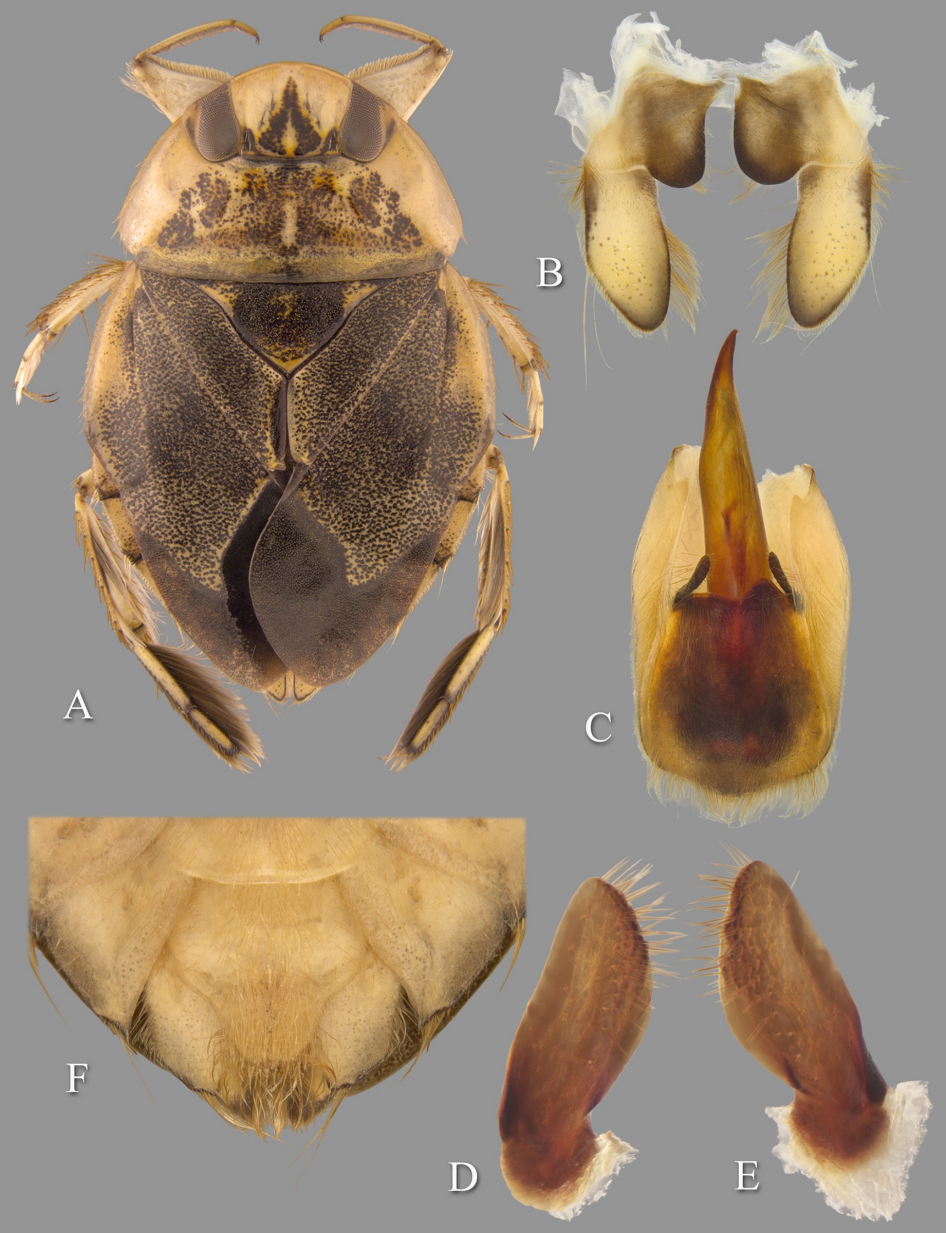
Temnocoris perplexus. (A) Macropterous female, (B) male 8^th^ abdominal tergum, (C) male genital capsule with proctiger and tergum IX removed, (D) left paramere, (E) right paramere, (F) female terminal abdominal sterna with subgenital plate.

### Discussion

The original description was based on one macropterous female [26]. The following year, based on two male and two female subbrachypterous specimens, Poisson [29] provided a supplemental description, including of the male parameres and aedeagus; however, labelling of the left and right parameres was reversed and the aedeagus figure was reversed horizontally. Because he slide-mounted these structures on a single glass slide, it is likely that he either examined the microscope slide upside-down or mounted the structures upside-down on the slide.

### Diagnosis

The aedeagus gradually becomes slender in the distal third, angles right, and ends in a tiny apical hook to the right (Fig 10C). The parameres are similarly shaped, although the length of the left paramere is 1.1× that of the right (Fig 10D and 10E). The pseudoparameres are broadly rounded (Fig 10B). The female subgenital plate is about as long as it is wide; the lateral margins are shallowly concave and converge to a broadly rounded apex (Fig 10F) and is similar in shape to those of *T*. *ambositrae* and *T*. *montandoni* **n.sp.**

### Published records

Ambodivoangy [26], Tananarive [29].

**Material examined**. **Antananarivo**: Tananarive, Bought, 1937, C. Lamberton (5♂, 6♀ SEMC). Tananarive, Bought 1937, C. Lamberton / Temnocoris perplexus Poisson / ♂ / Type / A. det. H.B. Hungerford. (♂ SEMC). Madagascar, Tananarive, Bought 1937, C. Lamberton / 1. [?esp.] A., d—Hung / ♂ / cotype / Temnocoris perplexus Poiss. / Poisson to Drake Coll 1979 (♂ USNM). **Antsiranana**: Marojejy National Park, 14°26.228’S, 49°46.549’E, elev. 459 m, 8-XI-2014, R.W. Sites, forest stream w/ marginal veg., L-1848 (1♂, 1♀ UMC).

## *Temnocoris poissoni* NEW SPECIES

urn:lsid:zoobank.org:act:70B4C278-EFA5-47FB-900C-105575CD73AF

(Figs 1B and 11)

### Forewing submacropterous male

**Fig 11:**
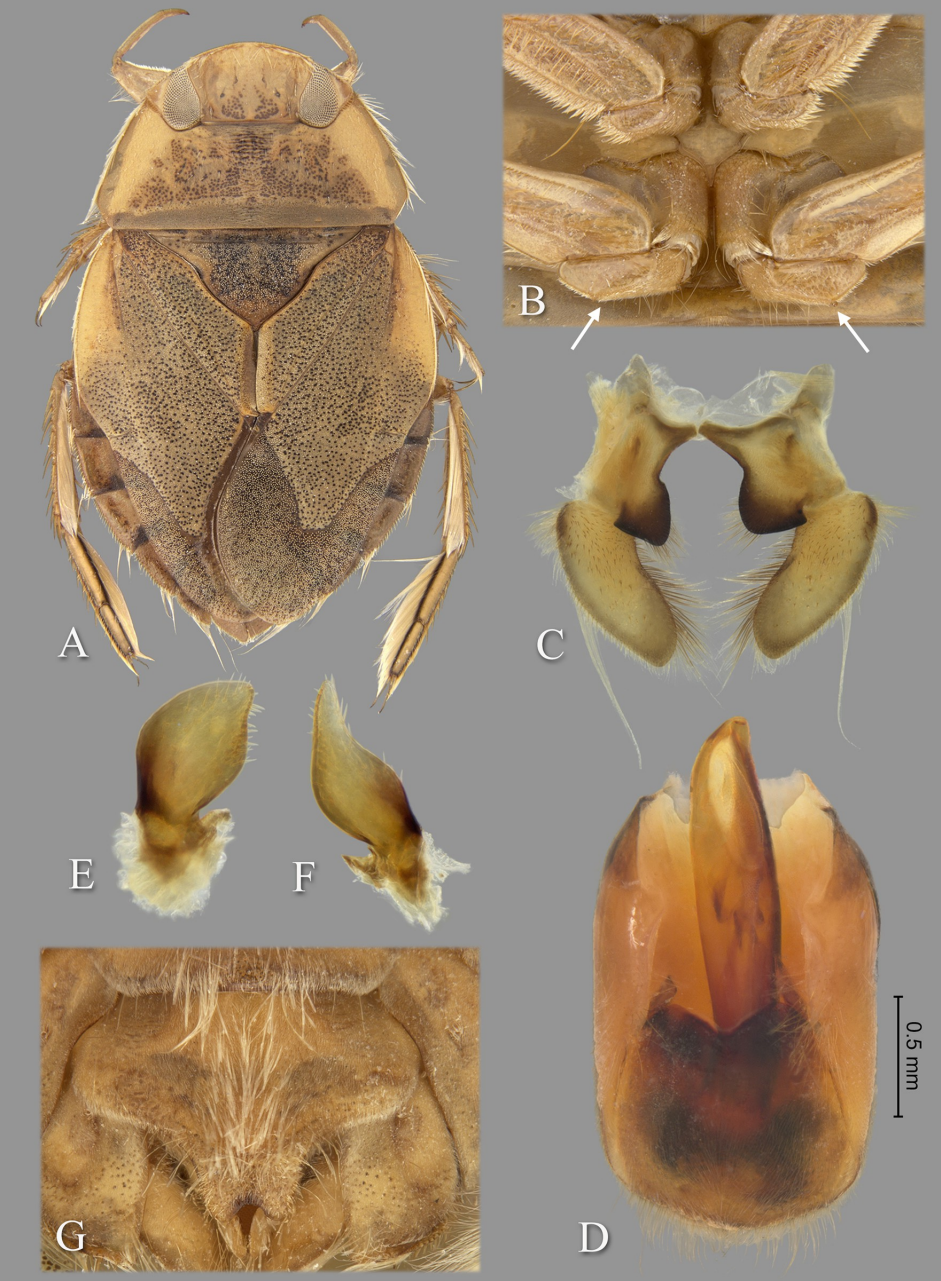
Temnocoris poissoni n.sp. (A) Macropterous female paratype, (B) bases of meso- and metathoracic legs, arrows indicate angled metatrochanters, (C) male 8^th^ abdominal tergum, (D) male genital capsule with proctiger and tergum IX removed, (E) left paramere, (F) right paramere, (G) female subgenital plate. Size bar pertains only to Fig D.

Holotype, length 10.24; maximum width across embolia 6.40. Paratypes (n = 5), length 9.20–10.48 (mean = 10.10); maximum width 6.20–6.56 (mean = 6.33). Overall shape elongate-oval. Dorsally, overall coloration light-brownish-yellow with profuse black speckling on hemelytra (as in Fig 11A); coarse punctation on pronotum. Ventrally, head and thorax yellowish; abdomen and legs light-brown.

*Head*. length 1.60, maximum width 3.64, inner margin of eyes strongly divergent anteriorly. Synthlipsis at posterior margin 1.68; cuticle laterad of eye expanded and subtriangular. Vertex with coarse, brown punctation forming broad, rounded triangles on each side of midline, becoming confluent anteriorly; dark-brown line of pigmentation extending from near posteromedian corner of eye toward posteromedian margin of head. Anterior margin of head sharply emarginated and with fringe of erect setae between eyes (Fig 1B); length of setae ca. 0.25× length of eye. Row of setae emanating from row of pits or sulcus paralleling inner margin of eyes. Labrum broadly triangular, distal margin broadly rounded, 1.85× wider than long. Antenna apparently four-segmented, length 0.56, proportions 3, 11, 11, 6.

*Thorax*. Pronotum yellow, broad, 3.05× wider than long, length at midline 1.86; maximum width at posterolateral corners 5.68; coarse punctures in anterior 3/4, punctures brown in middle half, concolorous with yellow ground color in lateral 1/4; transverse band across posterior 1/4 devoid of coarse punctures; lateral margin evenly convex and with fringe of long setae, posterolateral corners bluntly acute and slightly extending posteriorly, posterior margin nearly straight, anterior margin straight; ventrally, propleuron yellowish, mostly pruinose but with anterior and median darker yellow area, anterior dark yellow lobe extending posteriorly approximately to middle of procoxa. Scutellum surface irregular, triangular, yellow with black paramedian speckling, lateral margins distinctly sinuate, 2.42× wider than long, width 3.20, length 1.32. Hemelytra light-brownish-yellow with profuse black speckling throughout corium, clavus, membrane and posterior third of embolium; with sparse, fine, recumbent, pale setae. Membrane with profuse, fine, white punctures. Embolium length 3.40, greatest width 0.92; lateral margin almost straight to slightly convex for most of length, with degree of curvature greater near posterior end, with row of short spines and fringe of long setae. Claval suture present, but suppressed; intraclaval suture absent; claval commissure length 1.92. Membrane reduced, narrowly overlapping at midline, broadly V-shaped, extending anteriorly to claval commissure and along costal margin almost to embolium. Hindwing extending to tergum I. Metaxyphus broad, with acuminate apex. All leg segments light-brown, protarsomere 2 and all pretarsi darker. Profemur elongate, wide, anterior margin with dorsal and ventral rows of golden hairs sandwiching one row of shorter, wide, brown setae. Distal 3/4 of protibia and two-segmented protarsus with dense ventral pad of setae. Propretarsus with short, stout, paired, movable claws. Mesofemur with anteroventral and mid-ventral rows of elongate setae; metafemur with anteroventral row of mixed short and long setae, a mid-ventral row of short, spinose setae, and posteroventral surface set with short spinules. Mesotrochanter and mesofemur with profuse brush of light colored setae on posterior margin; Metatrochanter with brush of setae less prevalent and with distinct angle on posterior margin (Fig 11B). Elongate, narrow, profuse pad of setae on mesotibia and mesotarsomeres 2 and 3. Long golden swimming hairs sparse on mesotibia and tarsus, profuse on metatibia and tarsus. Middle and hind legs with tarsomere 1 extending beneath tarsomere 2. Meso- and metathoracic pretarsal claws long, straight, with slight curvature apically. Leg measurements as follows: foreleg, femur 2.42, tibia 1.60, tarsomere 1 0.24, tarsomere 2 0.32; middle leg, femur 2.56, tibia 1.74, tarsomeres 1–3 0.20, 0.56, 0.52, pretarsal claws 0.60; hind leg, femur 3.16, tibia 2.68, tarsomeres 1–3 0.30, 1.44, 1.04, pretarsal claws 0.68.

*Abdomen*. Dorsally, terga II-VII light-brown and lined with black on posterior margins; lateral margins of III–VII with regular row of short, stout, yellow spines and tuft of elongate setae at posterolateral corners. Tergum VIII with medial lobes (pseudoparameres) black, quadrate in appearance, with mesal margins convex, posteromedial corners rounded and posterolateral corners square or nearly acute, width ca. 0.75× width of lateral lobes (Fig 11C). Ventrally with mid-ventral longitudinal band of elongate setae. Sterna II-V entire, VI-VII divided into medio- and laterosternites. Posterior margins of sterna III–IV symmetrical and nearly straight. Posterior margin of sternum V with median convexity directed to right with abrupt concavity to left of midline, VI-VII symmetrical. Posterolateral corners of II–IV right angled, V acute. Aedeagus elongate, stout, left side nearly straight until broadly rounded apex (Fig 11D). Pygophore with anterior margin apparently symmetrical, deeply and broadly V-shaped between parameres, brush of long setae most prominent posteriorly (Fig 11D). Parameres asymmetrical; left paramere with anterior margin convex, mesal margin concave, elongate setae on distal half of dorsal surface and on ventral surface facing aedeagus (Fig 11E); right paramere with apical half acuminate, mesal margin straight, anterior margin sinuate, stout setae on margins at apex (Fig 11F).

### Forewing submacropterous female

Paratypes (n = 2), length 9.12–9.16 (mean = 9.14); maximum width 5.84. Similar to submacropterous male in general structure and coloration with following exceptions: Protarsus one-segmented. Pads of setae less pronounced on pro- and mesotibiae and tarsi. Metatrochanter with posterior angle less pronounced. Sternum V divided into medio- and laterosternites. Laterosternite V with posterior margin elevated forming a pocket near mediosternite. Posterior margins of all sterna symmetrical. Subgenital plate (mediosternite VII) lateral margins at angle greater than 45° to long axis of body in basal half (sometimes nearly horizontal), becoming concave in apical half, terminating in a pair of rigid apical lobes with a U-shaped concavity between (apical lobes tend to be reflexed dorsad), 1.43× wider than long, width 1.66, length 1.16 (measured to tip of apical lobe) (Fig 11G).

### Macropterous male

Paratypes (n = 4), length 9.76–10.80 (mean = 10.34); maximum width 6.16–6.40 (mean = 6.28). Similar to submacropterous male in general structure and coloration with following exceptions: Pronotum with posterolateral corners rounded. Hemelytra with membrane expanded and rounded distally, right broadly overlapping left. Claval suture distinct, intraclaval suture present. Hindwing extending to near middle of tergum VII.

### Macropterous female

Paratypes (n = 5), length 8.88–9.52 (mean = 9.06); maximum width 5.20–6.00 (mean = 5.64). Similar to submacropterous female in general structure and coloration with following exceptions: Pronotum with posterolateral corners rounded. Hemelytra with membrane expanded and rounded distally, right broadly overlapping left. Claval suture distinct, intraclaval suture present. Hindwing extending to near middle of tergum VII.

### Diagnosis

The angulate posterior margin of the metatrochanter, which is more pronounced in males, is similar to that of *Temnocoris leachi* **n.sp.** The female subgenital plate with the almost horizontal lateral margins is unique, and the rigid (not flap-like) apical lobes (Fig 11G) are similar to, but distinct from, those of *Temnocoris leachi* **n.sp.** (compare with Fig 8G). In males, the shape of the aedeagus is distinct in that the apex is broadly rounded on the left side (Fig 11D) and the right paramere being non-lanceolate, but with the apex acuminate, is unique among described species of *Temnocoris* (Fig 11F).

### Discussion

This species was collected on the sand in two unshaded, shallow, sandy streams, one with water temperature 106°F (= 41.1°C).

### Etymology

The specific epithet honors Raymond A. Poisson for his many contributions to the taxonomy of African aquatic Heteroptera in the middle of the 20th century.

### Type material examined

HOLOTYPE hindwing brachypterous ♂: Madagascar**: Toliara**: river 65 mi. SE of Morondava, 350’, 25-XI-1986, J.T. & D.A. Polhemus, CL2287 (USNM). PARATYPES: same data as holotype (3♂, 1♀ brachypterous, 2♀ macropterous USNM; 1♂, 1♀ brachypterous UMC); Toliara Prov., stream at Mahaboboka, 110 km NE of Tulear, 800’, 5-XII-1986, D.A. Polhemus, CL2301 (1♂ brachypterous, 3♂, 2♀ macropterous USNM; 1♂, 1♀ macropterous UMC). **Fianarantsoa**: Matsiatra, Ambony, Ranomafana NP, Namorona River, 4 km from Vohiparara, S21.24535, E047.39629, 1080 m, 30-X-2011, leg. J. Bergsten, R. Bukontaite, T. Ranarilalatiana, & J.H. Randriamihaja, MAD11-02 (2♂, 1♀ NHRS; 1♂, 1♀ UMC).

## *Temnocoris scarletti* Poisson, 1941

(Fig 12)

*Temnocoris scarletti* Poisson 1941: Bull. Soc. Zool. France 66: 331–335 (original description).

*Temnocoris scarletti*: Poisson 1952, Mem. Inst. Sci. Madag. E1: 58 (figures).

*Temnocoris scarletti*: La Rivers 1971, Biol. Soc. Nev. Mem. 2: 79 (catalog).

**Fig 12:**
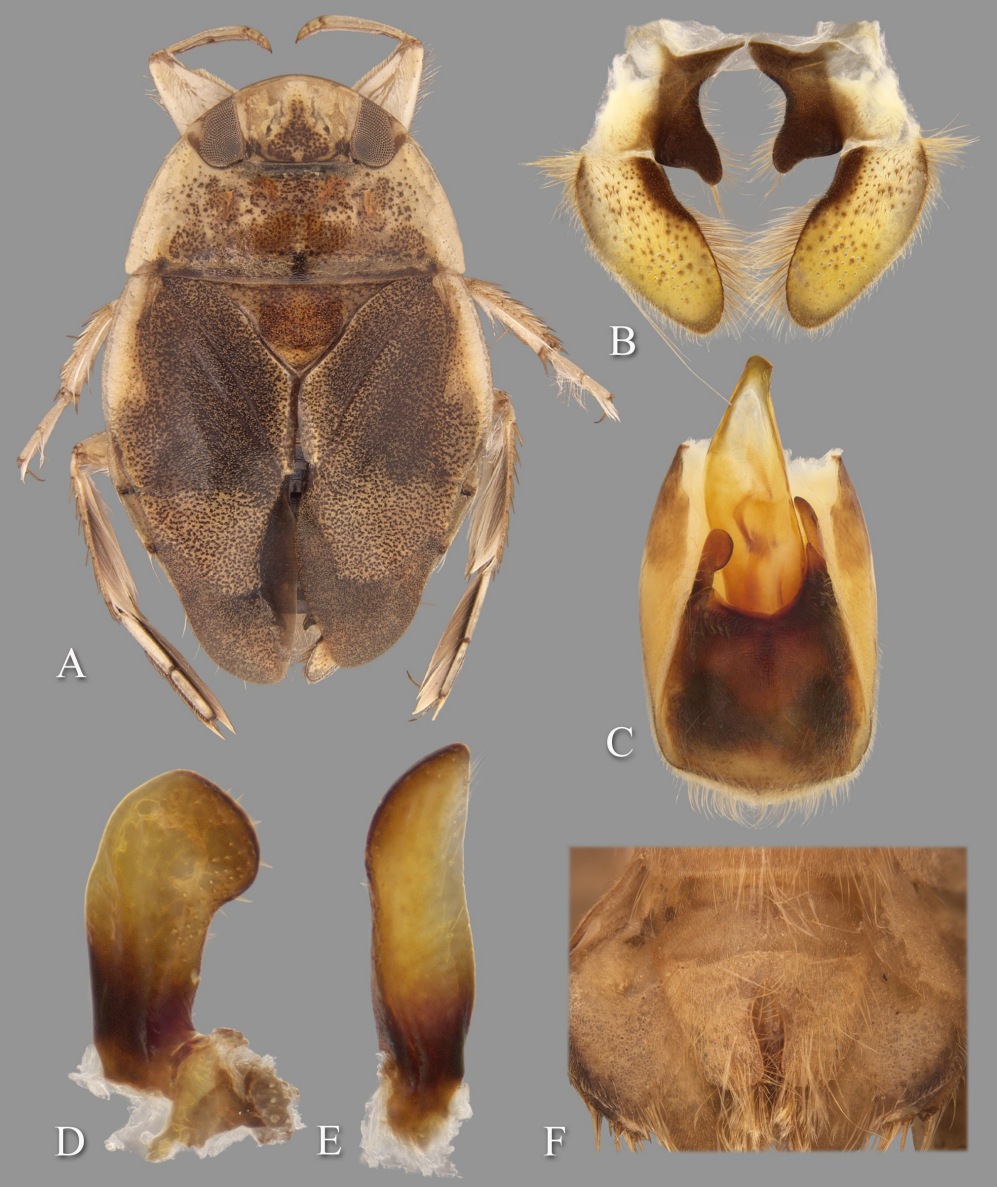
Temnocoris scarletti. (A) Submacropterous male, (B) male 8^th^ abdominal tergum, (C) male genital capsule with proctiger and tergum IX removed, (D) left paramere, (E) right paramere, (F) female subgenital plate.

### Discussion

Two forms of *T*. *scarletti* were described in the same paper [32]. Poisson based the species description on a single male macropterous specimen and the form *modestus* on two male and two female subbrachypterous specimens. Poisson (1941) clearly characterized *modestus* as the brachypterous form of *T*. *scarletti*; thus, although he described it prior to 1961 and gave a formal name, *modestus* is recognized only at an infrasubspecific level [33]. However, La Rivers (1971) incorrectly listed both forms as subspecies in his catalog. The type locality for *T*. *scarletti* was not given in the original description.

### Diagnosis

The left paramere with pronounced lobe in the distal half of the mesal margin (Fig 12C and 12D) and the pseudoparameres with the produced posteromesal lobes are unique and diagnostic in the male (Fig 12B). The female subgenital plate is deeply cleft with the cleft narrowly rounded at the anterior end and extending approximately half the length of the plate (Fig 12F).

### Published records

*T*. *scarletti modestus*: centre sud [32].

### Type material examined

HOLOTYPE ♂: *T*. *scarletti scarletti*: Madagascar [unknown locality] (slide-mounted genitalia USNM). *T*. *scarletti modestus*: Madagascar, centre sud / individual anormal / T. scarletti forme modestus nov. / Poisson to Drake Coll 1979 (USNM).

### Material examined

**Antsiranana**: 1.5 km north of Beraty village, -14.01654S, 48.26265E, 177 m, 21-XI-2012, leg. Bergsten, Bukontaite, Ranarilalatiana, Randriamihaja, MAD12-16 (1♂ NHRS); Daraina Reserve, Manakulana River, Ankijabe village, 13.2628S, 49.638E, 120 m, 1-XI-2014, pools in dry river, MAD14-34 (3 nymphs NHRS, 1♂ UMC); Andampinifosa village, 45 km N of Sambava on RN5A, Ifosa River crossing road, 14.0594S, 50.0264E, 40 m, 5-XI-2014, small shallow sandy river, leg. Bukontaite & Randriamihaja, MAD14-46 (1♂, 3 nymphs NHRS; 1♂, 1♀ UMC). **Unknown province**: N.W. Madagascar, 30-X-[19]07, J.J. Lloyd., 1908–193. (1♂ BMNH).

## *Temnocoris starmuhlneri* Poisson, 1962

(Fig 13)

*Temnocoris starmuhlneri* Poisson 1962b: Bull. Soc. Sci. Bretagne 37: 169–171 (original description).

*Temnocoris starmuhlneri*: La Rivers 1974, Biol. Soc. Nev. Occas. Pap. 38: 13 (catalog).

**Fig 13:**
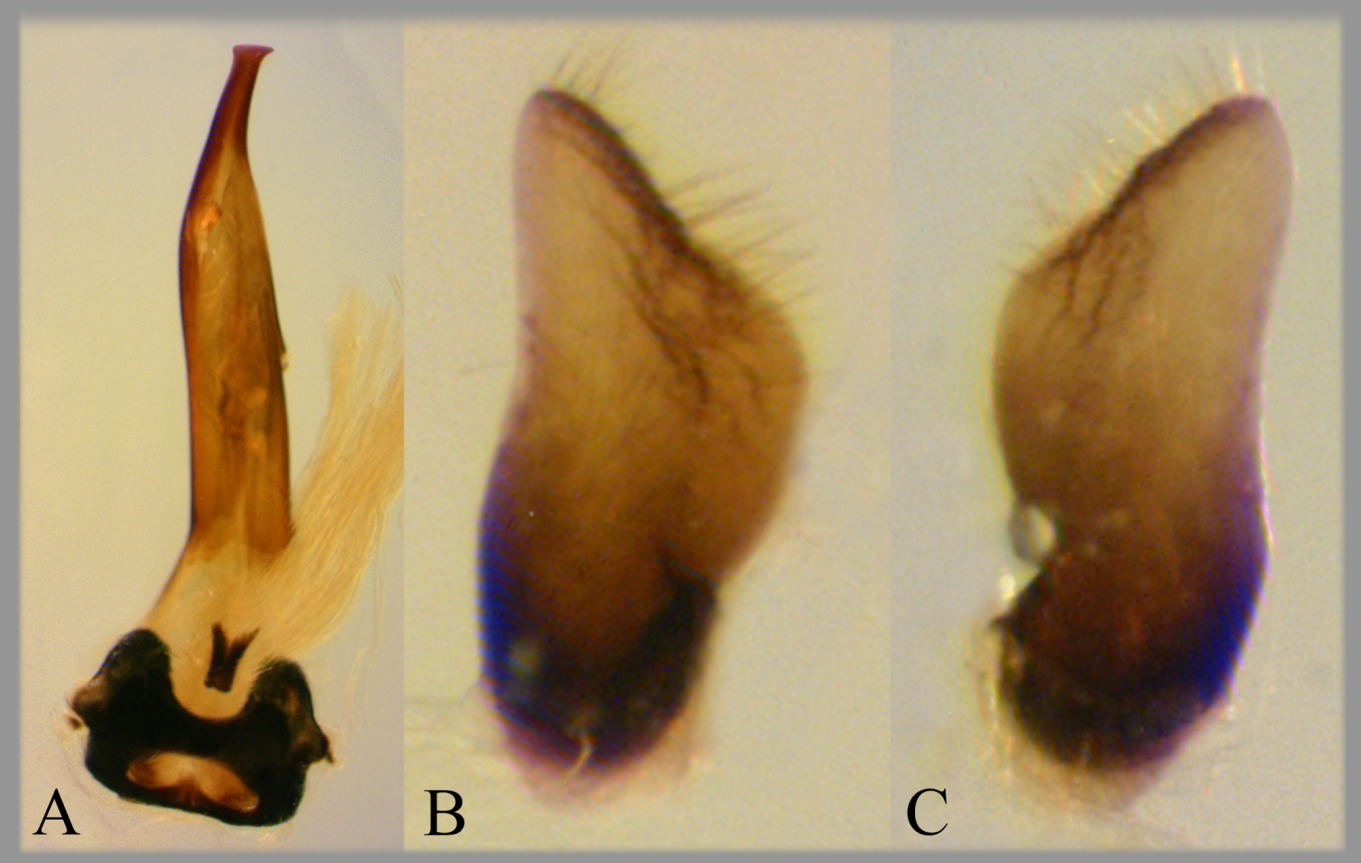
Temnocoris starmuhlneri. Male genitalia of holotype on Poisson slides (USNM). (A) Aedeagus, (B) left paramere, (C) right paramere.

### Discussion

This species is known from only a single male specimen. The description involved mostly color pattern, but Poisson [30] also gave measurements of the front leg and characteristics and figures of the parameres and aedeagus. He indicated that this species appears similar to *T*. *dubius*, but that it differs in the nearly symmetrical parameres and truncate aedeagus. He reported its length to be 14 mm and that this is the largest species of the genus [30]. Although we have not seen the type specimen, the slide-mounted aedeagus and parameres are housed in the Poisson slide collection in the USNM and photos of these structures are presented here (Fig 13A–13C). The parameres were not labeled on the slide; thus, left and right are interpreted here based on the figures and text in the original description [30].

### Diagnosis

The parameres are very similar in size and shape (Fig 13B and 13C), and the aedeagus is narrowed distally with a truncate apex and short hook (Fig 13A).

### Published records

Sakalava bach Ampamaherana [30].

### Type material examined

HOLOTYPE ♂: Sakalava bach Ampamaherana (slide-mounted genitalia USNM).

## *Temnocoris translucidus* Montandon, 1897

(Fig 14)

*Temnocoris translucidus* Montandon 1897b: Verh. Zool.-Bot. Gesel. Wien 47: 438–439 (original description).

*Temnocoris translucidus*: Poisson 1941, Bull. Soc. Zool. France 66: 329–331 (supplemental description).

*Temnocoris translucidus*: Poisson 1948, Mem. Inst. Sci. Madag. A1: 106 (distribution).

*Temnocoris translucidus*: Poisson 1951, Mem. Inst. Sci. Madag. A5: 108 (notes).

*Temnocoris translucidus*: Poisson 1956, Mem. Inst. Sci. Madag. E7: 256 (notes, distribution).

*Temnocoris translucidus*: La Rivers 1971, Biol. Soc. Nev. Mem. 2: 79 (catalog).

**Fig 14:**
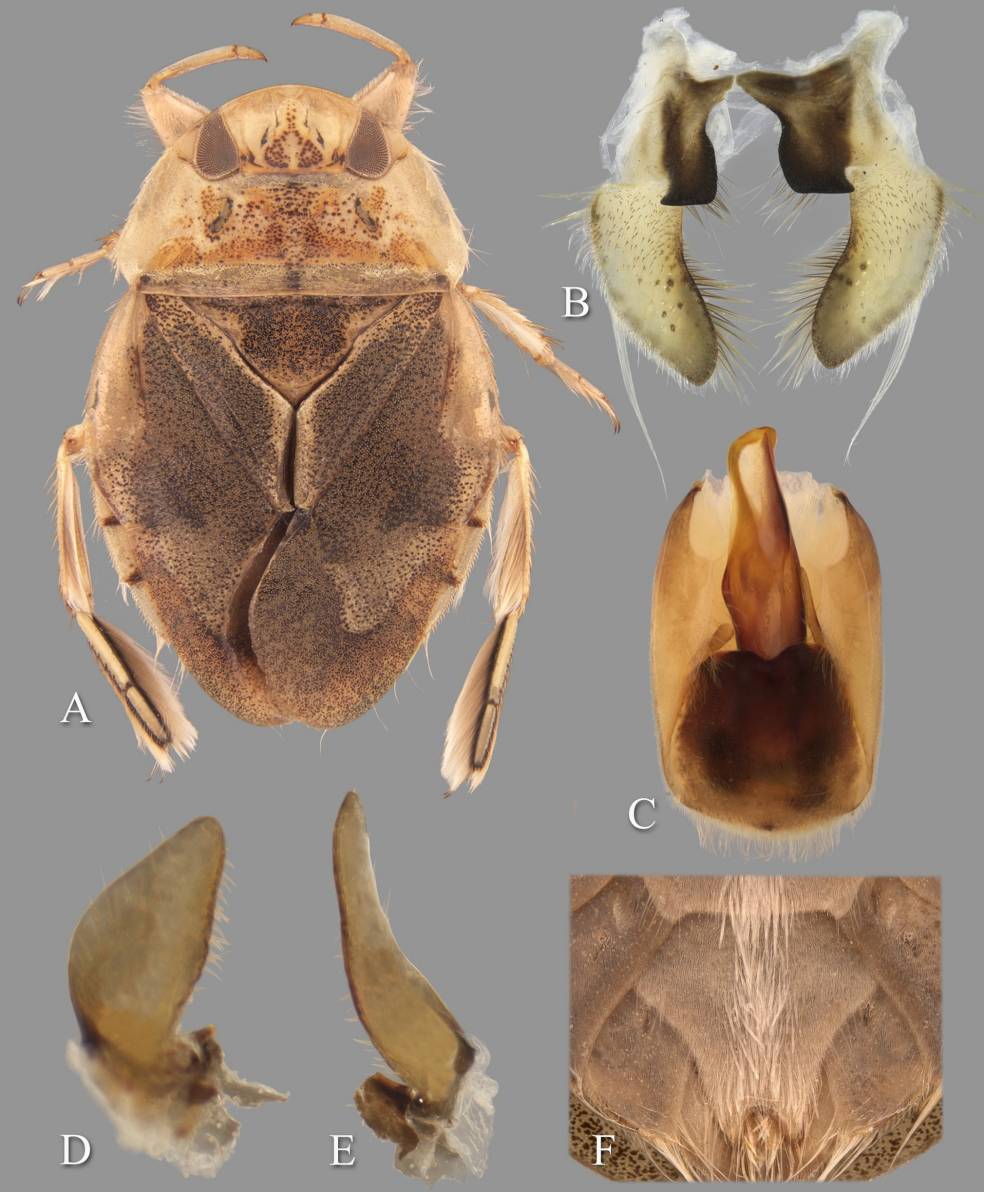
Temnocoris translucidus. (A) Macropterous male, (B) male 8^th^ abdominal tergum, (C) male genital capsule with proctiger and tergum IX removed, (D) left paramere, (E) right paramere, (F) female subgenital plate.

### Discussion

This is the type species of the genus and was described based on one specimen, which Montandon indicated was deposited in the MNHN; however, the specimen was not found there two recent visits. Following the original description by Montandon [25], Poisson [32] provided a supplemental description based on three subbrachypterous males, as well as distributional information and comparative notes [26,27,34]. As such, our concept of *T*. *translucidus* is based on the Poisson [32] supplemental description and genitalia slides in the USNM, which agree well with our specimens presented here.

### Diagnosis

This is a relatively small species and comparatively lighter in pigmentation than congeners. Our specimens measured 7.68–10.56 (mean = 8.61) and Poisson [32] gave the size range as 10.5–12 mm. Poisson [26] reported the color to be the same as that of *T*. *dubius* and *T*. *scarletti*. The eyes are noticeably more narrowed anteriorly (Fig 14A) than in congeners [26]. The male genitalia and pseudoparameres are diagnostic; the shape of the aedeagus (Fig 14C) is readily recognizable and the parameres are dramatically asymmetrical, with the left paramere short and subtriangular (Fig 14D, whereas the right paramere is slender, elongate, and gently curved (Fig 14E). The pseudoparameres are darkly colored and the posterolateral corners produced laterally to form a hooklike shape (Fig 14B). The female subgenital plate lateral margins are shallowly concave and converge to a shallowly to deeply concave apex (Fig 14F).

### Published records

Ambohivoangy [34], centre sud [32], Grande Forest [27].

### Material examined

All specimens macropterous unless otherwise noted. **Antsirinana**: Sava, 13 km SW of Vohemar along RN5A, Fananbe River, Mananbery village, 13.4451S, 49.9408E, 20 m, 5-XI-2014, dried out sandy river, leg. R. Bukontaite & J.H. Randriamihaja, MAD14-44 (1♀ subbrachypterous, 1♀ macropterous NHRS; 2♀ UMC); Diana, Mananjeba River, 17 km E of Ambilobe along RN 5A, 13.2071S, 49.1605E, 50 m, 31-X-2014, sandy river, leg. R. Bukontaite & J.H. Randriamihaja, MAD14-32 (10♂, 1♀ subbrachypterous, 11 ♀ macropterous, 1 nymph NHRS; 9♂, 1♀ subbrachypterous, 14 ♀ macropterous UMC; DNA extracted—1♀ UMC); Diana, Ambakirano River, 15 km E of Ambilobe along RN 5A, 13.215S, 49.1547E, 50 m, 31-X-2014, sandy river under bridge, Bukontaite & Randriamihaja, MAD14-31 (1♂, 1♀ NHRS); Diana, 5 km E of Ambilobe along RN 5A, 13.2076S, 49.0733E, 0 m, 31-X-2014, stream under bridge with sand and algae, Bukontaite & Randriamihaja, MAD14-29 (2♀ NHRS). **[Mahajanga Province]**: Marovoay Western Madagascar Prov., Majunga River, Ikopa, 1927 & 28 (2♀ SEMC); same data but also with Temnocoris det. H.B. Hungerford / R. Poisson det., Temnocoris translucidus ♀ Mont. Poiss. 1941 (1♀ SEMC). **Toliara**: Beraketa, So. Thosy / Temnocoris translucidus Mont., Poisson to Drake Coll. 1979 (1♂ USNM); Atsimo Andrefana, river 1 km north of Befandriana Sud, 22.08445S, 43.88558E, 134 m, 27-XI-2013, pools with vegetation next to river, leg. T. Ranarilalatiana & J.H. Randriamihaja, MAD13-26 (1♂ NHRS). **Unknown province:** Great Oriental Forest / Temnocoris translucidus Mont., c. cotype, det. by H.B. Hungerford / R. Poisson det. 1955, Temnocoris translucidus Mont. (1♂ SEMC).

### Genus *Tsingala* Sites, 2022

*Tsingala* Sites 2022: Zool. J. Linn. Soc. 195: 1272–1273. Type species: *Tsingala humeralis* (Signoret, 1860), by original designation.

*Tsingala* was recently erected to contain the Madagascar species formerly held in *Heleocoris* Stål, 1876, which was shown to be polyphyletic with three distinct, independent clades [21]. Because the type species of *Heleocoris* is *H*. *obliquatus* Spinola, which is in a clade with Indian species, *T*. *humeralis* was designated as the type species of *Tsingala* [21]. *Tsingala* can be distinguished from *Temnocoris* by the anterior margin of the head, which is bullnosed and hairless in *Tsingala* (Fig 1C), angulate and hairless in *Gonioathrix*, and sharply margined and with a fringe of hairs in *Temnocoris*.

*Tsingala* is an exceptionally challenging genus with which to work at the species level. Features given by Montandon [25] and Poisson [26,35] are problematic and include the inner margins of the eyes, which was given as barely vs. noticeably convergent, but was not quantified. Pronotum proportions using the anterior and posterior widths were each compared to the middorsal length, but the reported proportions are not close to our critical measurements of the available syntypes. Paramere shapes provided in line drawings by Poisson depict intraspecific variation, although differences among species are evident. Dorsal coloration is exceptionally variable within species and is generally not taxonomically reliable. Four new species are reported here and were validated as distinct species by both the recent molecular phylogeny of the family [21] and by morphological attributes, some of which were unreported by previous authorities, including shape of the female subgenital plate (abdominal sternum VII), which we have found to be diagnostic for three of the four new species described here. Thus, features we consider taxonomically informative include parameres, female mediosternite VI, female subgenital plate, overall size, and ventral coloration. Some species are characterized by features unique to males, females, or both. Further, because certain species require either males, females, or both for identification, collectors should endeavor to collect series of specimens to ensure that both sexes are represented; however, it is common for more than one species to be present at a site, which can further confound identification.

## *Tsingala angulata* NEW SPECIES

urn:lsid:zoobank.org:act:2E84261B-65E8-42D1-A956-3769B770E94C

(Fig 15)

### Macropterous female

**Fig 15:**
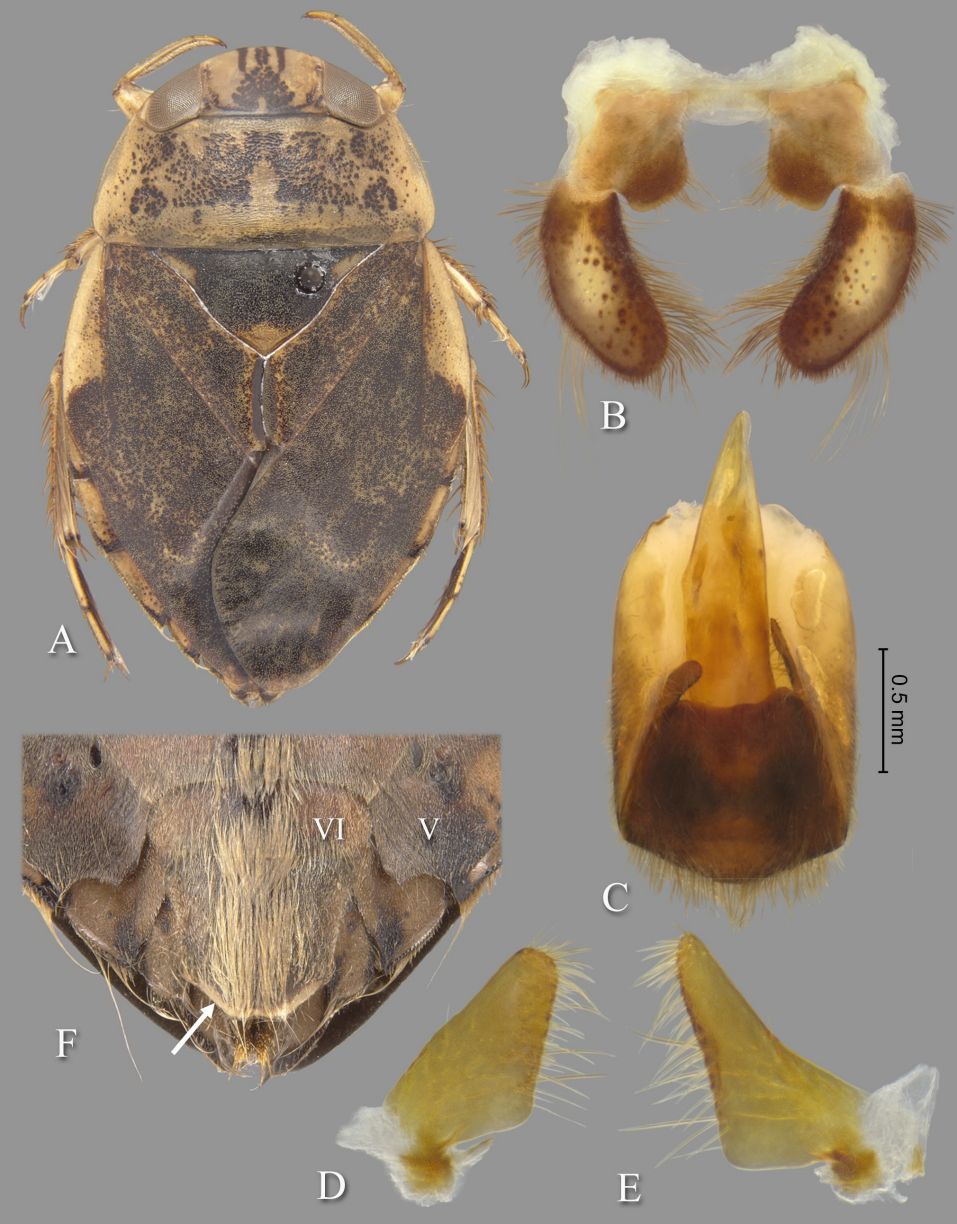
Tsingala angulata n.sp. (A) Holotype macropterous female, (B) male 8^th^ abdominal tergum, (C) male genital capsule with proctiger and tergum IX removed, (D) left paramere, (E) right paramere, (F) female terminal abdominal sterna with subgenital plate, arrow indicates angled posterolateral margin of subgenital plate, V = laterosternite V, VI = mediosternite VI. Size bar pertains only to Fig C.

Holotype, length 9.60; maximum width across embolia 5.84. Paratypes (n = 10), length 9.20–10.00 (mean = 9.46); maximum width 5.28–5.92 (mean = 5.70). Overall shape elongate-oval. Dorsally, overall coloration dark-brown with yellowish head, pronotum, proximal 2/3 of embolium (Fig 15A); brown, coarse punctation on head and pronotum; hemelytra irregularly brown, yellow, and finely punctate. Ventrally, abdomen light-brown, thorax yellow and medium-brown, legs yellowish.

*Head*. length 1.28, maximum width 3.76; inner margin of eyes slightly convergent anteriorly. Synthlipsis at anterior margin 1.72, interocular distance at posteromesal corner 1.96; cuticle laterad of eye expanded. Vertex with heavy, brown punctation forming broad triangle interrupted by yellow posteromedian gap; punctation extending anteriorly as irregular, double median row of coarse punctation, flanked by dark, paramedian row of coalescent punctation; irregular row of setae marked by broken line of brown pigmentation bordering inner margin of eyes; dark-brown line of pigmentation extending from near posteromedian corner of eye to posteromedian margin of head. Labrum with basal sulcus, transverse, distal margin evenly rounded, 2.07× wider than long. Antennae four-segmented, length 0.68, proportions 3, 12, 13, 8.

*Thorax*. Pronotum yellow, scabrous, broad, 2.48× wider than long, length at midline 2.00; maximum width at posterolateral corners 4.96; transverse band across posterior 1/4 devoid of coarse punctures; large, brown punctures tending to coalesce near midline, becoming smaller and more sparse laterally, with distinct yellow impunctate subrectangular area on midline anterior to transverse band; lateral margin evenly convex, posterolateral corners rounded, posterior margin shallowly convex, anterior margin straight; ventrally, propleuron generally medium-brown except yellow lateral glabrous area. Scutellum finely punctate, triangular, black with yellow apically and along lateral margins near anterior corners, with distinctly sinuate lateral margins, 2.13× wider than long, width 3.32, length 1.56. Hemelytra finely punctate throughout; with sparse, short, golden setae on corium extending throughout membrane; cuticle irregularly minimized creating dark-brown and yellow color differences and the appearance of small tubercles. Claval commissure length (to locking mechanism) 1.20. Embolium length 3.40 (chord measurement), greatest width 0.84; lateral margin evenly rounded; yellowish with scattered brown punctures in anterior 2/3, brown in posterior third. Hindwing extending to near middle of tergum V. Metaxyphus broad, with sharp mid-ventral ridge and papillose apex. Coxae brownish-yellow, all other leg segments yellow. Profemur elongate, inflated, anterior margin with dorsal and ventral rows of golden hairs sandwiching one row of short, dark spines. Distal 2/3 of protibia and single-segmented protarsus with dense ventral pad of setae. Propretarsus with short, stout, paired, movable claws. Meso- and metafemora with anteroventral and mid- or posteroventral rows of spinose setae; anteroventral seta rows arcuate from ventral surface to anterior margin, ending in distal fourth; setae number 23–25 on mesofemur and 30–33 on metafemur; mesofemur midventral row with 54–57 setae, with the distalmost 14–15 setae spaced distinctly more tightly; metafemur posteroventral row with 44–46 stout setae. Mesotrochanter and mesofemur with profuse brush of light colored setae on posteroventral margin; brush of setae narrowly set on metatrochanter and metafemur. Elongate, narrow pad of setae on mesotibia and mesotarsomeres 2 and 3. Long golden swimming hairs sparse on mesotibia and tarsus, profuse on metatibia and tarsus. Middle and hind legs with tarsomere 1 extending beneath tarsomere 2. Pretarsal claws long and with slight, even curvature. Leg measurements as follows: foreleg, femur 2.30, tibia 1.44, tarsus 0.44; middle leg, femur 2.32, tibia 1.64, tarsomeres 1–3 0.18, 0.48, 0.48; hind leg, femur 2.84, tibia 2.80, tarsomeres 1–3 0.26, 0.98, 0.80.

*Abdomen*. Dorsally with lateral margins of II–VIII with regular row of short, stout, golden-brown spines. Terga with lateral margins of II–VII each yellow anteriorly, dark brown at posterolateral corner. Ventrally with dense mid-ventral band of elongate setae beginning on III, continuing and becoming more profuse to and including subgenital plate. Posterior margins of mediosternites III–VI symmetrical and nearly straight. Posterior margin of laterosternite V produced at middle with narrowly rounded apex, distal half concave. Posterolateral corners of mediosternite VI produced posteriorly as blunt tabs. Subgenital plate (mediosternite VII) lateral margins slightly convergent with angle of incidence of ~10% in basal 2/3, then increasing to ~60% in distal 1/3, to short, shallowly concave, truncate apex; 1.15× wider than long, width 1.38, length 1.20 (Fig 15F).

### Macropterous male

Paratypes (n = 10), length 7.84–9.60 (mean = 9.36); maximum width 4.80–5.60 (mean = 5.55). Similar to female in general structure and coloration with following exceptions: Protarsus two-segmented. Pads of setae more pronounced on pro- and mesotibiae and tarsi. Posterior margins of mediosternite III–IV straight, V symmetrical and deeply concave, VI asymmetrical, shallowly concave, and more expansive on left side, VII symmetrical and straight. Aedeagus elongate, stout, parallel sided and widest in basal half, left lateral margin in distal half straight and angled toward apex, right lateral margin gently convex in distal half until apex, apex slightly deflected to right (Fig 15C). Pygophore with anterior margin concave between parameres, slight asymmetry in anterior production of lateral lobes, sometimes with incipient intermediate lobe, brush of long setae most prominent posteriorly (Fig 15C). Parameres asymmetrical; left paramere with margins generally straight, posteromesal corner obtusely angled, anterior corner acutely angled, both corners broadly rounded, elongate setae extending mesad from almost entire mesal margin and anterior corner (Fig 15D); right paramere with lateral margin subtly angled creating broadly concave appearance, posterior and mesal margins straight, posteromesal corner narrowly rounded and right angled, anterior corner produced to a blunt point, elongate setae extending mesad from almost entire mesal margin and anterior corner (Fig 15E).

### Diagnosis

This species is characterized by the shape of the female subgenital plate, in which the lateral margins are markedly angled at 2/3 length to become noticeably more convergent, and terminating in a short, truncate apex with a shallow concavity (Fig 15F). The hemelytra are punctulate and with the integument variably expressed with the surface continuously normally sclerotized or the sclerotization minimized creating lighter areas; thereby giving many specimens a speckled appearance (Fig 15A). Also, sparse, short, golden hairs of the hemelytra extend throughout the membrane. The abdominal sterna usually are orangish-brown.

### Discussion

Although the speckled appearance of the hemelytra can be seen on *T*. *angulata* **n.sp.**, other species can also have this appearance. Because the parameres are not sufficiently uniquely shaped to be diagnostic, males should be associated with identifiable females. We collected this species in a variety of lotic situations, including among vegetation at the margins of small streams, rock pools of a waterfall, and a silty, vegetated slow area of a large river. In the recent phylogeny of the family [21], this species was given as *Tsingala* sp. C and was sister to *Tsingala trilobata* **n.sp.**

### Etymology

The specific epithet *angulata* (= angular, cornered), a Latin adjective, is in reference to the angled posterolateral margins of the female subgenital plate.

### Type material examined

HOLOTYPE ♀: Madagascar: **Fianarantsoa** Province: Ranomafana National Park, Namorona River at Namorona Village, 21°15.738 S, 47°27.264’ E, elev. 621 m, 2-XI-2014, R.W. Sites, silty with cobble & marginal grasses, L-1830 (1♀ UMC). PARATYPES: **Fianarantsoa:** same data as holotype (2♂, 3♀ UMC); Ranomafana National Park, 21°15.494’S, 47°25.284’E, elev. 943 m, 3-XI-2014, seeps & hygropetric areas at Namorona River, R.W. Sites, K. Miller, S. Holmgren, L-1838 (13♂, 1♀ UMC); Ranomafana National Park, 21°15.477’S, 47°25.289’E, elev. 898 m, 3-XI-2014, rock pools in boulders, R.W. Sites, L-1835 (1♀ UMC). **Antsiranana**: Sava, Marojejy National Park, 14°28.088’S, 49°48.143’E, elev. 142 m, 7-XI-2014, small forest stream, R.W. Sites, L-1847 (1♀ UMC); Sava, Marojejy National Park, Humbert Waterfall, 14°25.970’S, 49°46.388’E, elev. 519 m, 8-XI-2014, rock pools, R.W. Sites, L-1849 (2♀ UMC); Sava, Marojejy NP, Humbert Waterfall, 14.4333S, 49.773E, 550 m, 8–12-XI-2014, leg. J. Bergsten, R. Bukontaite, J.H. Randriamihaja, T. Ranarilalatiana, S. Holmgren, MAD14-48 (1♀ NHRS); Sava, Marojejy National Park, 14.4345S, 49.7606E, 710 m, 9-XI-2014, hygropetric rocks and rockpools in wide river, leg. Bergsten, Bukontaite, Ranarilalatiana, Randriamihaja, Holmgren, MAD14-54 (1♀ NHRS); km NW Antsana, Galoko mountains, 13.60974S, 48.72175E, 263 m, 25-XI-2012, leg. Bergsten, Bukontaite, Ranarilalatiana, Randriamihaja, MAD12-26 (4♂, 1♀ NHRS; 3♂, 1♀ UMC); Daraina Reserve, Antsahabe River, Ankijabe village, 13.2639S, 49.6346E, 130 m, 1-XI-2014, leg. Bukontaite & Randriamihaja, MAD14-35 (1♀ UMC); Daraina Reserve, Antsahabe Waterfall, Ankijabe village, 13.276S, 49.6119E, 450 m, 3-XI-2014, leg. Bukontaite & Randriamihaja, MAD14-41 (1♀ NHRS); Anjanaribe Sud NP, River Marolakan, 14.7623S, 14.7623S, 920 m, 15-XI-2014, large rocky river, leg. J. Bergsten, R. Bukontaite, J.H. Randriamihaja, T. Ranarilalatiana, S. Holmgren, MAD14-64 (1♀ NHRS). **Toamasina**: Antsinanana, RN2, Laroka River, Ampasimbola, S18.96838, E048.73436, 240 m, 13-XI-2011, leg. J. Bergsten, R. Bukontaite, T. Ranarilalatiana, & J.H. Randriamihaja, MAD11-44 (1♂, 1♀ UMC).

### Additional material examined

**Fianarantsoa** Province: Ranomafana National Park, Namorona River at Namorona Village, 21°15.738S, 47°27.264’E, elev. 621 m, 4-XI-2014, R.W. Sites, marginal overhanging grasses in current, L-1843, DNA extracted (1♀ UMC).

## *Tsingala humeralis* (Signoret, 1860)

(Fig 16)

*Naucoris humeralis* Signoret 1860: Ann. Soc. Ent. France 8: 969.

*Ilyocoris humeralis*: Stål 1865, Hemip. Afr. 3: 175.

*Naucoris humeralis*: Walker 1873, Cat. Spec. Hemip. Heterop. Coll. Brit. Mus. 184.

*Heleocoris humeralis*: Stål 1876, K. Sven. Vetenskapsakad. Handl. 14: 146.

*Heleocoris humeralis*: Montandon 1897b, Verh. K. K. Zool.-Bot. Gesell. Wien 15: 445–446 (key).

*Heleocoris humeralis*: Poisson 1948, Mem. Inst. Sci. Mad. Ser. A: 1: 106.

*Heleocoris humeralis*: Poisson 1951, Mem. Inst. Sci. Madag. 5: 101.

*Heleocoris humeralis*: Poisson 1962a, Bull. Soc. Sci. Bret. 37: 39–40.

*Heleocoris humeralis*: Poisson 1963, Bull. Inst. Fr. Afr. Noire 25: 1181–1182.

*Heleocoris humeralis*: La Rivers 1971, Biol. Soc. Nev. Mem. 2: 78 (catalog).

*Tsingala humeralis*: Sites 2022, Zool. J. Linn. Soc. 195: 1272–1273.

**Fig 16:**
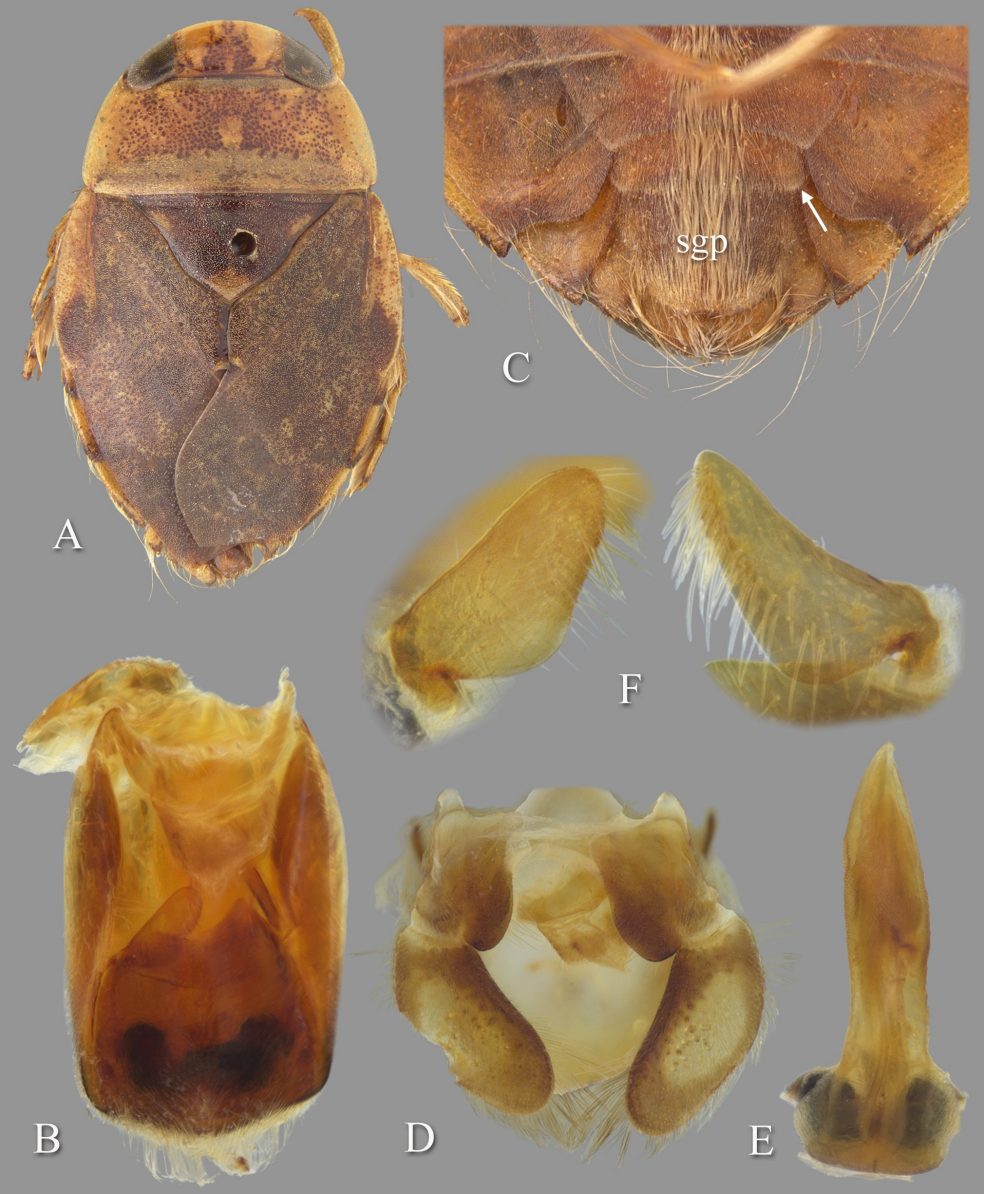
Tsingala humeralis. (A) Lectotype macropterous male, (B) genital capsule of paralectotype, (C–F) Montandon-determined specimens (USNM), (C) abdominal sterna of female, arrow indicates rounded posterolateral corner of mediosternite VI, (D) 8^th^ abdominal tergum, (E) aedeagus, (F) left and right parameres. sgp = subgenital plate.

### Lectotype designation

We examined two syntype males on loan from MCSN and Montandon-determined specimens from MNHN and USNM. One of the MCSN syntypes had historical dermestid damage; thus, we dissected the genitalia from this (Fig 16B) and the male USNM specimen. The parameres and other features are consistent between these dissected males; thus, we are confident that the Montandon-determined females at USNM (Fig 16C) can be associated as conspecific with the male syntypes. The undissected syntype male at MCSN is here designated as the lectotype (Fig 16A) in order to fix the identity of the species. The dissected male with dermestid damage at MCSN is a paralectotype.

### Diagnosis

This is the smallest species of *Tsingala*; body length was given as 8 mm [20] and 8–9 mm [35], but our specimens measure 7.28–9.04 mm. The female subgenital plate is broadly rounded, can have a slight median notch (Fig 16C), and is not as dorsoventrally robust as in most congeners. The posterolateral corners of female mediosternite VI are broadly rounded and the posterior margin of laterosternite V is roundly sinuate without sharp angles (Fig 16C). The ventral color is light-brown to orangish-brown. The dorsal color is variable, but generally mottled medium- to dark-brown. The parameres of *T*. *humeralis* have been illustrated and shown to be variable among individuals [20 (Fig 22)]; shown here are parameres and other structures dissected from a Montandon-determined male housed at USNM (Fig 16D–16F). Male mediosternite V posterior margin is evenly concave to the laterosternite corners.

### Discussion

Although early reports gave the distribution only as Madagascar [20,35,36], this species has since been determined to be widespread [37] and has been recorded from throughout Madagascar, including from Nossi-Be Island, the type locality [28]. It was reported to also occur on three of the Comoro Islands [37], although we have not seen these specimens and cannot verify the records. This species occurs in the margins of streams, including among vegetation, and has been recorded in puddles and marshes [28]. We have also collected it in residual pools of dry stream beds. Although most specimens are hindwing macropterous, brachypterous individuals occur and have forewing membrane minimized and the claval commissure is longer than the scutellum.

### Published records

Antananarivo [34]; Antsiranana, Fianarantsoa, Mahajanga, Toamasina, Toliara [28].

### Type material examined

LECTOTYPE, ♂: [?]Leucocoris humeralis Sig., Nossi-Bé, Signoret 1880, Heleocoris humeralis Signoret ALM 96, Syntypus? Naucoris humeralis V Signoret 1861, Museo Civico di Genova (MCSN). PARALECTOTYPE, same data as lectotype (1♂ MCSN),

### Additional material examined

Madagascar, **Antananarivo**: Anjozorobe Forest Reserve, small stream next to Saha forest camp, 10 km E of Anjozorobe, 18.4133S, 47.9443E, 1240 m, 23-XI-2014, leg. J. Bergsten, R. Bukontaite, J.H. Randriamihaja, T. Ranarilalatiana, S. Holmgren, MAD14-77 (1♂ NHRS; 1♂ UMC). **Antsiranana**: Nossi-Bé, Museum Paris, Coll. G. Fallou 259–95, Heleocoris humeralis Signt, Montandon det. 1897 (1♂ MNHN); same but with Heleocoris humeralis Signt, Fallou det. (1♂ MNHN); 8 km west of Diego Suarez, Namakia pool in dry river bed, -12.32321S, 49.26441E, 15 m elev, 10-XII-2012, leg. Bergsten Bukontaite, Ranarilalatiana, Randriamihaja, MAD12-58 (5♂, 3♀ NHRS; 3♂, 3♀ UMC); stagnant canal in Antsaba, -13.6459S, 48.73429E, 77 m elev, 21-XI-2012, leg. Bergsten Bukontaite, Ranarilalatiana, Randriamihaja, MAD12-23 (4♂, 3♀ NHRS); Ambakirano River under bridge, 15 km E of Ambilobe along RN 5A, 13.215S, 49.1547E, 50 m, 31-X-2014, sandy river, Bukontaite & Randriamihaja, MAD14-31 (2♂ UMC); 45 km E of Ambilobe along RN 5A, 13.1195S, 13.1195S, 100 m, roadside stream, 31-X-2014, pools in dry stream, Bukontaite & Randriamihaja, MAD14-33 (1♀ NHRS); Andampinifosa village, 45 km N of Sambava on RN5A, Ifosa River crossing road, 14.0594S, 50.0264E, 40 m, 5-XI-2014, small shallow sandy river, leg. Bukontaite & Randriamihaja, MAD14-46 (1♂ UMC); Anjanaribe Sud NP, River Marolakan, 14.7623S, 14.7623S, 920 m, 15-XI-2014, large rocky river, leg. J. Bergsten, R. Bukontaite, J.H. Randriamihaja, T. Ranarilalatiana, S. Holmgren, MAD14-64 (1♀ NHRS); 1 km N of Befingitra village, 4 km from Anjanaharibe-Sud Park NP, 14.6996S, 49.5374E, 680 m, 17-XI-2014, muddy pond, leg. J. Bergsten, R. Bukontaite, J.H. Randriamihaja, T. Ranarilalatiana, S. Holmgren, MAD14-72 (1♂, 4♀ NHRS); Daraina Reserve, Manakulana River, Ankijabe village, 13.2628S, 49.638E, 120 m, 1-XI-2014, pools in dry river, MAD14-34, leg. Bukontaite & Randriamihaja (2♀ NHRS, 1♂ UMC); Daraina Reserve, Antsahabe River, Ankijabe village, 13.2639S, 49.6346E, 130 m, 1-XI-2014, leg. Bukontaite & Randriamihaja, MAD14-35 (3♂, 3♀ UMC); unnamed stream, 14°3.552’S, 50°1.622’E, elev. 65 m, 10-XI-2014, R.W. Sites & K. Miller, rocky stream w/ veg margins, L-1853 (3♂, 2♀ UMC); Lokoho River at Belaoko, 14°34.153’S, 49°44.070’E, elev. 120 m, 11-XI-2014, R.W. Sites & K. Miller, mud, silt, marginal vegetation L-1857 (4♀ UMC); Daraina Reserve, Antsahabe Waterfall, Ankijabe village, 13.276S, 49.6119E, 450 m, 3-XI-2014, leg. Bukontaite & Randriamihaja, MAD14-41 (3♂, 3♀ NHRS; 2♂, 2♀ UMC); river in Mangoaka, 26 km W from Diego Suarez, 12.31326S 49.12532E, 36 m, 10-XII-2012, leg. Bergsten, Bukontaite, Ranarilalatiana, Randriamihaja, MAD12-59 (3♂, 1♀ NHRS); Andranonakoho, 12.92734S, 49.16309E, 154 m, 3-XII-2012, leg. Bergsten, Bukontaite, Ranarilalatiana, Randriamihaja, MAD12-41 (4♂, 3♀ NHRS; 2♂, 2♀ UMC); Sava, Marojejy National Park, 14.4345S, 49.7606E, 710 m, 9-XI-2014, hygropetric rocks and rockpools in wide river, leg. Bergsten, Bukontaite, Ranarilalatiana, Randriamihaja, Holmgren, MAD14-54 (5♂, 4♀ NHRS; 2♂, 2♀ UMC); Nossi-Be: Ambalahonko, marsh next to mangrove,13.4005N, 48.3440E, 27 m, 8-VIII-2019, leg. J. Bergsten & T. Ranarilalatiana, MAD19-10 (1♀ NHRS); Diana, River Mahavanona, 25 km S from Diego Suarez, S12.45538, E49.37619, 70 m, 10-XII-2012, leg. Bergsten & Bukontaite, MAD12-60 (1♀ NHRS). **Fianarantsoa**: Sendrisoa, Ambilavao, N-22°0.585, E46°57.024, 1164 m, 7-V-2006, leg. J. Bergsten et al., hygropetric, standing water with vegetation BMNH (E) 742358 (1♂, 1♀ UMC); N of Ambohimanjaka, Antanavierna River, 20°10.260’S, 47°5.437’E, elev. 1355 m, 1-XI-2014, R.W. Sites, sandy bottom, irrigation channel, L-1828 (1♂ UMC); Ranomafana National Park, Namorona River, 21°15.494’S, 47°25.284’E, elev. 943 m, 3-XI-2014, R.W. Sites, mossy rocks of waterfall, L-1837 (1♂ UMC); Ranomafana National Park, Amboditanimena River at Amboditanimena, nr. confluence with Namorona River, 21°13.584’S, 47°22.182’E, elev. 952 m, 3-XI-2014, R.W. Sites, small sandy stream w leafpacks & dense canopy, L-1841 (1♂ UMC); Ranomafana NP, Research House, 26-XI-1994, 21°15’38"S, 47°25’11"E, M.A. Ivie & D.A. Pollock (1♂, 1♀ UMC); same but 925 m, bank of Namorona river (2♀ UMC); Ranomafana National Park, Namorona River at Namorona Village, 21°15.738 S, 47°27.264’ E, elev. 621 m, 2-XI-2014, R.W. Sites, silty with cobble & marginal grasses, L-1830 (1♀ UMC); Ihorombe, Maropaika commune, 2 km from Maropaika, on way to Vondrozo, S22.734166, E46.9887, 581 m, 12-XII-2013, lake in open area, leg. Randriamihaja & Ranarilalatiana, MAD13-65 (1♂ NHRS). **Mahajanga**: Maropapango, River Maropapango under bridge RN6, -14.35419S, 48.01984E, 13 m, 18-XI-2012, leg. Bergsten, Bukontaite, Ranarilalatiana, Randriamihaja, MAD12-10 (1♂, 1♀ NHRS; 1♂, 1♀ UMC); Boeny, Mahavavy Kinkony RS, S16.15890, E045.93967, 11 m.a.o., 3-XII-2009, leg. J. Bergsten, N. Jönsson, T. Ranarilalatiana, H.J. Randriamihaja, MAD09-21 (1♂, 1♀ NHRS). **Toamasina**: Alaotra-Mangoro, Zahamena NP, Manambato River by Camp Bemoara, 17.5126S, 48.7267E, 1050 m, 6-III-2018, rockpools, leg. J. Bergsten & T. Ranarilalatiana, MAD18-77 (1♂, 1♀ NHRS); Alaotra-Mangoro Zahamena Nat. Pk., Antanandava Sect., Manambato River by Camp Bemoara, rockpools, mid elev. rainforest, 17.5126S, 48.7267E, 1050m, 6-III-2018, leg. J. Bergsten & T. Ranarilalatiana, MAD18-77 (1♂ UMC, DNA extracted—1♀ UMC). Centmetre, 18°55.626’S, 48°25.219’E, elev. 935 m, 15-XI-2014, R.W. Sites, pond with grasses, L-1869 (2♀ UMC). **Toliara**: Tulear, Nat. Rd. 13, 321 m, desert, Ambovombe, Andalatanosy, 22°44’10"S, 45°35’02"E, dry stream bed w/pond (1♂ UMC); Tulear, 3.5 km N Betroka, 758 m, 23°14’00"S, 46°05’07"E, 20-XI-1994, pool in wash, M.A. Ivie & D.A. Pollock (1♂, 1♀ UMC). **Unknown Province:** Collection Le Moult, Juin, J.R. de la Torre-Bueno Collection K.U., Exchange KU-1973, H. humeralis Sign., Heleocoris humeralis Sign. det Montandon 1911, Heleocoris humeralis Sign. det J.T. Polhemus (1♂ USNM); Museum Paris, Coll. G. Fallou 259–95, Heleocoris humeralis Signt, Montandon det. 1897 (1♂ MNHN); same but with Exchange-Paris 2017 (1♀ UMC).

## *Tsingala latiforma* NEW SPECIES

urn:lsid:zoobank.org:act:D74EBE60-E444-43D0-ADF7-EF956DD36D12

(Fig 17)

### Macropterous male

**Fig 17:**
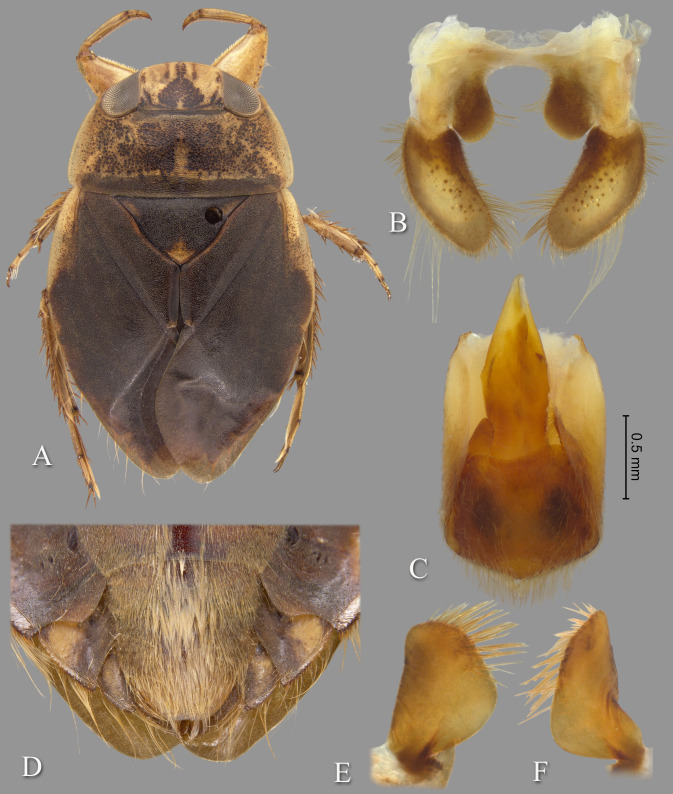
Tsingala latiforma n.sp. (A) Holotype macropterous male, (B) male 8^th^ abdominal tergum, (C) male genital capsule with proctiger and tergum IX removed, (D) female terminal abdominal sterna with subgenital plate, (E) left paramere, (F) right paramere. Size bar pertains only to C.

Holotype, length 9.68; maximum width across embolia 6.04. Paratypes (n = 5), length 9.68–10.16 (mean = 9.95); maximum width 6.00–6.24 (mean = 6.14). Overall shape ovate. Dorsally, overall coloration dark-brown with yellowish head, pronotum, proximal 2/3 of embolium (Fig 17A); brown, coarse punctation on head and pronotum; profuse yellow, fine punctation on hemelytra. Ventrally, mostly medium-brown, yellowish legs and propleuron.

*Head*. Length 1.24, maximum width 4.04; inner margin of eyes slightly convergent anteriorly. Synthlipsis at anterior margin 1.84, interocular distance at posteromesal corner 2.00; cuticle laterad of eye expanded. Vertex with heavy, brown punctation forming broad triangle interrupted by yellow posteromedian gap; punctation extending anteriorly as irregular, fused, double row of coarse punctation, flanked by lighter, paramedian row of punctation; irregular row of setae marked by broken line of brown pigmentation bordering inner margin of eyes; brown line of pigmentation extending from near posteromedian corner of eye to posteromedian margin of head. Anterior margin broadly rounded dorsoventrally. Labrum with basal sulcus, transverse, distal margin evenly rounded, 2.32× wider than long. Antennae four-segmented, length 0.88, proportions 3, 16, 20, 9.

*Thorax*. Pronotum yellow, scabrous, broad, 2.5× wider than long, length at midline 2.04; maximum width at posterolateral corners 5.12; transverse darker band across posterior 1/4; large, brown punctures tending to coalesce near midline, becoming smaller and more sparse laterally, with distinct yellow impunctate subrectangular area on midline anterior to transverse band; lateral margin evenly convex, posterolateral corners rounded, posterior margin shallowly convex, anterior margin shallowly concave; ventrally, propleuron generally medium-brown except yellow lateral glabrous area. Scutellum finely punctate, triangular, dark-brown with yellow apically and along lateral margins near anterior corners, with distinctly sinuate lateral margins, 1.90× wider than long, width 3.04, length 1.60. Hemelytra finely punctate throughout; with sparse, short, golden setae on corium especially evident laterally, setae extending only to base of membrane. Claval commissure length (to locking mechanism) 1.36. Embolium length 3.32 (chord measurement), greatest width 0.84; lateral margin evenly rounded; yellowish with scattered brown punctures in anterior 2/3, brown in posterior third with dense white punctures. Hindwing extending to near posterior margin of tergum V. Metaxyphus broad, with sharp mid-ventral ridge. Coxae brown, all other leg segments yellow. Profemur elongate, inflated, anterior margin with dorsal and ventral rows of golden hairs sandwiching one row of short, dark spines. Distal 2/3 of protibia and two-segmented protarsus with dense ventral pad of setae. Propretarsus with short, stout, paired, movable claws. Meso- and metafemora with anteroventral and mid- or posteroventral rows of spinose setae; anteroventral seta rows arcuate from ventral surface to anterior margin, ending in distal fourth; setae number 25–30 on mesofemur and 30–33 on metafemur; mesofemur midventral row with 65–70 setae, with the distalmost 14–15 setae spaced distinctly more tightly; metafemur posteroventral row with 45–48 stout setae. Mesotrochanter and mesofemur with profuse brush of light colored setae on posteroventral margin; brush of setae narrowly set on metatrochanter and metafemur. Thick pad of setae on mesotibia and mesotarsomeres 2 and 3. Long golden swimming hairs sparse on mesotibia and tarsus, profuse on metatibia and tarsus. Middle and hind legs with tarsomere 1 extending beneath tarsomere 2. Pretarsal claws long and with slight, even curvature. Leg measurements as follows: foreleg, femur 2.44, tibia 1.54, tarsomeres 1–2 0.24, 0.28; middle leg, femur 2.38, tibia 1.80, tarsomeres 1–3 0.20, 0.46, 0.52; hind leg, femur 2.82, tibia 2.80, tarsomeres 1–3 0.22, 0.96, 0.80.

*Abdomen*. Dorsally with lateral margins of II–VIII with regular row of short, stout, golden-brown spines. Terga with lateral margins of II–VII each yellow anteriorly, dark brown at posterolateral corner. Ventrally dark-brown with dense mid-ventral band of elongate setae beginning on III, continuing to posterior margin of abdomen. Posterior margins of mediosternite III and IV straight, V with slight asymmetry and deeply concave, VI asymmetrical, shallowly convex, more expansive on left side, VII symmetrical and shallowly convex. Aedeagus elongate, stout, widest at middle of part visible beyond pygophore, left lateral margin in distal half straight and angled toward apex, right lateral margin gently convex throughout most of length until apex, apex slightly deflected to right (Fig 17C). Pygophore with anterior margin concave between parameres, slight asymmetry in anterior production of lateral lobes, brush of long setae most prominent posteriorly (Fig 17C). Parameres dramatically asymmetrical; left paramere broad, with left margin straight for most of length, posterior margin shallowly convex, mesal margin straight, both corners broadly rounded, elongate setae extending mesad from distal half of near mesal margin (Fig 17E); right paramere with lateral margin sharply angled, posterior margin straight, mesal margin shallowly convex, posteromesal corner broadly rounded, anterior corner produced to a blunt point, elongate setae extending mesad from distal 2/3 of mesal margin (Fig 17F).

### Macropterous female

Paratypes (n = 2), length 8.96–9.84 (mean = 9.40); maximum width 7.56–8.21 (mean = 7.88). Similar to male in general structure and coloration with following exceptions: Protarsus one-segmented; pads of setae greatly reduced on pro- and mesotibiae and tarsi. Posterior margins of mediosternites III–VI symmetrical and nearly straight. Posterior margin of laterosternite V with mesal half lobed posteriorly, inflection point near middle of margin broadly or narrowly rounded. Posterolateral corners of mediosternite VI rounded. Posterior margin of subgenital plate (mediosternite VII) broadly rounded (some specimens with straightened posterolateral margins) and with slight medial concavity, 1.37× wider than long, width 1.32, length 1.04 (Fig 17D).

### Submacropterous form

Unknown.

### Diagnosis

This species can be characterized only by its unique combination of broadly expansive male parameres, especially the left paramere, and broadly rounded female subgenital plate with the posterolateral corners of mediosternite VI broadly rounded.

### Discussion

This species is known only from standing water bodies in three localities in Fianarantsoa Province; two are ponds and one was from pools in a drawn-down stream. Other undescribed species also have broad parameres, but mediosternite VI posterolateral corners have small productions and in some the subgenital plates have convergent lateral margins with a truncated apex. Further, these other populations are of dramatically different body sizes; thus, this group requires further study.

### Etymology

The specific epithet *lati-* (= wide, broad) and *form* (= shape) from Latin is in reference to both the unusually broad male parameres and female subgenital plate.

### Type material examined

HOLOTYPE ♂: Madagascar, **Fianarantsoa**: District Ambositra, S of Ambalamakana, 20°27.381’S, 47°13.855’E, elev. 1328 m, 1-XI-2014, R.W. Sites, pond with lily pads and marginal veg, L-1829 (UMC). PARATYPES: same data as holotype (1♂, 1♀ UMC); Ambositra, 4.8 km N Ambatofitorahana, 20°46’22.6"S, 47°10’48.7"E, 1708 m, 1-XI-2014, leg. G. Gustafson & K.B. Miller, pond off road, GTG110114B (2♂, 1♀ UMC); N of Ambohimanjaka, 20°14.019’S, 47°5.611’E, elev. 1461 m, 5-XI-2014, R.W. Sites, cascade w/ pools, marginal grasses, L-1845 (2♂, 3♀ UMC). Antsiranana: Ambilomagodra, S-13.0078, E49.13313, 139 m, 30-XI-2012, colls: Bergsten, Bukontaite, Ranarilalatiana, & Randriamihaja, MAD12-33 (DNA extracted—1♀ UMC; 1♂, 2♀ NHRS).

## *Tsingala naucoroides* (Montandon, 1897)

(Fig 18)

*Heleocoris naucoroides* Montandon 1897a: Ann. Soc. Ent. Belg. 41: 57–58.

*Heleocoris naucoroides*: Montandon 1897b, Verh. K. K. Zool. Bot. Gesell. Wien 15: 445 (key).

*Heleocoris naucoroides*: Poisson 1951, Mem. Inst. Sci. Madag. 5: 100–101.

*Heleocoris naucoroides*: Poisson 1962a, Bull. Soc. Sci. Bret. 37: 34–39 (key).

*Heleocoris naucoroides ambiguus* Poisson 1962a: Bull. Soc. Sci. Bret. 37: 34, 37–39 (description).

*Heleocoris naucoroides*: La Rivers 1974, Biol. Soc. Nev. Occas. Pap. 38: 12 (catalog).

*Tsingala naucoroides*: Sites 2022, Zool. J. Linn. Soc. 195: 1273.

**Fig 18:**
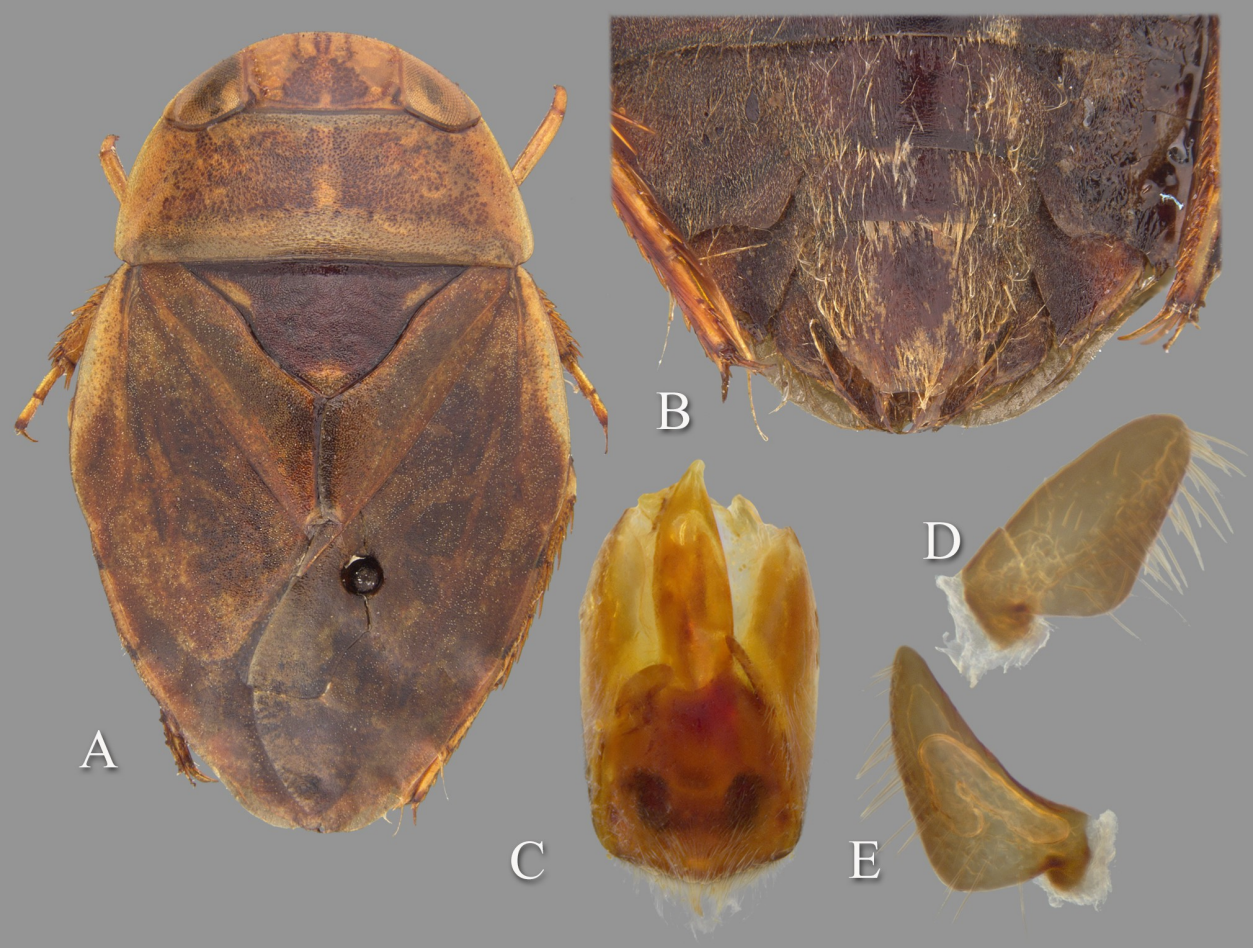
Tsingala naucoroides. (A) Lectotype macropterous female, (B) lectotype terminal abdominal sterna with subgenital plate, (C–E) genitalia of Montandon-determined male (MNHN), (C) genital capsule with proctiger and tergum IX removed, (D) left paramere, (E) right paramere.

### Lectotype designation

In the original description, Montandon [38] indicated specimens were deposited in Genoa (MCSN), Vienna, and his personal collection. Currently, a single female syntype is housed at MCSN, three are in Vienna (one male and two females), and two in NHMUK (one male and one female). We examined the syntype female on loan from MCSN and a pair Montandon-determined specimens from MNHN. The syntypes deposited at NHMUK and NHMW were not examined. In order to fix the identity of this species, we herein designate the female deposited at MCSN as the lectotype (Fig 18A and 18B). The specimens at NHMUK and NHMW are paralectotypes.

### Diagnosis

The large size and dark coloration of this species provide the first indication of its identity. This species is among the largest in the genus; it was reported as 9.5–9.8 mm in length in the original description [38]. Our specimens measure 9.12–9.92 in length. The female subgenital plate is broad and its lateral margins are convergent and nearly straight from laterosternite VI to a truncate or shallowly concave apex. The posterolateral corners of mediosternite VI are variable, ranging from broadly to narrowly rounded or with an incipient production. The posterior margin of laterosternite V in females is roundly sinuate and usually without sharp angles, although an incipient angle occasionally occurs at the inflection point. The ventral color of the abdomen is dark-brown to black. The dorsal color can be slightly lighter, with the hemelytra generally concolorous brown to dark-brown.

### Discussion

This species is found throughout the country. We have collected it in a variety of aquatic habitats, including stream margins, ponds with lily pads, rock pools, and even pools on a road. The subspecies *H*. *n*. *ambiguus* was differentiated from the nominate form by a smaller and more slender size and shape, and by parameres with substantial differences [37]. When considering all species of *Tsingala* from across Madagascar, this taxon would seem to warrant species-level recognition. However, in the absence of a type or authoritatively identified specimen, the two-sentence description and illustration of only parameres renders this subspecies as a *nomen dubium*.

**Fig 19:**
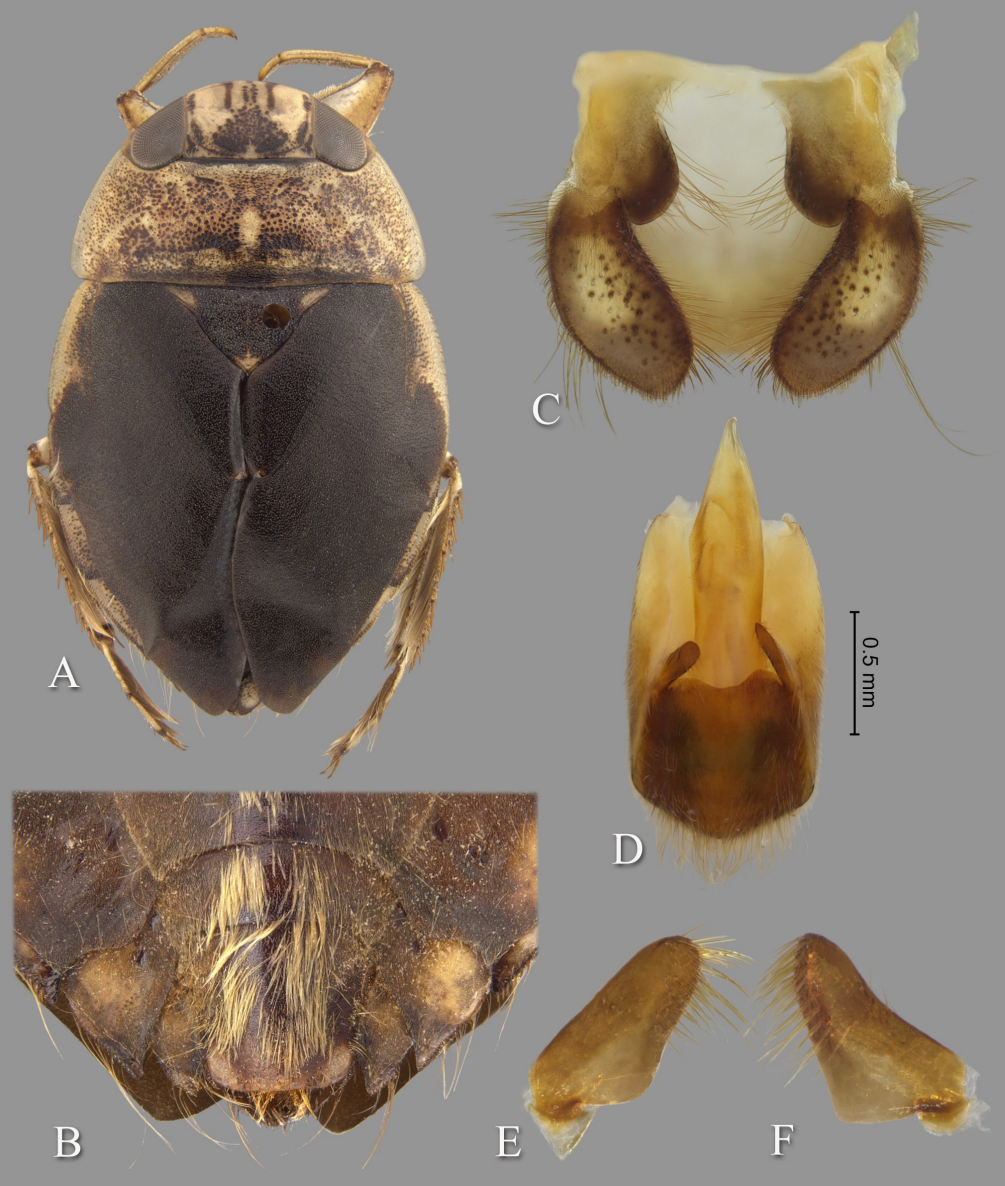
Tsingala spatulata n.sp. (A) Holotype submacropterous female, (B) female terminal abdominal sterna with subgenital plate, (C) male 8^th^ abdominal tergum, (D) male genital capsule with proctiger and tergum IX removed, (E) left paramere, (F) right paramere. Size bar pertains only to D.

**Fig 20:**
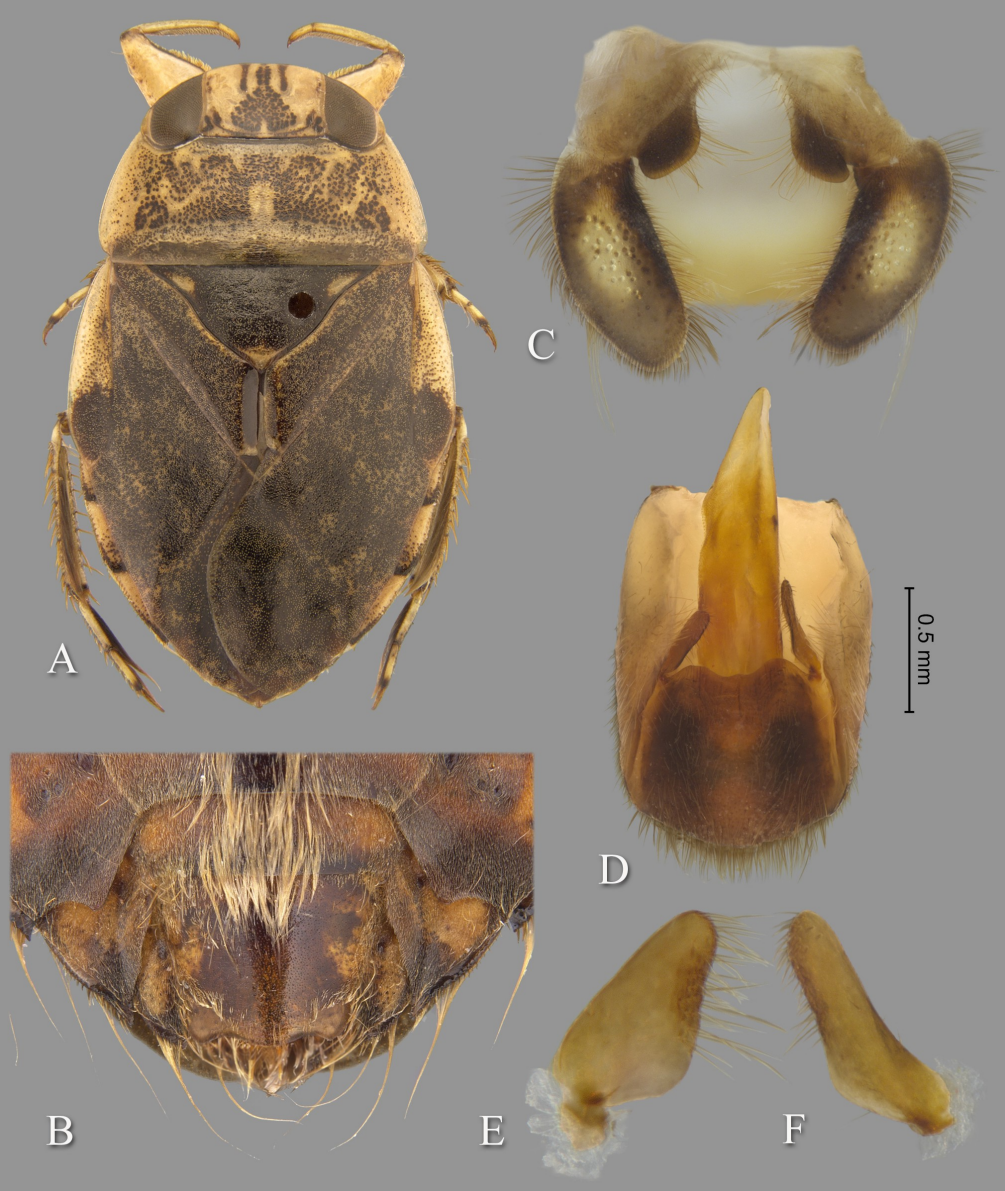
Tsingala trilobata n.sp. (A) Holotype macropterous female, (B) female terminal abdominal sterna with subgenital plate, (C) male 8^th^ abdominal tergum, (D) male genital capsule with proctiger and tergum IX removed, (E) left paramere, (F) right paramere. Size bar pertains only to Fig D.

**Fig 21:**
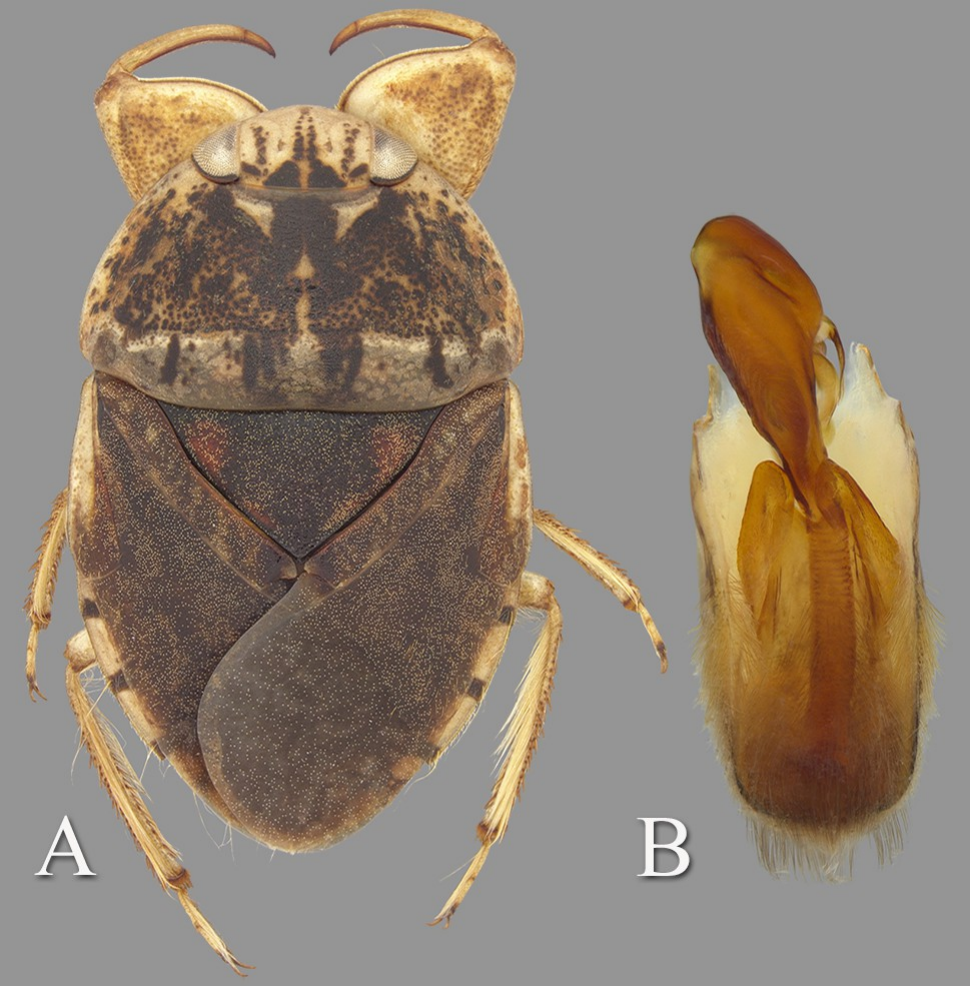
Macrocoris distinctus. (A) Macropterous male, (B) male genital capsule with proctiger and tergum IX removed.

Line drawings of abdominal terga 6–8, the aedeagus, and parameres of *T*. *naucoroides* were presented by Poisson [26 (Figs 19–21)], and parameres from two populations showing intraspecific variation by Poisson [37, Fig 3)].

### Published records

*T*. *n*. *naucoroides*: Antananarivo [38], Fianarantsoa [26], Toamasina, Toliara [37]. *T*. *naucoroides ambiguus*: Antananarivo, Antsiranana [37].

### Type material examined

LECTOTYPE, ♀ of *T*. *n*. *naucoroides*: Madagas. Pipitz 83, Typus, Heleocoris naucoroides Montand. type, Syntypus Heleocoris naucoroides A.-L. Montandon 1897, Museo Civico di Genova (MCSN).

### Additional material examined

Madagascar, **Antananarivo**: Andranofena River at Andranofena Sud village, 18.0844S, 47.1776E, 1430 m, 21-XI-2014, leg. Bergsten, Bukontaite, Randriamihaja, Ranarilalatiana, Holmgren, MAD14-74 (1♂, 1♀ NHRS; 1♂ UMC); Museum Paris, Madagascar Tananarive, R. Decary 1921 / 28369 det. 19 H.B. Hungerford / Europ. Trip 1928 / Heleocoris naucoroides Mont. det Hungerford (1♀ SEMC). **Antsiranana**: Sava, Marojejy NP, stream over open bedrock, 14.4339S, 49.7600E, 720m, 6-II-2018, leg. J. Bergsten & T. Ranarilalatiana, MAD18-11 (2♂, 1♀ NHRS; DNA extracted—1♀ UMC); River Antsabalahy by Maholera village, -14.03715S, 48.22945E, 21-XI-2012, leg. Bergsten, Bukontaite, Ranarilalatiana, Randriamihaja, MAD12-30 (1♂, 1♀ NHRS; 1♀ UMC); west entrance to Ankarana NP, -12.93103S, 49.05585E, 38 m, 30-XI-2012, blacklight trap, leg. Bergsten, Bukontaite, Ranarilalatiana, Randriamihaja, MAD12-34 (1♂ NHRS). **Fianarantsoa**: Canyon de Rats, -22.48019S, 45.37805E, 751 m, 13-XI-2012, leg. Bergsten, Bukontaite, Ranarilalatiana, Randriamihaja, MAD12-04 (1♀ NHRS); Sava, Marojejy National Park, 14.4345S, 49.7606E, 710 m, 9-XI-2014, hygropetric rocks and rockpools in wide river, leg. Bergsten, Bukontaite, Ranarilalatiana, Randriamihaja, Holmgren, MAD14-54 (6♂, 3♀ NHRS; 5♂, 2♀ UMC); Ihorombe, R.S. Pic d’Ivohibe, Marovitsika, Marovitsika stream, S22.475683, E46.95076, 974 m, 8-XII-2013, stream with pools, leg. J.H. Randriamihaja & T. Ranarilalatiana, MAD13-47 (1♂, 1♀ NHRS); Ihorombe, Mahatsinjotsifoka village, 2 km from Mahatsinjotsifoka to Ivohibe, S22.517783, E46.6739, 761 m, 7-XII-2013, pools on the road, leg. J.H. Randriamihaja & T. Ranarilalatiana, MAD13-45 (1♀ NHRS); District Ambositra, S of Ambalamakana, 20°27.381’S, 47°13.855’E, elev. 1328 m, 1-XI-2014, R.W. Sites, pond with lily pads and marginal veg, L-1829 (1♂, 5♀ UMC); Ranomafana National Park, Tomaro River at Ambatolahy, 21°15.024’S, 47°25.756’E, elev. 872 m, 3-XI-2014, R. W. Sites, rocky with vegetated margins, L-1842 (1♂, 1♀ UMC); Isalo Park, entrance of de Makis canyon, -22.48684S, 45.37668E, 706 m, 12-XI-2012, leg. Bergsten, Bukontaite, Ranarilalatiana, Randriamihaja, MAD12-01 (1♀ NHRS); N of Ambohimanjaka, Antanavierna River, 20°10.260’S, 47°5.437’E, elev. 1355 m, 5-XI-2014, R.W. Sites, shallow, sandy w/ veg. margins, L-1846 (1♀ UMC). **Mahajanga**: Melaky, btw. Morafenobe-Ambohijanahary, S18.20675, E045.31783, 711 m, 19-XII-2009, leg. J. Bergsten, N. Jönsson, T. Ranarilalatiana, H.J. Randriamihaja, MAD9-75 (1♀). **Toamasina**: 22 km E. Manjakantriana, 18 55.22S, 47 45.13’E, 12-XI-1994, riparian, M.A. Ivie & D.A. Pollock (1♀ UMC); Andasibe National Park, tributary ca. 200 m E of Lac Vert, 18°56’18.12"S, 48°25’18.72"E, elev. 949 m, 13-XI-2014, R.W. Sites, L-1861 (1♀ UMC); Antongil B., Mocquerys / Exchange from Budapest Mus., H.B. Hungerford / H. naucoroides, det. G. Horváth de Mont. (1♀ SEMC). **Toliara**: N. of Fort Dauphin [Tolanaro], 16-XI-1994, 24°47’S, 46°52’E, 46 m, leg. M.A. Ivie & D.A. Pollock (1♀ UMC).

## *Tsingala nossibeanus* (Bergroth, 1893)

*Heleocoris nossibeanus* Bergroth, 1893: Rev. Entom. 12: 212–213.

*Heleocoris nossibeanus*: Montandon 1897b, Verh. K. K. Zool. Bot. Gesell. Wien 15: 447 (key).

*Heleocoris nossibeanus*: Poisson 1962a, Bull. Soc. Sci. Bret. 37: 36, 39–40.

*Heleocoris nossibeanus*: La Rivers 1974, Biol. Soc. Nev. Mem. 38: 12 (catalog).

*Tsingala nossibeanus*: Sites 2022, Zool. J. Linn. Soc. 195: 1273.

### Diagnosis

The inner margins of the compound eyes are subparallel in the posterior half and noticeably convergent in the anterior half [37]. Body length is ≤ 8.5 mm. The parameres are dramatically asymmetrical and with shapes as in Fig 6 of Poisson [37].

### Discussion

This species is known only from Antsiranana Province. The type locality is the island of Nossi-Bé and the species has also been reported from Sambirano River [37]. The type specimen was reported by Bergroth [39] to be deposited in the museum at Frankfurt am Main. Montandon [25] reported that he was not familiar with the species; thus, he had not seen the type, and Poisson [37] later reported the type to be missing. In the absence of a type specimen or any authoritatively identified museum specimens, we rely on the original description [39], which gave color characteristics, although generally these are not reliable species descriptors in *Tsingala*, and that the eyes are convergent anteriorly. Secondarily, we rely on the species concepts of Montandon [25] and Poisson [37], who redescribed the species and provided line drawings of male parameres from two populations showing intraspecific variation [37 (Fig 6)]. It is unknown upon what material Poisson based his redescription, although he gave the locality as Nosi-Be. Poisson repeated the size range given in the original description [39] as 7.8–8.5 mm and that that eyes are noticeably convergent. We have morphospecies with parameres resembling those illustrated by [37], and with female subgenital plates of various shapes; however, all of our specimens have compound eyes that are parallel or barely, rather than noticeably, convergent and some are substantially longer than 8.5 mm; thus, they apparently are not conspecific with *T*. *nossibeanus*. As such, we consider these morphospecies to represent new species and described one as *T*. *latiforma* **n.sp.**

### Published records

Antsiranana [37,39].

### Material examined

None.

## *Tsingala spatulata* NEW SPECIES

urn:lsid:zoobank.org:act:D115ACE1-DE87-42F3-9860-9F8B09BECD93

(Fig 19)

### Submacropterous female

Holotype, length 9.84; maximum width across embolia 5.88. Paratypes (n = 10), length 8.64–10.40 (mean = 9.47); maximum width 5.20–6.00 (mean = 5.71). Overall shape elongate-oval. Dorsally, overall coloration dark-brown with yellowish head, pronotum, proximal half of embolium (Fig 19A); brown, coarse punctation on head and pronotum; hemelytra dark-brown and profusely punctate. Ventrally, abdomen light-brown, thorax yellow and medium-brown, legs yellow.

*Head*. length 1.24, maximum width 3.68 inner margin of eyes slightly convergent anteriorly. Synthlipsis at anterior margin 1.80, interocular distance at posteromesal corner 1.92; cuticle laterad of eye expanded. Vertex with heavy, brown punctation forming broad triangle interrupted by yellow posteromedian gap; punctation extending anteriorly as irregular, double row of coarse punctation, flanked by dark, paramedian row of coalescent punctation; irregular row of setae marked by broken line of brown pigmentation bordering inner margin of eyes; dark-brown line of pigmentation extending from near posteromedian corner of eye to posteromedian margin of head. Anterior margin broadly rounded dorsoventrally. Labrum with basal sulcus, transverse, distal margin evenly rounded, 2.17× wider than long. Antennae four-segmented, length 0.72, proportions 1, 3, 4, 2.

*Thorax*. Pronotum yellow, scabrous, broad, 2.70× wider than long, length at midline 1.84; maximum width at posterolateral corners 4.96; transverse band across posterior 1/4 devoid of coarse punctures; large, brown punctures tending to coalesce near posterior midline, diminishing in size near lateral margins, with distinct yellow impunctate subrectangular area on midline anterior to transverse band; lateral margin evenly convex, posterolateral corners rounded, posterior margin very shallowly convex, anterior margin straight; ventrally, propleuron generally yellow, with light-brown near posterior margin and dark-brown semicircle at lateral margin of anterior pruinose lobe. Scutellum finely punctate, triangular, black with yellow apically and along lateral margins near anterior corners, with distinctly sinuate lateral margins, 2.22× wider than long, width 2.84, length 1.28. Hemelytra continuously dark-brown to black; finely punctate throughout except embolium; with sparse, short, golden setae. Embolium length 3.00, greatest width 0.80; lateral margin evenly rounded; yellow with scattered brown punctures in anterior half, brown in posterior half. Claval and intraclaval sutures present, but suppressed; claval commissure length (to locking mechanism) 1.48. Membrane lacking on right hemelytron, reduced to narrow strip on left hemelytron that subducts beneath right. Hindwing extending to posterior half of tergum IV. Metaxyphus broad, with sharp mid-ventral ridge and papillose apex. All leg segments pale yellow, coxae slightly darker. Profemur elongate, inflated, anterior margin with dorsal and ventral rows of golden hairs sandwiching one row of short, dark spines. Distal 3/4 of protibia and single-segmented protarsus with dense ventral pad of setae. Propretarsus with short, stout, paired, movable claws. Meso- and metafemora with anteroventral and mid- or posteroventral rows of spinose setae; anteroventral seta rows arcuate from ventral surface to anterior margin, ending in distal fourth; setae number 30–32 on mesofemur and 33–34 on metafemur; mesofemur midventral row with 51–57 setae, with the distalmost 10–16 setae spaced distinctly more tightly; metafemur posteroventral row with 48–50 stout setae. Mesotrochanter and mesofemur with profuse brush of light colored setae on posteroventral margin; brush of setae narrowly set on metatrochanter and metafemur. Elongate, narrow pad of setae on mesotibia and mesotarsomeres 2 and 3. Long golden swimming hairs sparse on mesotibia and tarsus, profuse on metatibia and tarsus. Middle and hind legs with tarsomere 1 extending beneath tarsomere 2. Pretarsal claws long and with slight, even curvature. Leg measurements as follows: foreleg, femur 2.30, tibia 1.60, tarsus 0.44; middle leg, femur 2.26, tibia 1.62, tarsomeres 1–3 0.18, 0.42, 0.44; hind leg, femur 2.76, tibia 2.80, tarsomeres 1–3 0.26, 0.94, 0.72.

*Abdomen*. Dorsally with lateral margins of II–VIII with regular row of short, stout, golden-brown spines. Terga with lateral margins of II–VII each yellow anteriorly, dark brown at posterolateral corner. Ventrally with dense mid-ventral band of elongate setae beginning on IV, continuing and becoming more profuse to and including subgenital plate. Posterior margins of mediosternites III–VI symmetrical and nearly straight. Posterior margin of laterosternite V sinuate, roundly convex in medial half, concave in distal half. Posterolateral corners of mediosternite VI produced posteriorly as blunt tabs, which are partially concealed by vestiture. Subgenital plate (mediosternite VII) lateral margins slightly convergent to broadly truncate posterior margin, posterolateral corners rounded, 1.16× wider than long, width 1.18, length 1.02 (Fig 19B).

### Submacropterous male

Paratypes (n = 10), length 8.40–9.20 (mean = 8.73); maximum width 5.20–5.76 (mean = 5.45). Similar to female in general structure and coloration with following exceptions: Protarsus two-segmented. Pads of setae more pronounced on pro- and mesotibiae and tarsi. Posterior margins of mediosternite symmetrical, III–IV straight, V deeply concave, VI–VII straight to shallowly convex. Tergum VIII with pseudoparameres symmetrical and with posterior margin shallowly rounded and oriented posteromesad (Fig 19C). Aedeagus elongate, stout, widest at distal third, left lateral margin in distal third straight and angled toward apex, right lateral margin gently convex until near apex, apex slightly deflected to right (Fig 19D). Pygophore with anterior margin concave between parameres, slight asymmetry in anterior production of lateral lobes, brush of long setae most prominent posteriorly (Fig 19D). Parameres asymmetrical; left paramere with margins generally straight, posteromesal, both corners broadly rounded, elongate setae extending mesad from anterior 2/3 of mesal margin and anterior corner (Fig 19E); right paramere with lateral margin subtly angled creating broadly concave appearance, posterior and mesal margins straight, posteromesal corner narrowly rounded and right angled, anterior corner bluntly rounded, elongate setae extending mesad from anterior 2/3 of mesal margin (Fig 19F).

### Macropterous form

Unknown.

### Diagnosis

This species is known from only the submacropterous form; thus, the lack of membrane of the right hemelytron and nearly uniformly dark-brown to black hemelytra (except embolium) are diagnostic for this species of *Tsingala*. The claval commissure is longer than the length of the scutellum. Males have no other diagnostic features; however, females have a uniquely shaped subgenital plate in that the posterior margin is broadly truncate with rounded corners (Fig 19B).

### Discussion

We collected this species in both lentic and lotic habitats. More specifically, in standing water we found it among vegetation and in rockpools, and in flowing water among marginal vegetation. In the recent phylogeny of the family [21], this species was given as *Tsingala* sp. E and was sister to an undetermined species of *Tsingala* close to *T*. *latiforma*.

### Etymology

The specific epithet derives from the New Latin adjective *spatulata* (= spatula-like) and is in reference to the shape of the female subgenital plate.

### Type material examined

HOLOTYPE ♀: Madagascar: **Antsiranana:** Marojejy National Park, Humbert Waterfall, 14°25.970’S, 49°46.388’E, elev. 519 m, 8-XI-2014, rock pools, R.W. Sites, L-1849 (UMC). PARATYPES: **Antsiranana:** same data as holotype (6♂, 7♀ UMC); Sava, Marojejy NP, Humbert Waterfall, 14.4333S, 49.773E, 550 m, 8–12-XI-2014, leg. J. Bergsten, R. Bukontaite, J.H. Randriamihaja, T. Ranarilalatiana, S. Holmgren, MAD14-48 (7♂, 4♀ NHRS; 7♂, 4♀ UMC); Sava, Marojejy National Park, 14.4369S, 49.7749E, 490 m, waterhole with dead leaves next to stream, 8-XI-2014, leg. Bergsten, Bukontaite, Ranarilalatiana, Randriamihaja, Holmgren, MAD14-50 (1♂ NHRS); Sava, Marojejy National Park, 14.4354S, 49.768E, 590 m, forest stream with pools, stones, dead leaves, 9-XI-2014, leg. Bergsten, Bukontaite, Ranarilalatiana, Randriamihaja, Holmgren, MAD14-53 (1♂ NHRS); Sava, Marojejy National Park, 14.4345S, 49.7606E, 710 m, 9-XI-2014, hygropetric rocks and rockpools in wide river, leg. Bergsten, Bukontaite, Ranarilalatiana, Randriamihaja, Holmgren, MAD14-54 (1♀ NHRS); Sava, Marojejy National Park, 14.4329S, 49.7592E, 640 m, rocky forest stream, 10-XI -2014, leg. Bergsten, Bukontaite, Ranarilalatiana, Randriamihaja, Holmgren, MAD14-57 (1♂, 1♀ NHRS); Sava, Marojejy National Park, 14.4354S, 49.768E, 590 m, forest stream with pools, stones, dead leaves, 11-XI-2014, leg. Bergsten, Bukontaite, Ranarilalatiana, Randriamihaja, Holmgren, MAD14-59 (2♂ NHRS); Sava, Marojejy NP, Ambinanitelo River, 14.4370S, 49.7751E, 470 m, 5-II-2018, leg. J. Bergsten & T. Ranarilalatiana, MAD18-03 (4♂, 1♀ NHRS; 3♂ UMC); Sava, Marojejy NP, stream and streampool, 14.4339S, 49.7600E, 721 m, 6-II-2018, leg. J. Bergsten & T. Ranarilalatiana, MAD18-12 (1♂, 1♀ NHRS); Marojejy National Park, 14°26.228’S, 49°46.549’E, elev. 459 m, 8-XI-2014, R.W. Sites, forest stream w/ marginal veg., L-1848 (1♂ UMC); Befosa River, 2 km E of Beraty, 14.02944S, 48.27491E, 328 m, 22-XI-2012, leg. Bergsten, Bukontaite, Ranarilalatiana, Randriamihaja, MAD12-22 (1♂ NHRS); 12.52859S, 49.16982E, Montagne d’Ambre NP, above Cascade Sacre, 6-XII-2012, leg. Bergsten, Bukontaite, Ranarilalatiana, Randriamihaja, MAD12-50 (1♂, 1♀ NHRS; 1♂, 1♀ UMC); 12.52456S, 49.17255E, 1037 m, Montagne d’Ambre NP, downstream from Cascade Sacre, 5-XII-2012, leg. Bergsten, Bukontaite, Ranarilalatiana, Randriamihaja, MAD12-48 (2♂, 1♀ NHRS; 2♂, 2♀ UMC); Daraina Reserve, Antsahabe Waterfall, Ankijabe village, 13.276S, 49.6119E, 450 m, 3-XI-2014, leg. Bukontaite & Randriamihaja, MAD14-41 (3♂, 1♀ NHRS); Anjanaribe Sud NP, River Marolakan, 14.7623S, 14.7623S, 920 m, 15-XI-2014, large rocky river, leg. J. Bergsten, R. Bukontaite, J.H. Randriamihaja, T. Ranarilalatiana, S. Holmgren, MAD14-64 (1♂ NHRS). **Fianarantsoa:** Ranomafana National Park, unnamed stream nr confl. w/Namorona Riv., 21°14.425’S, 47°23.649’E, elev. 1135 m, 2-XI-2014, R.W. Sites, marginal veg, open canopy, L-1831 (1♀ UMC); Ranomafana National Park, Matsiara Ambony, 21.2395S, 47.3947E, 1130 m, forest stream with sandy bottom, 2-XI-2014, Bergsten, Ranarilalatiana, Holmgren, MAD14-04 (1♀ NHRS); 21°25.030’S, 47°42.900’E, 4-XI-2014, K.B. Miller (1♂, 1♀ UMC); Sendrisoa, Ambilavao, N-22°0.585, E46°57.024, 1165 m, 7-V-2006, standing water with vegetation, leg. Bergsten et al, BMNH(E)742186 (1♂, 2♀ NHRS). **Toamasina**: Alaotra-Mangoro, Zahamena NP, Antanandava Sect., Manambato River by Camp Cascade, 17.5450S, 48.7237E, 1290 m, 9-III-2018, leg. J. Bergsten & T. Ranarilalatiana, MAD18-100 (1♀ NHRS); 7 km N of Andasibe-Mantadia NP, Waterfall Beanamalao, Circuit Chute Sacre, 18.8282S, 48.4398E, 990 m, leg. J. Bergsten, R. Bukontaite, J.H. Randriamihaja, T. Ranarilalatiana, S. Holmgren, MAD14-84 (2♂, 2♀ NHRS; 2♂, 2♀ UMC); Mantadia National Park, Sahandambo Creek, 18°51.318’S, 48°25.700’E, elev. 933 m, 14-XI-2014, R.W. Sites, sandy w/ veg margins, L-1862 (1♀ UMC); Analanjirofo, Masoala NP, unnamed stream, 15.6832S, 49.9573E, 50 m, 14-II-2018, leg. J. Bergsten & T. Ranarilalatiana, MAD18-35 (1♀ NHRS); Analanjirofo, Masoala NP, Andranobe stream, 15.6798S, 49.9606E, 20 m, 15-II-2018, leg. J. Bergsten & T. Ranarilalatiana, MAD18-38 (1♀ NHRS); Atsinanana, Betampona RNI, Fontsimavo stream above waterfall, 17.9149S, 49.2140E, 300 m, 25-II-2018 leg. J. Bergsten & T. Ranarilalatiana, MAD18-72 (1♂, 2♀ NHRS); Zahamena NP, Antanandava Sect, Alaotra-Mangoro, Manambato River by Camp Bemoara, 17.5126S, 48.7267E, 1050 m, 6-III-2018, rockpools, leg. J. Bergsten & T. Ranarilalatiana, MAD18-77 (1♀ NHRS; 1♀ UMC); Zahamena NP, Antanandava Sect., Alaotra-Mangoro: Manambato River above big cascade, 17.5422S, 48.7225E, 1230 m, 10-III-2018, leg. J. Bergsten & T. Ranarilalatiana, MAD18-106 (2♂ NHRS).

### Additional material examined

Marojejy National Park, 14°26.228’S, 49°46.549’E, elev. 459 m, 8-XI-2014, R.W. Sites, forest stream w/ marginal veg., L-1848 (DNA extracted—1♀ UMC).

## *Tsingala trilobata* NEW SPECIES

urn:lsid:zoobank.org:act:F5F9D1C3-45FD-456C-A725-8CDA1666DC49

(Fig 20)

### Macropterous female

Holotype, length 9.84; maximum width across embolia 5.88. Paratypes (n = 10), length 8.96–10.20 (mean = 9.50); maximum width 5.36–6.16 (mean = 5.72). Overall shape elongate-oval. Dorsally, overall coloration dark-brown with yellowish head, pronotum, proximal 2/3 of embolium (Fig 20A); brown, coarse punctation on head and pronotum; hemelytra mostly dark brown to black, speckled with yellow, and finely punctate. Ventrally, abdomen light-brown, thorax yellow and dark-brown, legs yellowish.

*Head*. length 1.32, maximum width 3.84; inner margin of eyes slightly convergent anteriorly. Synthlipsis at anterior margin 1.80, interocular distance at posteromesal corner 1.96; cuticle laterad of eye expanded. Vertex with heavy, brown punctation forming broad triangle interrupted by yellow posteromedian gap; punctation extending anteriorly as irregular, double median row of coarse punctation, flanked by dark, paramedian row of coalescent punctation; irregular row of setae bordering inner margin of eyes; dark-brown line of pigmentation extending from near posteromedian corner of eye to posteromedian margin of head; three small oval dark markings near posteromedian corner of eye. Anterior margin broadly rounded dorsoventrally (Fig 1C). Labrum with basal sulcus, transverse, distal margin evenly rounded, 2.04× wider than long. Antennae four-segmented, length 0.70, proportions 3, 13, 16, 6.

*Thorax*. Pronotum yellow, scabrous, broad, 2.48× wider than long, length at midline 2.00; maximum width at posterolateral corners 4.96; transverse band across posterior 1/4 devoid of coarse punctures; large, brown punctures becoming smaller and more sparse laterally, with distinct yellow impunctate subrectangular area on midline anterior to transverse band; lateral margin evenly convex, posterolateral corners rounded, posterior margin straight, anterior margin straight; ventrally, basisternum and sternellum mostly medium-brown, propleuron generally yellow and with medium-brown markings near coxa and dark-brown outlining lateral margin of anterior pruinose lobe. Scutellum finely punctate, triangular, black with yellow apically and along lateral margins near anterior corners, with distinctly sinuate lateral margins, 2.02× wider than long, width 3.44, length 1.70. Hemelytra dark-brown with fine, white punctures throughout; with sparse, short, golden setae on corium extending throughout membrane; cuticle irregularly minimized creating dark-brown and yellow color differences and punctures irregularly clustered creating overall speckled appearance. Claval commissure length (to locking mechanism) 1.26. Embolium length 3.36, greatest width 0.80; lateral margin gently curved anteriorly, convexity more pronounced near distal end; yellowish with scattered brown punctures in anterior 2/3, brown in posterior third. Hindwing extending to near posterior margin of tergum V. Metaxyphus broad, with sharp mid-ventral ridge and papillose apex. Coxae brownish-yellow, all other leg segments yellow. Profemur elongate, inflated, anterior margin with dorsal and ventral rows of golden hairs sandwiching one row of short, dark spines. Distal 2/3 of protibia and single-segmented protarsus with dense ventral pad of setae. Propretarsus with short, stout, paired, movable claws. Meso- and metafemora with anteroventral and mid- or posteroventral rows of spinose setae; anteroventral seta rows arcuate from ventral surface to anterior margin, ending in distal fourth; setae number 23–25 on mesofemur and 29–34 on metafemur; mesofemur midventral row with 47–55 setae, with the distalmost 14–16 setae spaced distinctly more tightly; metafemur posteroventral row with 40–42 stout setae. Mesotrochanter and mesofemur with profuse brush of light colored setae on posteroventral margin; brush of setae narrowly set on metatrochanter and metafemur. Elongate, narrow pad of setae on mesotibia and mesotarsomeres 2 and 3. Long golden swimming hairs sparse on mesotibia and tarsus, profuse on metatibia and tarsus. Middle and hind legs with tarsomere 1 extending beneath tarsomere 2. Pretarsal claws long and with slight, even curvature. Leg measurements as follows: foreleg, femur 2.26, tibia 1.50, tarsus 0.44; middle leg, femur 2.28, tibia 1.66, tarsomeres 1–3 0.20, 0.46, 0.48; hind leg, femur 2.80, tibia 2.76, tarsomeres 1–3 0.24, 0.94, 0.78.

*Abdomen*. Dorsally with lateral margins of II–VIII with regular row of short, stout, golden-brown spines. Terga with lateral margins of II–VII each yellow anteriorly, dark brown at posterolateral corner. Ventrally with dense mid-ventral band of elongate setae beginning on III, continuing and becoming more profuse to and including subgenital plate. Posterior margins of mediosternites III–VI symmetrical and nearly straight. Posterior margin of laterosternite V produced at middle with sharply angled, falcate lobe; distal half concave. Posterolateral corners of mediosternite VI produced posteriorly as blunt tabs. Subgenital plate (mediosternite VII) lateral margins slightly convergent, posterior margin with posterolateral and medial weakly pointed lobes; 1.27× wider than long, width 1.32, length 1.04 (Fig 20B).

### Macropterous male

Paratypes (n = 5), length 8.24–9.52 (mean = 8.90); maximum width 4.88–5.80 (mean = 5.42). Similar to female in general structure and coloration with following exceptions: Protarsus two-segmented. Pads of setae more pronounced on pro- and mesotibiae and tarsi. Posterior margins of mediosternite symmetrical, III to IV straight, V deeply concave, VI distinctly concave, VII straight. Tergum VIII with pseudoparameres asymmetrical with posterior margin of left lobe mostly straight to rounded posteromesal corner, right lobe broadly rounded from mesal to lateral margins (Fig 20C). Aedeagus elongate, stout, parallel sided and widest in basal 2/3, left lateral margin in distal third straight and angled toward apex, right lateral margin mostly straight to apex (Fig 20D). Pygophore with anterior margin concave between parameres, slight asymmetry in anterior production of lateral lobes, usually with incipient intermediate lobe, brush of long setae most prominent posteriorly (Fig 20D). Parameres asymmetrical; left paramere with lateral and mesal margins generally straight, both corners broadly rounded (Fig 20E); right paramere narrow, with lateral margin broadly angled creating broadly concave appearance, posterior margin straight, mesal margin with slight concavity, both corners broadly rounded, posteromesal corner slightly deflexed (Fig 20F); elongate setae extending mesad from distal 2/3 of mesal margin and anterior corner of both parameres (Fig 20E and 20F).

### Submacropterous form

Unknown.

### Diagnosis

This species is characterized by the trilobed posterior margin of the female subgenital plate (Fig 20B) and the narrow, somewhat boomerang-shaped right paramere of the male (Fig 20F).

### Discussion

We collected this species from only standing water situations, including pooled water on rocks, vegetated ponds, and mossy rocks near a waterfall. It was collected with *Tsingala humeralis* at Ranomafana National Park. In the recent phylogeny of the family [21], this species was given as *Tsingala* sp. D and was sister to *Tsingala angulata* **n.sp**.

### Etymology

The specific epithet *tri-* (= three) and *lobata* (= lobed) from Latin adjectives are in reference to uniquely three-lobed posterior margin of the female subgenital plate.

### Type material examined

HOLOTYPE ♀: Madagascar, **Fianarantsoa**: N of Ambohimanjaka, Antanavierna River, 20°10.260’S, 47°5.437’E, elev. 1355 m, 1-XI-2014, R.W. Sites, sandy, cascades with pools, irrigation channel, L-1828 (UMC). PARATYPES: same data as holotype (2♂, 1♀ UMC); Ranomafana National Park, 21°14.54508’S, 47°23.5692’E, 2-XI-2014, K.B. Miller, KBM02111401 (4♀ UMC); Ranomafana National Park, Namorona River, 21°15.494’S, 47°25.284’E, elev. 943 m, 3-XI-2014, R.W. Sites, mossy rocks of waterfall, L-1837 (3♂, 1♀ UMC); Ranomafana NP, Research House, 26-XI-1994, 21°15’38"S, 47°25’11"E, M.A. Ivie & D.A. Pollock (1♀ UMC); Ranomafana National Park, 21°15.477’S, 47°25.289’E, elev. 898 m, 3-XI-2014, rock pools in boulders, R.W. Sites, L-1835 (2♀ UMC); Isalo Park, entrance of de Makis canyon, -22.48684S, 45.37668E, 706 m, 12-XI-2012, leg. Bergsten, Bukontaite, Ranarilalatiana, Randriamihaja, MAD12-01 (1♂ NHRS). **Antananarivo**: Analamanga, Manankazo River at RN4 bridge, 18.158S, 47.2104E, 1450 m, 21-XI-2014, leg. J. Bergsten, R. Bukontaite, J.H. Randriamihaja, T. Ranarilalatiana, S. Holmgren, MAD14-75 (2♀ NHRS; 1♀ UMC). **Antsiranana**: Sava, Marojejy NP, Humbert Waterfall, 14.4333S, 49.773E, 550 m, 8–12-XI-2014, leg. J. Bergsten, R. Bukontaite, J.H. Randriamihaja, T. Ranarilalatiana, S. Holmgren, MAD14-48 (1♂ NHRS; 1♂ UMC). **Toamasina**: Betsabora River by RN2 near Antsampanana village, 6 km W of Moramanga, 18.9247S, 48.1828E, 900 m, 24-XI-2014, vegetated forest pond, leg. J. Bergsten, R. Bukontaite, J.H. Randriamihaja, T. Ranarilalatiana, S. Holmgren, MAD14-81 (1♀ NHRS; 1♀ UMC).

### Additional material examined

Antananarivo, Analamanga, Andranofena River by the bridge of RN4, next to Andranofena Sud village, 18.0844S, 47.1776E, 1430 m, 21-XI-2014, colls: J. Bergsten, R. Bukontaite, J.H. Randriamihaja, T. Ranarilalatiana & S. Holmgren, MAD14-74, DNA extracted (1♀ UMC).

## Subfamily Macrocorinae Sites, 2022

### Genus *Macrocoris* Signoret, 1860

*Macrocoris* Signoret 1860: Ann. Soc. Entomol. France 8: 970. Type species: *Macrocoris flavicollis* Signoret, 1860, by monotypy.

This genus was erected by Signoret to contain the widespread mainland African species *M*. *flavicollis* Signoret [20]. The entire genus currently contains nine species, six of which are restricted to mainland Africa and the other three are in a sister clade and restricted to Madagascar. All species of *Macrocoris* are moderately sized to very large for the family and dorsoventrally robust. Reports of *M*. *flavicollis* occurring in Madagascar (e.g., [40]) are not supported by our extensive collecting, nor by museum holdings that we have examined; thus, this species is removed from the list of species of Naucoridae occurring in Madagascar. Until the recent phylogeny of the family [21], *Macrocoris* was contained within the subfamily Naucorinae; however, this subfamily was shown to be polyphyletic and the new subfamily Macrocorinae was established for *Macrocoris* and its sister mainland African genus *Neomacrocoris*. Of the five genera of Naucoridae occurring in Madagascar, *Macrocoris* and *Afronaucoris* differ from the laccocorine genera *Gonioathrix*, *Temnocoris*, and *Tsingala* in having the labrum originating at the front of the head, rather than being set back posteroventrally.

### *Macrocoris distinctus* Bergroth, 1893

(Fig 21)

*Macrocoris distinctus* Bergroth 1893: Rhyn. Aquat. Madag. 12: 212.

*Macrocoris distinctus*: Poisson 1948, Rev. Zool. Bot. Afr. 41: 203 (morphology).

*Macrocoris distinctus*: Poisson 1951, Mem. Inst. Sci. Mad. Ser. A, 5: 101–102 (morphology).

*Macrocoris distinctus*: Poisson 1963, Bull. Inst. Franc. Afr. Noire Ser. A, 25: 1181 (distribution).

*Macrocoris distinctus*: La Rivers 1971, Biol. Soc. Nev. Mem. 2: 74 (catalog).

### Discussion

**Fig 22:**
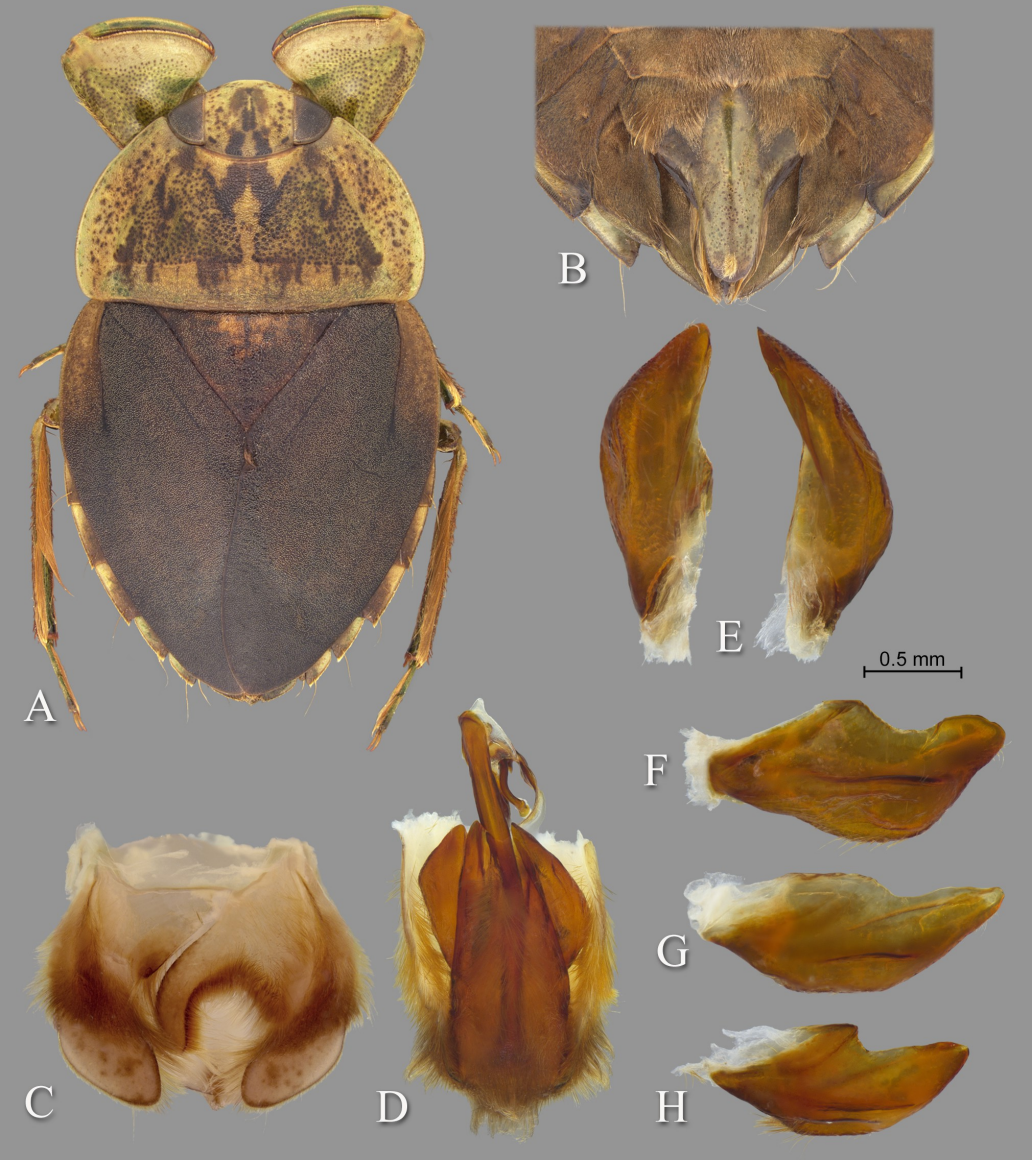
Species of Macrocoris. (A–F) *Macrocoris namorona* **n.sp.** (A) Submacropterous male paratype, (B) female subgenital plate, (C) male 8^th^ abdominal tergum, (D) male genital capsule with proctiger and tergum IX removed, (E) dorsal view of left and right parameres; (F–H) ventral view of left parameres of (F) *M*. *namorona* **n.sp**., (G) *M*. *sikorae*, (H) *M*. *rhantoides*. Scale bar applies only to parameres in Figs E–H.

Nothing has been published on this species after its original description other than a few records of occurrence [26,28], brief notes on morphology [26,34], and inclusion in a catalog of the Naucoridae [22]. This species tends to occur among vegetation in muddy margins of slow streams. A record of this species from Cameroon [41] was later discounted as erroneous and blamed on the similarity of this species to *M*. *flavicollis chariensis* Poisson [26]. The apically widened phallosoma (Fig 22B) is atypical for the species of *Macrocoris* from both Madagascar and mainland Africa that we have examined. The other congeners from Madagascar have slender, arcuate phallosomas similar to that of mainland *M*. *flavicollis*.

### Diagnosis

This is by far the smallest species of the genus in Madagascar. It was reported in the original description to be 7.5 mm in length; however, specimens from our collections were slightly larger. Mean length and width (mm) of our specimens were 7.99 × 4.84 (10 males) and 8.47 × 5.12 (10 females). All specimens that we have examined are macropterous. The wings are dark-brown with lighter mottling, which is especially evident on the clavus (Fig 22A). The scutellum is dark-brown to black, with dark-reddish regions laterally in most specimens. The head and pronotum are yellowish-brown with great variation in the extent of dark-brown maculation on the pronotum (Fig 22A). Ventrally, the abdomen is rufous, propleuron dark-brown and black medially and yellow-brown laterally, and meso- and metasterna mostly black.

### Published records

Betioky [26], Tananarive, Tamatave, Tuléar [28].

### Material examined

**Antsiranana**: unnamed stream, 14°3.552’S, 50°1.622’E, elev. 65 m, 10-XI-2014, R.W. Sites & K. Miller, rocky stream w/ veg margins, L-1853 (1♂ UMC); Andranonakoho, 12.92734S, 49.16309E, 154 m, 3-XII-2012, leg. Bergsten, Bukontaite, Ranarilalatiana, Randriamihaja, MAD12-41 (1♀ NHRS); Diana, Ankarana National Park, Riviere Vert, S12.92653, E49.09687, 42 m, 1-XII-2012, leg. Bergsten, Bukontaite, Randriamihaja, Ranarilalatiana, MAD12-38 (1♂, 1♀ UMC); Diana, River Mahavanona, 25 km S from Diego Suarez, S12.45538, E49.37619, 70 m, 10-XII-2012, leg. Bergsten & Bukontaite, MAD12-60 (1♂, 1♀ NHRS). **Antananarivo**: Andranofena River at Andranofena Sud village, 18.0844S, 47.1776E, 1430 m, 21-XI-2014, leg. Bergsten, Bukontaite, Randriamihaja, Ranarilalatiana, Holmgren, MAD14-74 (1♂, 1♀ NHRS); Vakinankaratra, Manjakatampa Stn., Forestiera, Lac Froid, S19.34295, E47.3390, 1640 m, 3-XI-2011, J. Bergsten, MAD11-15 (4♂, 4♀ NHRS; 2♂, 2♀ UMC); Museum Paris, Madagascar Tananarive, R. Decary 1921 / 28367, 19 det by H.B. Hungerford / Macrocoris distinctus Bergroth det. R.W. Sites 2021 (1♀ SEMC); Museum Paris, Madagascar Tananarive, R. Decary 1921 / Europ. Trip 1928, No. 28367, H.B. Hungerford / 28367, 19 det by H.B. Hungerford / Macrocoris distinctus Berg 19 det by H.B. Hungerford / Macrocoris distinctus Bergroth det. R.W. Sites 2021 (1♂ SEMC). **Fianarantsoa**: Amoron’i Mania, Talaviana River, 37 km S of Antsirabe, S20.1711, E47.091, 1360 m, 1-XI-2014, rocks and sand, leg. Bergsten, Ranarilalatiana, Holmgren, MAD14-01 (1♀ NHRS); Ihorombe, 4 km from Ambinda Village, Lake Ambiaravy, S22.54076, E46.32273, 761m, 6-XII-2013, leg. Randriamihaja & Ranarilalatiana, MAD13-43 (1♂ NHRS); Atsimo Antsinanana, R.S. Manombo, Parcelle I, S23.01388, E47.72425, 16 m, 16-XII-2013, pools on trail, leg. Randriamihaja & Ranarilalatiana, MAD13-76 (2♂, 1♀ NHRS); N of Ambohimanjaka, Antanavierna River, 20°10.260’S, 47°5.437’E, elev. 1355 m, 1-XI-2014, R.W. Sites, sandy, cascades with pools, irrigation channel, L-1828 (2♂, 3♀ UMC); same locality, 5-XI-2014, R.W. Sites, shallow, sandy w/ vegetated margins, L-1846 (15♂, 21♀ UMC); Ranomafana National Park, Namorona River at Namoroma Village, 21°15.738 S, 47°27.264’ E, elev. 621 m, 2-XI-2014, R.W. Sites, silty with cobble & marginal grasses, L-1830 (1♀ UMC); Ranomafana National Park, unnamed stream nr confl. w/Namorona Riv., 21°14.425’S, 47°23.649’E, elev. 1135 m, 2-XI-2014, R. W. Sites, marginal veg, open canopy, L-1831 (1♂ UMC); Ranomafana National Park, Tomaro River at Ambatolahy, 21°15.024’S, 47°25.756’E, elev. 872 m, 3-XI-2014, R. W. Sites, rocky with vegetated margins, L-1842 (1♀ UMC); Isalo Menamaty River, 757 m, N-22°33.001, E45°24.074, 11-V-2006, leg Bergsten et al, degraded river w/ lots of veg., P41AM01, BMNH(E)741830 (2♂, 1♀ NHRS). **Toamasina**: Antsinanana, RN2, Ambodivoanio, 41 km N Toamasina, S17.87002, E049.46249, 20 m, 16-XI-2011, pond w vegetation, leg. J. Bergsten, R. Bukontaite, T. Ranarilalatiana, & J.H. Randriamihaja, MAD11-59 (1♀ NHRS); Alaotra, Mangoro, RNs, Mangoro River, 10 km W of Moramanga, S18.92438, E048.18273, 940 m, 6-XI-2011, river & pools, leg. Bergsten, Bukontaite, Ranarilalatiana & Randriamihaja, MAD11-21 (6♂, 2♀ NHRS; 1♂, 1♀); Analanjirofo, by RN2, 17 km N Fenerive, S17.25577, E049.40646, 20 m, 16-XI-2011, pools in rocks, leg. Bergsten, Bukontaite, Ranarilalatiana & Randriamihaja, MAD11-56 (1♂ UMC); Betsabora River by RN2 near Antsampanana village, 6 km W of Moramanga, 18.9247S, 48.1828E, 900 m, 24-XI-2014, vegetated forest pond, leg. J. Bergsten, R. Bukontaite, J.H. Randriamihaja, T. Ranarilalatiana, S. Holmgren, MAD14-81 (1♂ NHRS); Maroantsetra, 4-V-2010, M. Randrianandrafana (1♂ UMC); Antsinanana, RN2, river 13 km N Toamasina, S18.01965, E049.40129, 30 m, 14-XI-2011, leg. J. Bergsten, R. Bukontaite, T. Ranarilalatiana, & J.H. Randriamihaja, MAD11-53 (1♀ NHRS); Antsinanana, RN2, Onibe River, 60 km N Toamasina, S17.65545, E049.4737, 20 m, 15-XI-2011, pond w vegetation, leg. J. Bergsten, R. Bukontaite, T. Ranarilalatiana, & J.H. Randriamihaja, MAD11-54 (5♂ UMC). **Toliara**: NW Ft Dauphin, rice paddies w/ water running under road, 34 m, 19-V-2006, P54F, leg. Bergsten et al, N-24°49.472, E46°51.974, BMNH(E)742268 (1♂ NHRS).

## *Macrocoris namorona* NEW SPECIES

urn:lsid:zoobank.org:act:CAA3B0F5-8E30-4777-AB85-6AF8A15E69EE

(22A–F)

### Submacropterous male

Holotype, length 14.88; maximum width across embolia 9.16. Paratypes (n = 10), length 13.92–14.88 (mean = 14.60); maximum width 8.00–8.96 (mean = 8.58). General shape elongate, broadly rounded anteriorly and posteriorly, dorsoventrally robust, and widest across embolia (Fig 22A); very large for the genus, overall coloration dorsally yellowish-brown anteriorly with dark brown scutellum and hemelytra; ventral surface mostly brown, legs and lateral part of propleura yellow; yellowish coloration appearing green in live specimens.

*Head*. Length 2.68, maximum width 4.06. Yellow with dark brown spots, paired paramedian dark brown triangular markings at posterior margin, dark brown median stripe formed by two confluent rows of spots; projecting beyond eyes 15% of head length. Eyes dark brown; twice as long as wide; inner margins slightly concave, lateral margins rounded; synthlipsis anteriorly 2.16; vertex with posterior margin convex, meeting posteromedian corner of eye. Labrum yellowish-brown, transverse, broadly rounded distally, width/length 1.20/0.66. Maxillary plate yellow, elongate, extending from labrum to near base of antenna. Labium with three visible segments, basal two segments yellowish-brown, distal segment dark brown, extending 0.70 beyond labrum. Antenna four-segmented; 0.78; hirsute; pedicel broadening distally with white, oval, glabrous area on anterior surface; segments three and four hirsute, cylindrical, similar in shape; not extending beyond lateral margin of eye; relative lengths 7, 16, 11, 10.

*Thorax*. Pronotum broad, highly convex, brownish-yellow; scattered brown punctation throughout, brown W-shaped marking at anterior midline; wide transverse band along posterior margin with irregular series of brown, longitudinally elongate markings, otherwise mostly brownish-yellow; maximum width 8.00, length at midline 3.60; posterior margin broadly, shallowly convex; lateral margins evenly convex, convergent anteriorly, distinctly explanate; posterolateral corners rounded. Scutellum dark-brown, lighter near anterior corners; densely punctate; width 4.60, length at midline 2.80; lateral margins nearly straight, slightly concavity anteriorly. Hemelytra densely punctate throughout, irregularly mottled with medium and dark brown, length 9.92 (chord measurement). Clavus distinct, with small yellow marking at commissure. Embolium with sparse golden setae, posterior delimiting suture partially suppressed, lateral margin convex throughout, anterior 2/3 yellowish laterally, maximum width 1.36, length 4.40. Hemelytra not attaining tip of abdomen, half of lateral lobes of abdominal segment VIII exposed; membrane of underlapping wing (left) punctate and pruinose. Hindwings reduced, extending to middle of tergum III. Ventrally, prosternum with mid-ventral carina between procoxae. Propleuron laterally with extensive yellow, glabrous area extending 60% distance to mesal margin; brown hirsute area mesad of yellow, glabrous area and along posterior margin; propleura widely separated at midline. Mesobasisternum with median carina covered with elongate brown setae; meso- and metasterna mostly medium brown, pruinose, with scattered setae.

*Legs*. All segments mostly brownish-yellow except brown protarsus and apices of meso-and metatarsus. Profemur with scattered brown spots on ventral and dorsal surfaces; anterior margin with dense pad of elongate, golden-brown setae, but without intervening spines; posterior margin with narrow band of spines in basal half. Protibia and tarsus with flattened inner surface, tarsus one-segmented, single claw minute. Middle and hind coxae densely covered with short, pale, recumbent setae. Metasternellum (= metaxyphus) with pronounced transverse and longitudinal carinae, thus resembling head of Phillips screwdriver directed posteroventrad. Meso- and metafemora each with posteroventral row of brown, peglike spines; spines more elongate distally on mesofemur; meso- and metafemora and trochanters with posterodorsal row of pale, elongate setae. Meso- and metatibiae with longitudinal rows of stout, reddish-brown spines and two transverse rows of long, stout spines at apices; meso- and metatibiae and tarsi with long, golden brown swimming hairs. Meso- and metapretarsal claws paired, slender, evenly curved, with basal tooth. Leg measurements as follows: foreleg, femur 4.16, tibia 3.36, tarsus 0.68; middle leg, femur 3.76, tibia 3.20, tarsomeres 1–3 0.26, 0.54, 0.84; hind leg, femur 4.52, tibia 5.28, tarsomeres 1–3 0.38, 1.02, 1.04.

*Abdomen*. Margins of terga III–VIII exposed laterally beyond hemelytra; III–V each yellow anteriorly, brown posteriorly, VI–VIII mostly brownish-yellow; lateral margins smooth, with dense fringe of pale setae; posterolateral angles of II–V squared, VI–VII acute but not sharp, VIII rounded. Tergum V extended posteriorly, posterior margin broadly lobed on each side of midline. Tergum VIII right medial lobe strongly curved and heavily setose on posterior margin (Fig 22C). Proctiger covered with pale brown setae, acuminate apically. Ventrally brown and covered with dense, fine pile of short, recumbent setae, except narrow marginal glabrous band; mediosternites also with elongate, erect, brown setae. Sternum V posterior margin asymmetrically concave and mediosternite VI displaced asymmetrically to left. Genital operculum evenly rounded. Pygophore brown, elongate setae generally scattered and with a dense brush posteriorly (Fig 22D). Phallosoma slender, straight in basal half, then angled left in distal half before deflexing with articulated vesica and endosoma (Fig 22D). Parameres large, 3-dimensionally robust, elongate and flanking phallosoma to end of pygophore (Fig 22D). Left paramere in ventral view with mesal margin deeply concave at middle and broadly rounded distally (Fig 22F); right paramere gently curved and cupped medially at apex (Fig 22E).

### Submacropterous female

Paratypes (n = 10), length 14.40–15.36 (mean = 15.00); maximum width 8.64–9.28 (mean = 8.84). Similar to male in general structure and coloration with following exceptions: Abdominal terga not modified. Abdominal mediosternites symmetrical. Subgenital plate (sternite VII) broad basally, narrowing abruptly at midlength to elongate, laterally cupped, tonguelike lobe, dense pile of short brown setae in basal third, glabrous in distal third, tuft of elongate golden brown setae in middle at apex (Fig 22B); subgenital plate length at midline 3.04, width at base 3.32.

### Discussion

This large species was collected only in the Namorona River and its tributaries in Ranomafana National Park. Specifically, it occurred in grassy, vegetated margins where the current was moderate to strong.

### Diagnosis

This species is nearly indistinguishable from the congeners *M*. *rhantoides* and *M*. *sikorae* and was recovered as sister to *M*. *rhantoides* in the recent molecular phylogeny [21]. We have found only the left paramere to be a reliable diagnostic attribute by which to distinguish among these species. More specifically, the mesal margin in ventral view has a deep concavity at the middle and is broadly rounded distally in *M*. *namorona* **n.sp**., whereas the mesal margin is mostly straight beyond the base in *M*. *rhantoides* and *M*. *sikorae*. In addition, *M*. *rhantoides* is somewhat smaller than the other two. The fourth species of *Macrocoris* in Madagascar, *M*. *distinctus*, is easily distinguished because it is much smaller and rounder than this group of three closely related species.

### Etymology

The species is named for the river within which the type series was collected.

### Type material examined

HOLOTYPE ♂: Madagascar, Fianarantsoa: Ranomafana National Park, Namorona River at Namorona Village, S21°15.738’, E47°27.264’, elev. 621 m, 2-XI-2014, R.W. Sites, marginal grasses, L-1830 (UMC). PARATYPES: same data as holotype (9♂, 6♀ UMC); same locality, 4-XI-2014, R.W. Sites, marginal overhanging grasses in current, L-1843 (15♂, 20♀ UMC; 5♂, 5♀ NHRS; 5♂, 5♀ USNM); Ranomafana National Park, Matsiara Ambony, S21.2395°, E47.3947°, 1130 m, forest stream with sandy bottom, 2-XI-2014, Bergsten, Ranarilalatiana, Holmgren, MAD14-04 (1♀ NHRS); Matsiatra, Ambony, Ranomafana NP, Namorona River 2 km from Vohiparara, S 21.2408 E 047.39405, 1120 m, 30-X-2011–31-X:-2011, crayfishtrap in river with muddy bottom, Leg. J. Bergsten, R. Bukontaite, T. Ranarilalatiana, & J.H. Randriamihaja, MAD11-04 (1♂ NHRS); Ranomafana National Park, Amboditanimena River at Amboditanimena, nr. confluence with Namorona River, S21°13.584’, E47°22.182’, elev. 952 m, 3-XI-2014, R.W. Sites, small sandy stream w leafpacks & dense canopy, L-1841 (1♂ UMC); Ranomafana National Park, unnamed stream, S21°14.289’, E47°23.681’, elev. 1145 m, 2-XI-2014, R.W. Sites & J. Bergsten, shallow, shaded, sandy substrate, L-1833 (1♂ UMC).

## *Macrocoris rhantoides* Bergroth, 1893

(Fig 23)

*Macrocoris rhantoides* Bergroth 1893: Rhyn. Aquat. Madag. 12: 211.

*Pseudambrysus rhantoides*: Montandon 1913, Bull. Soc. Rom. Stiinte 22: 331.

*Pseudambrysus rhantoides*: Poisson 1951, Mem. Inst. Sci. Mad. Ser. A, 5: 100.

*Pseudoambrysus rhantoides*: Poisson 1963, Bull. Inst. France Afr. Noire Ser. A, 25: 1182.

*Macrocoris rhantoides*: La Rivers 1971, Biol. Soc. Nev. Mem. 2: 7 (catalog).

**Fig 23:**
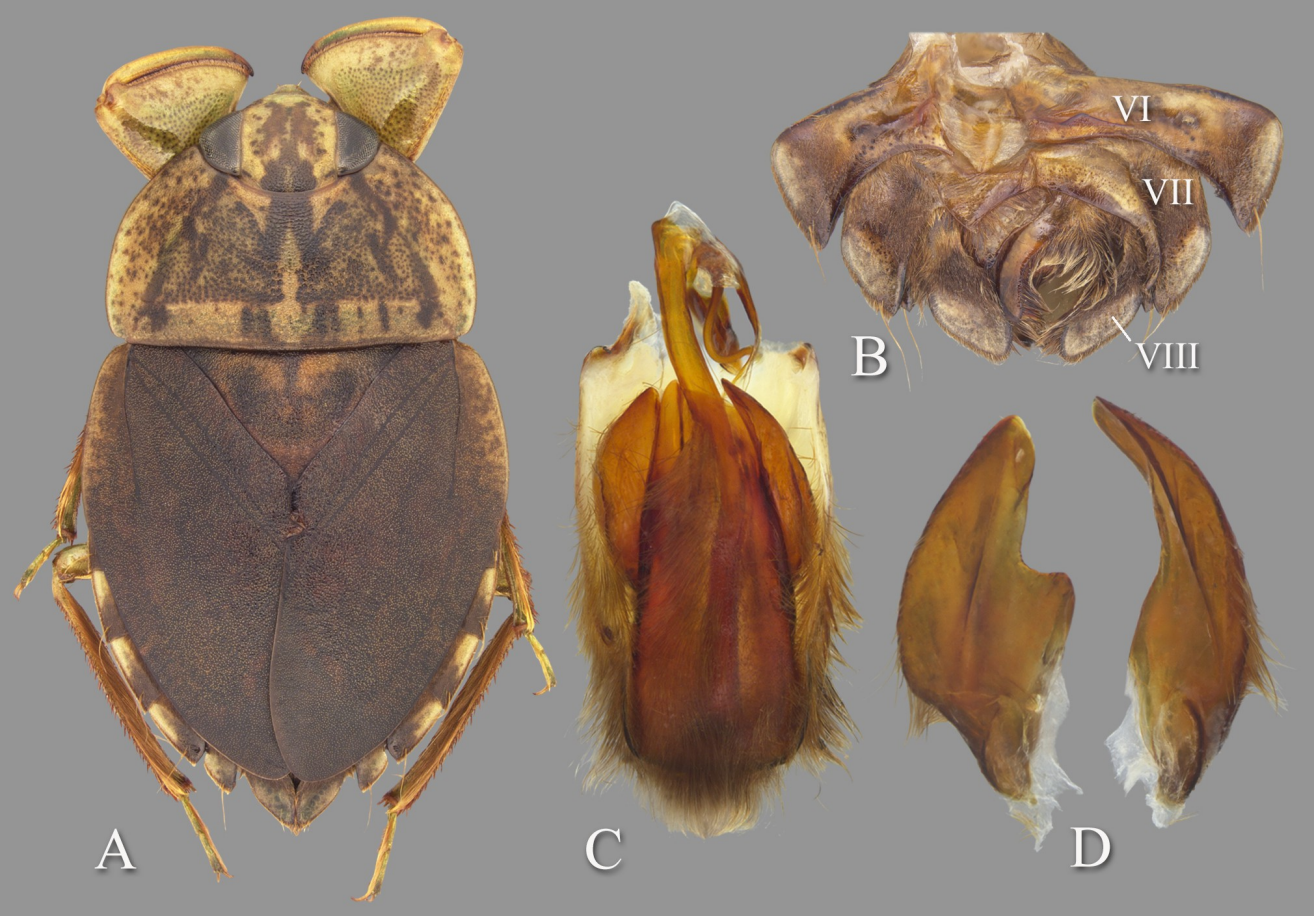
Macrocoris rhantoides. (A) Submacropterous female, (B) male terminal abdominal terga, (C) male genital capsule with proctiger and tergum IX removed, (D) dorsal view of left and right parameres.

### Discussiony

This species is much less frequently collected than *M*. *sikorae*. The few specimens of *M*. *rhantoides* we collected included adults and immatures from among the marginal vegetation of a rocky stream. Live specimens tend to be moderately dark green anteriorly with dark grey wings. Montandon [42] established the genus *Pseudambrysus* based on a superficial resemblance to the American genus *Ambrysus*, but with little comparative material in collections at the time. After more specimens were available to him for examination, he recognized clear distinctions between these two genera and between *Pseudambrysus* and *Macrocoris* [43]. Further, he also realized his *Pseudambrysus fairmairei* was already described as *Macrocoris sikorae*; thus, he synonymized the species but transferred it to *Pseudambrysus* [43]. In that work, he also transferred *Macrocoris rhantoides* to *Pseudambrysus*. Poisson continued to recognize *Pseudambrysus* (and *Pseudoambrysus* [sic]) as a valid genus [26,28], which continued until La Rivers published a catalog of the species in which the two species were transferred back to *Macrocoris* [22].

### Diagnosis

This species is much larger than *M*. *distinctus* and slightly smaller than *M*. *namorona* **n.sp**. and *M*. *sikorae*, with which it shares a remarkably similar appearance. Mean length and width (mm) of our specimens are 12.36 × 7.20 (2 males) and 12.96 × 7.60 (1 female). Other than size, the most distinctive difference separating it from these two congeners is the shape of the male left paramere (Figs 2H and 23D).

### Published records

Ankarafantsika [26], Nossi-Bé [28].

### Material examined

**Antsiranana Province**: ca. 40 km W of Sambava, 14°28.186’S, 49°55.433’E, elev. 102 m, 9-XI-2014, R. W. Sites, rocky stream w/ overhanging veg, L-1851 (DNA extracted—1♂ UMC; 1♀ UMC); River Amposatelo, 4 km from Anilotra, S-12.9261, E49.09526, 43 m, 2-XII-2012, leg. Bergsten, Bukontaite, Ranarilalatiana, Randriamihaja, MAD12-40 (1♀, 5 nymphs NHRS); Vakinankaratra: Manjakatompo Stn. Forestière, Poste, S19.354194, E047.308083, 1796 m, 24-I-2012, lake w grass, leg. T. Ranarilalatiana & J. H. Randriamihaja, MJK12-14 (1♀ UMC). **[Mahajanga Province]**: Marovoay Western Madagascar Province: Majunga River, Ikopa, 1927 & 28 (1♂ UMC).

## *Macrocoris sikorae* Bergroth, 1893

(Fig 24)

*Macrocoris sikorae* Bergroth 1893: Rhyn. Aquat. Madag. 12: 211–212.

*Pseudambrysus fairmairei* Montandon 1897c: Verh. Zool. Bot. Ges. Wien 47: 10–11.

*Macrocoris (Pseudambrysus) sikorae*: Montandon 1900, Term. Fuz. 23: 422 (synonymy).

*Pseudambrysus sikorae*: Montandon 1913, Bull. Soc. Rom. Stiinte 22: 331 (synonymy).

*Pseudambrysus sikorae*: Poisson 1951, Mem. Inst. Sci. Madag. Ser. A, 5: 99.

*Pseudoambrysus sikorae*: Poisson 1963, Bull. Inst. France Afr. Noire Ser. A, 25: 1183.

*Macrocoris sikorae*: La Rivers 1971, Biol. Soc. Nev. Mem. 2: 74 (catalog, synonymy).

**Fig 24:**
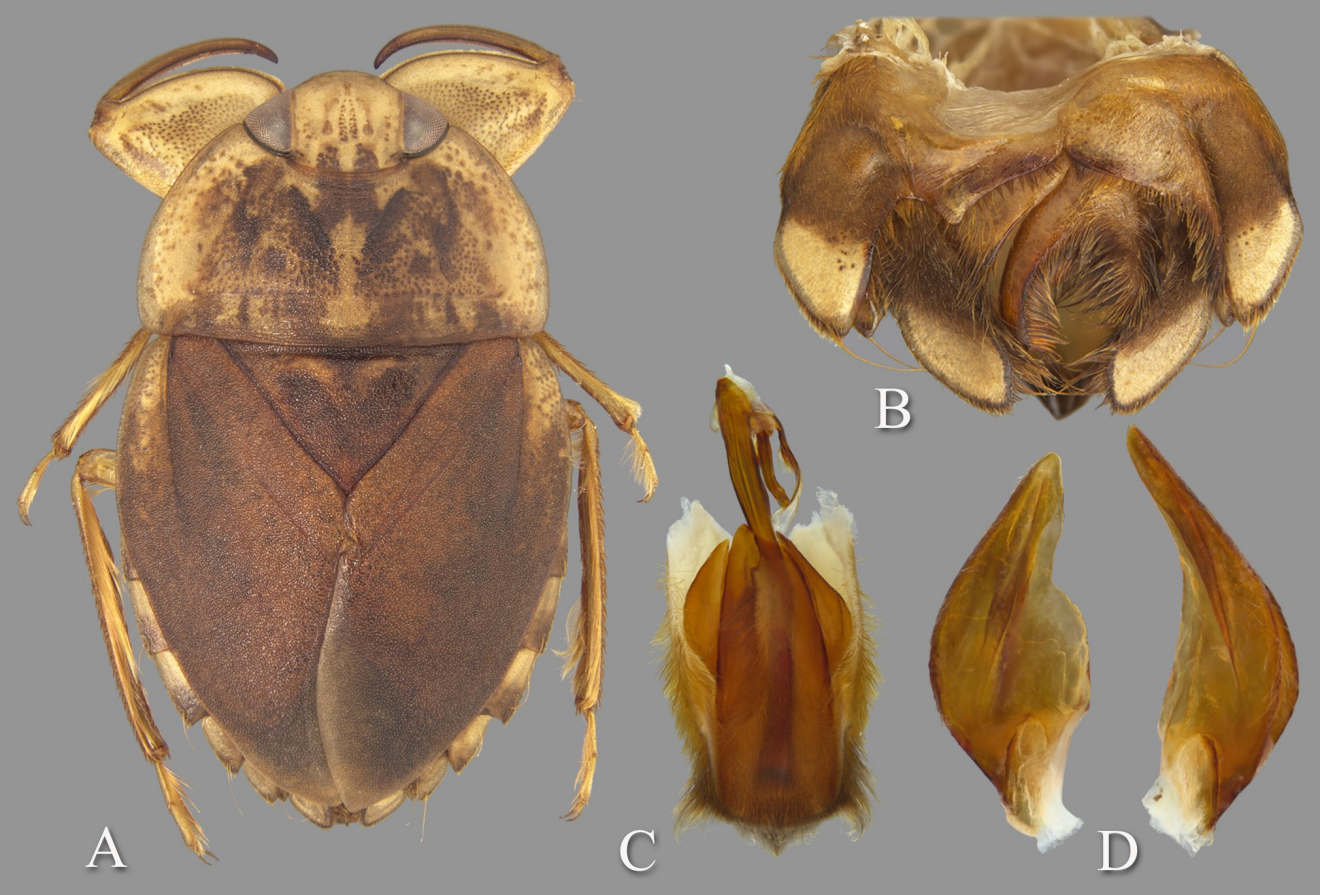
Macrocoris sikorae. (A) Submacropterous male, (B) male abdominal terga VII-VIII, (C) male genital capsule with proctiger and tergum IX removed, (D) dorsal view of left and right parameres.

### Discussion

In 1893, Bergroth described the three species of *Macrocoris* known from Madagascar at the time [39]. Four years later, Montandon must have been unaware of Bergroth’s species when he erected a new genus, *Pseudambrysus*, to contain *P*. *fairmairei* [42], which he would later synonymize at the species level with *M*. *sikorae*, but retain in *Pseudambrysus* as *P*. *sikorae* [43]. Live specimens tend to be moderately green to brown anteriorly with grey to brown wings.

### Diagnosis

This is among the largest of the Madagascar saucer bugs; mean length and width (mm) of our specimens are 13.12 × 7.61 (10 males) and 14.13 × 8.22 (10 females). Its general appearance is nearly identical to that of *M*. *namorona* **n.sp.** and *M*. *rhantoides*, although significantly larger than the latter. The most distinctive difference separating it from these two congeners is the shape of the male left paramere (Figs 22G and 24D).

### Published records

Lac Tritriva [26], Tuléar [28].

### Material examined

Madagasacar, **[Mahajanga]**: Marovoay Western, Majunga River, Ikopa, 1927 & 28 (1♂, 2♀ UMC; 3♂, 2♀ SEMC). **Toamasina**: Analanjirofo Masoala NP, rainforest, pristine forest stream, 3 hours walk E of Andranobe camp, 15.6735S, 49.9886E, 500 m, 16-II-2018, leg. J. Bergsten & T. Ranarilalatiana, MAD18-44 (2♂, 5♀, 9 nymphs NHRS; DNA extracted—1♂ UMC; 2♂, 3♀ UMC); Analanjirofo, Masoala NP, Andranobe stream, ~400 m upstream camp, 15.6798S, 49.9606E, 20 m, 15-II-2018, leg. J. Bergsten & T. Ranarilalatiana, MAD18-38 (1♂, 1-5th instar NHRS); Analanjirofo, Masoala NP, Andranobe stream, backwater, ~500 m upstream camp, 15.6794S, 49.9620E, 30 m, 15-II-2018, leg. J. Bergsten & T. Ranarilalatiana, MAD18-39 (2♂, 1♀, 6-5th instars NHRS; 1♂, 1♀ UMC); Alaotra-Mangoro Zahamena NP, Antanandava Sect., Manambato river by Camp Bemoara, 17.5126S, 48.7267E, 1050 m, 6-III-2018, leg. J. Bergsten & T. Ranarilalatiana, MAD18-78 (1♀ NHRS; 1♂ UMC); Mantadia National Park, Sahandambo Creek, 18°51.318’S, 48°25.700’E, elev. 933 m, 14-XI-2014, R.W. Sites, sandy w/ veg margins, L-1862 (6♀ UMC); Mantadia National Park, Andranomanamponga Ck., 18° 49.784’S, 48° 25.917’E, elev. 948 m, 14-XI-2014, R.W. Sites, sand/rock/gravel/veg undercuts, L-1863 (3♀, 4 nymphs UMC); Mantadia National Park, Sahabe River, 18°47.910’S, 48°25.580’E, elev. 940 m, 14-XI-2014, R.W. Sites, sandy w/ undercuts, L-1865 (1♀, 1-5th instar UMC); Betsabora River by RN2 near Antsampanana village, 6 km W of Moramanga, 18.9247S, 48.1828E, 900 m, 24-XI-2014, vegetated forest pond, leg. J. Bergsten, R. Bukontaite, J.H. Randriamihaja, T. Ranarilalatiana, S. Holmgren, MAD14-81 (1♀ NHRS).

## Subfamily Naucorinae Stål, 1876

### Tribe Afronaucorini Sites, 2022

Genus *Afronaucoris* Sites, 2022

*Afronaucoris* Sites 2022: Zool. J. Linn. Soc. 195: 1275–1277. Type species: *Afronaucoris obscuratus* (Montandon, 1913), by original designation.

*Afronaucoris* was recently erected to contain the Afrotropical species formerly held in *Naucoris* Fabricius, 1775, which was shown to be polyphyletic with four distinct, independent clades [21]. *Afronaucoris* excludes the nominate, Palearctic species *Naucoris maculatus* Fabricius, which occurs in northern Africa. In Madagascar, *Afronaucoris* is the only genus of the subfamily Naucorinae. It can be distinguished from *Gonioathrix*, *Temnocoris*, and *Tsingala*, all members of Laccocorinae, by the mouthparts arising from the anterior margin of the head, rather than being set back posteroventrally. It can be distinguished from all species of *Macrocoris* (Macrocorinae) most readily by its small size (body length ≤ 7.7 mm); the smallest species of *Macrocoris* is ≥ 7.9 mm.

Only two species of *Afronaucoris* occur in Madagascar. They can be distinguished from each other most notably by the surface texture of the pronotum. In *A*. *parvulus*, the pronotum is smooth and glossy; indications of punctation are only negligibly dimpled, if at all. In contrast, the pronotum of *A*. *madagascariensis* has punctures that are clearly dimpled and the entire surface has a more matte appearance in comparison. Orienting the light source to visualize surface texture and reflection of dry specimens under the microscope is necessary to confidently identify specimens. The two species are not distinguishable by color or patterns of color. *Afronaucoris madagascariensis* is on average slightly larger and proportionately wider. Most or all of our *A*. *madagascariensis* were collected syntopically with series of *A*. *parvulus*.

## *Afronaucoris madagascariensis* (Montandon, 1899)

(Fig 25)

*Naucoris madagascariensis* Montandon 1899: Bull. Mus. Hist. Natur. 1899(2): 81.

*Naucoris madagascariensis*: La Rivers 1971, Biol. Soc. Nev. Mem. 2: 72 (catalog).

*Afronaucoris madagascariensis*: Sites 2022, Zool. J. Linn. Soc. 195: 1277.

**Fig 25:**
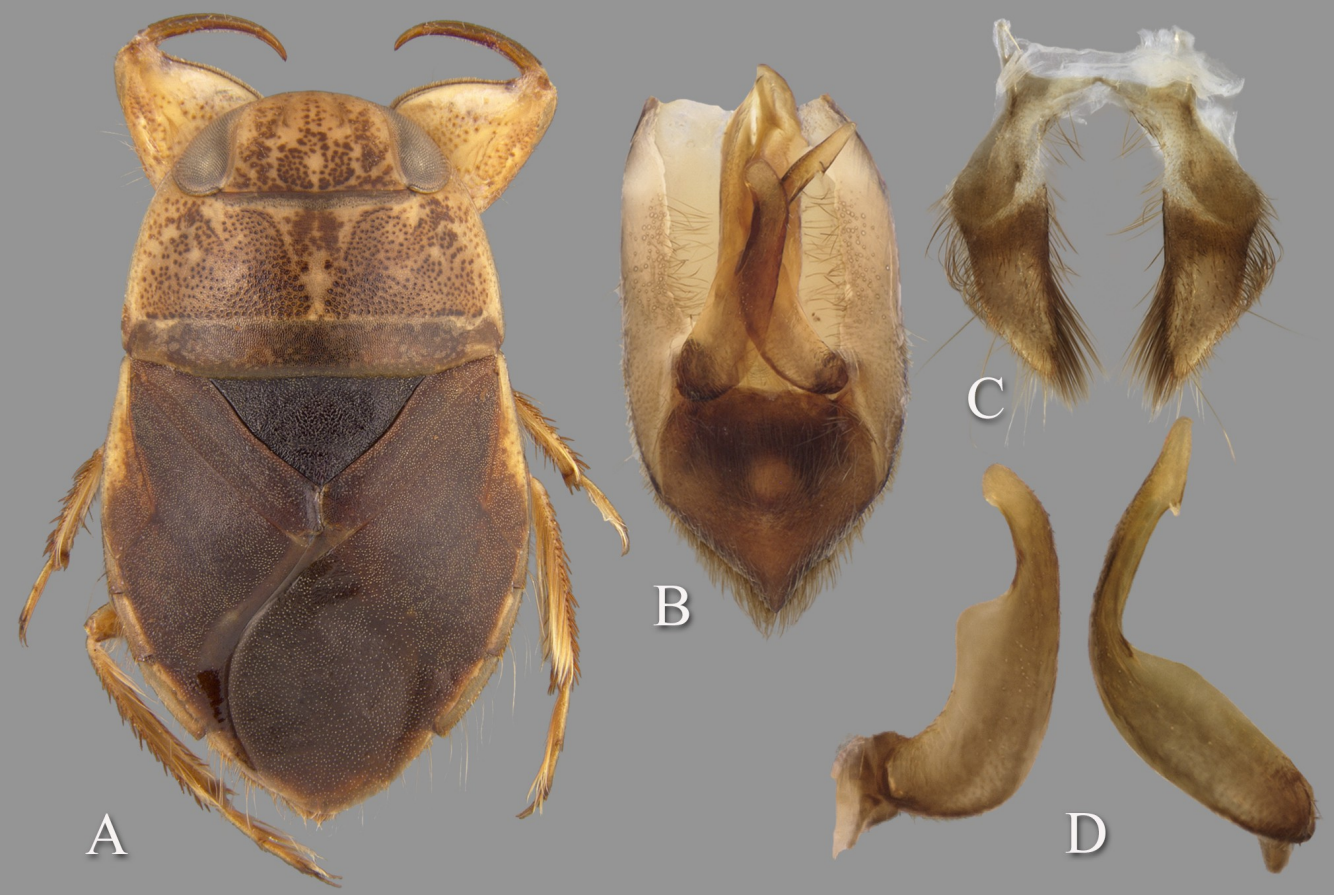
Afronaucoris madagascariensis. (A) Male habitus, (B) male genital capsule with proctiger and tergum IX removed, (C) male 8^th^ abdominal tergum, (D) dorsal view of left and right parameres.

### Lectotype designation

We examined the type series of seven syntypes specimens on loan from the MNHN. One large female is labeled "type" and the other six specimens as cotypes. Because the original description did not designate any specimen as the type, we consider all seven specimens to be syntypes. The female labeled type is here designated as the lectotype in order to fix the identity of the species and the other six specimens are paralectotypes. One pair of paralectotypes is transferred to UMC as an exchange.

### Discussion

Nothing has been published on this species after its original description other than its inclusion in a catalog of the world fauna of Naucoridae [22] and its recent transfer from *Naucoris* to the new genus *Afronaucoris* [21]. Surprisingly, Poisson did not treat this species in any of his series of papers on the aquatic and semiaquatic Heteroptera of Africa. Specimens from our sampling were collected from the vegetated margins of slow streams and lentic waterbodies.

### Diagnosis

This small species was listed in the original description as length 6.9–8.5, width 3.8–4.9 [44]. Our specimens and the series of six paralectotypes measure length 6.56–8.08 (mean = 7.21); maximum width 3.84–4.64 (mean = 4.26). The lectotype measures 8.36 × 4.80. This species differs from *A*. *parvulus* in the surface texturing of the pronotum; the punctures are clearly dimpled and along with additional fine irregularities, the surface has an overall matte appearance (Fig 25A); male genitalic differences between them are negligible (Fig 25B–25D).

### Published records

No published records are available.

### Type material examined

LECTOTYPE, ♀: Type / Museum Paris, Madagascar, P. Camboue 346–94 / Naucoris madagascariensis 1899, Montand., type 1899, Bull. Mus. Paris. 1899 (MNHN). PARALECTOTYPES: Museum Paris, Madagascar, P. Camboue 346–94 / Naucoris madagascariensis 1899, cotype Montand (2♂, 1♀ MNHN); same but with extra label: exchange w/ MNHN (1♂, 1♀ UMC); same as MNHN paralectotypes, but also Bull. Mus. Paris. 1899 (1♂ MNHN).

### Additional material examined

**Antananarivo:** Vakinankaratra, Manjakatompo Stn. Forestiére, Analafandriana, S19.35806, E047.31401, 1730 m, 3-XI-2011, stream with wet field, leg. J. Bergsten, R. Bukontaite, T. Ranarilalatiana, & J.H. Randriamihaja, MAD11-13 (1♂, 1♀ UMC; 1♀ NHRS); Vakinankaratra, Manjakatompo Stn. Forestiére, 2 km NE Amparandraindrisa, S19.36067, E047.30098, 1770 m, 5-XI-2011, pond and stream, leg. J. Bergsten, R. Bukontaite, T. Ranarilalatiana, & J.H. Randriamihaja, MAD11-18 (1♀ NHRS); Vakinankaratra: Manjakatompo Stn. Forestiére, 500 m E Lac Froid, S19.34485, E047.33381, 1620 m, 4-XI-2011, pond and inlet stream, leg. J. Bergsten, R. Bukontaite, T. Ranarilalatiana, & J.H. Randriamihaja, MAD11-16 (1♀ NHRS); Tananarive Madagascar Prof C. Lamberton purch Nov 1931 (1♂ SEMC); 12-2-31 / Museum Paris, Madagascar Tananarive, R. Decary 1921 / Naucoris madagascariensis det. by H.B. Hungerford, c with cotype (1♂, 1♀ SEMC). **Antsiranana**: Manara River at Antirabe Nord, 13°58.522’ S, 49°57.845’ E, elev. 32 m, 10-XI-2014, R.W. Sites, rock pools & vegetation, L-1852 (1♀ UMC); unnamed stream, 14°3.552’S, 50°1.622’E, elev. 65 m, 10-XI-2014, R.W. Sites & K. Miller, rocky stream w/ veg margins, L-1853 (2♀ UMC); 14°11.038’ S, 50°1.500’ E, elev. 42 m, 10-XI-2014, R.W. Sites, G. Gustafson, K. Miller, flooded rice paddy & overflow, L-1854 (2♂, 1♀ UMC); Bemarivo River at Ambudipunt, 14°12.292’ S, 50°3.141’ E, elev. 19 m, 10-XI-2014, R.W. Sites, vegetated margins, L-1855 (1♀ UMC). **Toamasina**: Alaotra, Mangoro Reg, 150 m E of entrance to Andasibe-Mantadia N.P., 18.9355S, 4834166E, 930 m, 27-XI-2014, Analamazoatra, leg. Bergsten, Ranarilalatiana, Bukontaite, Randriamihaja, MAD11-14 (1♂ UMC).

## *Afronaucoris parvulus* (Signoret, 1860)

(Fig 26)

*Naucoris parvulus* Signoret 1860: Ann. Soc. Entomol. France 8: 970.

*Naucoris parvulus*: Stål 1865, Hemip. Afr. 3: 176–177.

*Naucoris parvulus*: Walker 1873, Cat. Spec. Hem. Heterop. Coll. Brit. Mus. 8: 184.

*Naucoris parvulus*: Stål 1876, Enum. Hemip. 14: 145.

*Naucoris hydroporoides* Bergroth 1893: Rev. Ent. 12: 210–211 (syn. by Montandon 1899, Bull. Mus. Hist. Natur. 1899(2): 81).

*Naucoris parvulus*: Poisson 1948, Mem. Inst. Sci. Madag. Ser. A 2: 106–107 (distribution).

*Naucoris parvulus*: Poisson 1963, Bull. Inst. Franc. Afr. Noire 25: 1181 (distribution).

*Naucoris parvulus*: La Rivers 1971, Biol. Soc. Nev. Mem. 2: 72 (catalog).

*Afronaucoris parvulus*: Sites 2022, Zool. J. Linn. Soc. 195: 1277.

**Fig 26:**
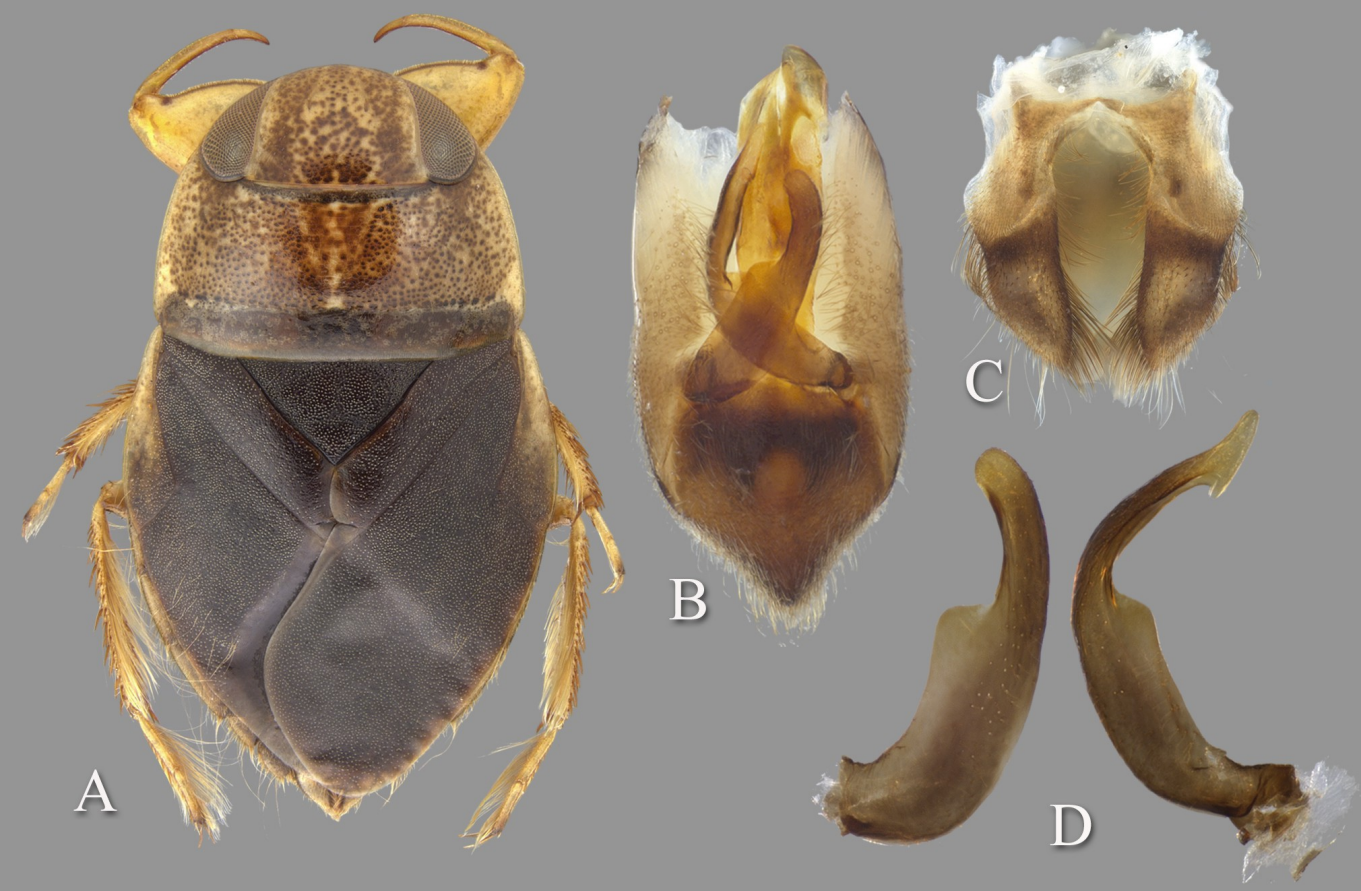
Afronaucoris parvulus. (A) Male habitus, (B) male genital capsule with proctiger and tergum IX removed, (C) male 8^th^ abdominal tergum, (D) dorsal view of left and right parameres.

### Discussion

This is the smallest species of saucer bug in Madagascar. Measurements of 16 randomly chosen specimens are length 5.84–6.80 (mean = 6.29); maximum width 3.52–4.16 (mean = 3.71). It is a common inhabitant of aquatic vegetation in ponds, marshes, swamps, rice paddies, and stream margins. This species is abundant and occurs syntopically with *A*. *madagascariensis*, but in much greater numbers.

### Diagnosis

With the apparent absence of Signoret type specimens, our concept of *A*. *parvulus* is dependent on Montandon-examined specimens housed in MCSN and MNHN (see Uknown Province in Material examined). Although there is overlap in size among individuals of both species, *A*. *parvulus* is somewhat smaller than *A*. *madagascariensis*; however, surface texture of the pronotum will unmistakably distinguish between them. The pronotum of *A*. *parvulus* is smooth and glossy with only negligible indications of dimpled punctation (Fig 26A), whereas that of *A*. *madagascariensis* is obviously dimpled and less reflective because of its matte appearance (Fig 25A). Genitalia (Fig 26B–26D) are similar to those of *A*. *madagascariensis*.

### Published records

Antananarivo [39]; lac Alaotra, Angavokely, Ankarara [34]; Fianarantsoa, Tamatave, Tananarive, Tuléar [28].

### Material examined

**Antananarivo**: Analamanga, 2km W of Manjakandriana, 18.9213S, 47.8335E, 1390 m, 24-XI-2014, leg. Bergsten, Bukontaite, Randriamihaja, Ranarilalatiana, Holmgren, MAD14-80 (5♂, 5♀ NHRS); Vakinankaratra: Manjakatompo Stn. Forestière, Poste, S19.354194, E047.308083, 1796 m, 24-I-2012, lake w grass, leg. T. Ranarilalatiana & J. H. Randriamihaja, MJK12-14 (1♀ UMC); Analamanga: RN2, 5 km W of Manjakandriana, S18.91754, E047.83444, 1370 m, 6-XI-2011, pond with vegetation, leg. J. Bergsten, R. Bukontaite, T. Ranarilalatiana, & J.H. Randriamihaja, MAD11-20 (2♂, 3♀ UMC); Andranofena River at Andranofena Sud village, 18.0844S, 47.1776E, 1430 m, 21-XI-2014, leg. Bergsten, Bukontaite, Randriamihaja, Ranarilalatiana, Holmgren, MAD14-74 (1♂, 1♀ UMC; 4♂, 5♀, 4 nymphs NHRS); Vakinankaratra, Manjakatompo Stn. Forestière, Analafandriana, S19.35806, E047.31401, 1730 m, 3-XI-2011, stream with wet field, leg. J. Bergsten, R. Bukontaite, T. Ranarilalatiana, & J.H. Randriamihaja, MAD11-13 (8♂, 4♀ UMC); Vakinankaratra, Manjakatampa Stn., Forestière, Lac Froid, S19.34295, E47.3390, 1640 m, 3-XI-2011, J. Bergsten, MAD11-15 (1♀ UMC); Vakinankaratra: Manjakatompo Stn. Forestière, 500 m E Lac Froid, S19.34485, E047.33381, 1620 m, 4-XI-2011, pond and inlet stream, leg. J. Bergsten, R. Bukontaite, T. Ranarilalatiana, & J.H. Randriamihaja, MAD11-16 (1♀ UMC); Vakinankaratra: Manjakatompo Stn., Forestière, Analafandriana, 500 m S fish farm by road, S19.36191, E047.31495, 1730 m, 3-XI-2011, grassy pond, leg. J. Bergsten, R. Bukontaite, T. Ranarilalatiana, & J.H. Randriamihaja, MAD11-14 (5♂, 5♀ UMC; 3 nymphs NHRS); Vakinankaratra, Manjakatompo Stn. Forestière, 2 km NE Amparandraindrisa, S19.36067, E047.30098, 1770 m, 5-XI-2011, pond and stream, leg. J. Bergsten, R. Bukontaite, T. Ranarilalatiana, & J.H. Randriamihaja, MAD11-18 (9♂, 6♀ UMC; 1♂, 1♀ NHRS). **Antsiranana**: stagnant canal in Antsaba, -13.6459S, 48.73429E, 77 m elev, 21-XI-2012, leg. Bergsten Bukontaite, Ranarilalatiana, Randriamihaja, MAD12-23 (1♀, 1 nymph NHRS); Sava, Anjanaribe Sud NP, S14.7528, E49.4875, 990 m, 16-XI-2014, swamp, J. Bergsten, R. Bukontaite, J.H. Randriamihaja, T. Ranarilalatiana, S. Holmgren, MAD14-68 (2♂, 2♀, UMC; 4♂, 3♀, 9 nymphs NHRS); Sava, Anjanaribe Sud NP, 14.7528S, 49.4875E, 15-XI-2014, swamp, leg. Bergsten, Bukontaite, Randriamihaja, Ranarilalatiana, Holmgren, MAD14-65 (1♂, 1♀ UMC; 8♂, 7♀, 24 nymphs NHRS); Sava,14.6996S, 49.5374E, 680 m, 1 km N of Befingitra village, 4 km from Anjanaharibe-Sud Park entrance, 17-XI-2014, muddy pond, J. Bergsten, R. Bukontaite, J.H. Randriamihaja, T. Ranarilalatiana, S. Holmgren, MAD14-72 (12♂, 15♀ UMC; 5♂, 7♀, 20 nymphs NHRS); Andranonakoho, 12.92734S, 49.16309E, 154 m, 3-XII-2012, leg. Bergsten, Bukontaite, Ranarilalatiana, Randriamihaja, MAD12-41 (1♀ NHRS); 14°11.038’ S, 50°1.500’ E, elev. 42 m, 10-XI-2014, R.W. Sites, G. Gustafson, K. Miller, flooded rice paddy & overflow, L-1854 (7♂, 4♀ UMC); Manara River at Antirabe Nord, 13°58.522’ S, 49°57.845’ E, elev. 32 m, 10-XI-2014, R.W. Sites, rock pools & vegetation, L-1852 (1♀ UMC); unnamed stream, 14°3.552’S, 50°1.622’E, elev. 65 m, 10-XI-2014, R.W. Sites & K. Miller, rocky stream w/ veg margins, L-1853 (2♂ UMC); marsh by entrance of Montagen d’Ambre National Park, -12.511389S, 49.18315E, 967 m, 5-XII-2012, leg. Ranarilalatiana, Bergsten, Bukontaite, & Randriamihaja, MAD12-47 (1♂, 1♀ UMC). **Fianarantsoa**: Ranomafana National Park, 21°14.259’ S, 47°23.786’ E, elev. 1138 m, 2-XI-2014, R.W. Sites, J. Bergsten, S. Holmgren, shallow muddy, mossy forest pool, L-1832 (7♂, 5♀ UMC); Ranomafana National Park, 21.23958°S, 47.37926°E, elev. 1141 m, 3-XI-2014, R.W. Sites & S. Holmgren, forest pond with sedges and lilypads L-1840 (11♂, 12♀, 9 nymphs UMC); pond at Ranomafana National Park, 21°14’22.5"S, 47°22’38.1"E, 3-XI-2014, K.B. Miller, KBM03111402 (4♂, 6♀ UMC); Ranomafana, Ifanadiana, Sahamalaotra, 1123 m, N-21.23590, E47.39630, small stream, 6-XII-2004, leg. Balke et al, P27MD31 (1♂, 1♀ UMC); Matsiatra, Ambony, Ranomafana NP, 500 m SW Vohiparara bridge, S21.24029, E047.37725, 1150 m, 31-X-2011, large forest pond, leg. J. Bergsten, R. Bukontaite, T. Ranarilalatiana, & J.H. Randriamihaja, MAD11-08 (1♂, 3♀ NHRS); Matsiatra, Ambony: Ranomafana NP, Sahamalaotra 2 km from Vohiparara, S21.23807, E047.39489, 1140 m, 1-XI-2011, forest bog in rainforest, leg. J. Bergsten, R. Bukontaite, T. Ranarilalatiana, & J.H. Randriamihaja, MAD11-12 (1♂, 1♀ UMC; 5♂, 3♀ NHRS); Matsiara Ambony, Ranomafana NP, Sahamalaotra trail, 21.2382S, 47.3947E, 1130 m, 2-XI-2014, forest marsh, leg. J. Bergsten, T. Ranarilalatiana, S. Holmgren, MAD14-07 (12♂, 5♀ NHRS); Matsiara Ambony, Vohiparara village, 21.2397S, 47.3774E, 1110 m, 3-XI-2014, small forest lake with floating margins of vegetation, leg. J. Bergsten, T. Ranarilalatiana, S. Holmgren, MAD14-10 (1♂, 3♀ NHRS); Ambositra, 4.8 km N Ambatofitorahana, 20°46’22.6"S, 47°10’48.7"E, 1708 m, 1-XI-2014, leg. G. Gustafson & K.B. Miller, pond, GTG110114B (1♂, 2♀ UMC); District Ambositra, S of Ambalamakana, 20°27.381’S, 47°13.855’E, elev. 1328 m, 1-XI-2014, R.W. Sites, pond with lily pads and marginal veg, L-1829 (5♂, 7♀ UMC); Atsimo Antsinanana, 27 km before Vondrozo, S22°48’33.8", E047°09’30.1", 569 m, 12-XII-2013, pond w vegetation, leg. Ranarilalatiana & Randriamihaja, MAD13-69 (1♂, 1 nymph NHRS); Atsimo Antsinanana, R.S. Manombo, Parcelle II, Akoandara, S23°05’08.3", E047°45’37.7", 3 m, 17-XII-2013, pools w vegetation, leg. Ranarilalatiana & Randriamihaja, MAD13-84 (1♀ NHRS); Ihorombe, Pic d’Ivohibe Corridor, S22°28’10.0", E046°56’48.3", 843 m, 9-XII-2013, muddy pools in vegetated swamp, leg. Ranarilalatiana & Randriamihaja, MAD13-60 (1♀ NHRS); Isalo Menamaty River, 757 m, N-22°33.001, E45°24.074, 11-V-2006, degraded river w veg., leg. Bergsten et al, P41AM01, BMNH(E)741830 (2♂ UMC); Andringitra, Zomandao R., Belamba, 9-V-2006, N-22°6.225, E46°55.244, 1421 m, vegetation river margins, leg. Bergsten et al, P39EM08, BMNH(E)741918 (1♂ UMC); Amoron’i Mania, 3 km south of Ambalamanakana next to RN7, Ankazomivady forest, 20.7722S, 47.1809E, 1-XI-2014, marsh with vegetation, leg. J. Bergsten, T. Ranarilalatiana, S. Holmgren, MAD14-02 (7♂, 2♀ NHRS). **Mahajanga Province**: Boeny, Ankarafantsika NP, S16.31215, E046.81523, 76 m, 29-XII-2009, leg. J. Bergsten, N. Jönsson, T. Ranarilalatiana, HJ. Randriamihaja, MAD09-02 (1♂ UMC); Boeny, Mahavavy Kinkony RS, S16.06651, E045.77672, 24 m, 5-XII-2009, leg. J. Bergsten, N. Jönsson, T. Ranarilalatiana, HJ. Randriamihaja, MAD09-29 (1♂ UMC); Melaky btw Bekopaka—Antsalova, S18.91556, E044.55546, 47 m, 16-XII-2009, leg. J. Bergsten, N. Jönsson, T. Ranarilalatiana, HJ. Randriamihaja, MAD09-61 (1♂ UMC); Marovoay Western Madagascar Prov.: Majunga River, Ikopa, 1927 & 28 (5♂, 8♀ UMC; 151 SEMC). **Toamasina**: Alaotra Mangoro, Analamazoatra SR, Andasibe village, S18.92742, E048.42013, 850 m, 8-XI-2011, forest pond, leg. J. Bergsten, R. Bukontaite, T. Ranarilalatiana, & J.H. Randriamihaja, MAD11-22 (1♂, 2♀ UMC; 1♀ NHRS); Alaotra, Mangoro, RNs, Mangoro River, 10 km W of Moramanga, 6-XI-2011, 940 m, S18.92438, E048.18273, river & pools, leg. J. Bergsten, R. Bukontaite, T. Ranarilalatiana, & J.H. Randriamihaja, MAD11-21 (1♀ UMC); Alaotra, Mangoro, Analamazoatra SR, bog at S border of reserve, S18.95456, E048.44048, 910 m, 9-XI-2011, river and side pool, leg. J. Bergsten, R. Bukontaite, T. Ranarilalatiana, & J.H. Randriamihaja, MAD11-27 (2♀ UMC); Alaotra, Mangoro, Mantadia NP, 3 km from park entrance, S18.85262, E048.42721, 920 m, 13-XI-2011, open pond with vegetation, leg. J. Bergsten, R. Bukontaite, T. Ranarilalatiana, & J.H. Randriamihaja, MAD11-42 (4♀ UMC); Antsinanana, RN2, Onibe River, 60 km N Toamasina, S17.65545, E049.4737, 20 m, 15-XI-2011, pond w vegetation, leg. J. Bergsten, R. Bukontaite, T. Ranarilalatiana, & J.H. Randriamihaja, MAD11-54 (2♂, 3♀ UMC); Antsinanana, RN2, 2 km N Brickaville, S18.80477, E049.08473, 80 m, 14-XI-2011, swamp, leg. J. Bergsten, R. Bukontaite, T. Ranarilalatiana, & J.H. Randriamihaja, MAD11-46 (1♂, 1♀ UMC); Antsinanana, RN2, 43 km N Brickaville, S18.51714, E049.14264, 80 m, 14-XI-2011, artificial ponds with vegetation, leg. J. Bergsten, R. Bukontaite, T. Ranarilalatiana, & J.H. Randriamihaja, MAD11-48 (2♂, 1♀ UMC); Antsinanana, RN2, river 13 km N Toamasina, S18.01965, E049.40129, 30 m, 14-XI-2011, leg. J. Bergsten, R. Bukontaite, T. Ranarilalatiana, & J.H. Randriamihaja, MAD11-53 (3♀ UMC); Antsinanana, RN2, Ambodivoanio, 41 km N Toamasina, S17.87002, E049.46249, 20 m, 16-XI-2011, pond w vegetation, leg. J. Bergsten, R. Bukontaite, T. Ranarilalatiana, & J.H. Randriamihaja, MAD11-59 (2♂, 2♀ UMC; 1♂, 1♀ NHRS); Antsinanana, RN2, 10 km S of Brickaville, S18.84045, E49.011669, 10 m, 13-XI-2011, leg. Bukontaite, Bergsten, Randriamihaja, Ranarilalatiana, MAD11-45 (5♂, 2♀ UMC; 1♂, 1♀ NHRS). **Toamasina**: Centmetre, 18°55.626’S, 48°25.219’E, elev. 935 m, 15-XI-2014, R.W. Sites, pond with grasses, L-1869 (1♀ UMC); Morimanga Andasibe, Andasibe National Park, Fish Park, shallow standing water, 5-I-2007, N18.93533, E48.41783, 933 m, leg. Isambert et al, P61BI14 (1♂ UMC). **Toliara**: Menabe, W of Beroboka village, S19°58’12.9", E044°36’05.5", 20 m, 19-XI-2013, rice field, leg. Ranarilalatiana & Randriamihaja, MAD13-04 (2♂, 3♀ NHRS); Zombitse, Ambiamena, edge PN Zombitse, 14-V-2006, N-22°51.605 E44°37.035, 533 m, stagnant zebu-visited marshland, muddy and lots of vegetation, leg. Bergsten et al, P42C, BMNH(E)741973 (1♀ UMC); Atsimo Andrefana, 2 km before Angara village, S21°40’55.3", E043°43’27.6", 30 m, 26-XI-2013, muddy pond, leg. Ranarilalatiana & Randriamihaja, MAD13-21 (1♀ NHRS). **Unknown Province**: Madagas. Pipitz 1883 / Naucoris hydroporoides Bergroth det. A. L. Montandon / Museo Civico di Genova (2♂ MCSN); same but with extra label reading Naucoris hydroporoides Bergroth (1♀ MCSN); Museum Paris, Madagascar, P. Camboue 173–94 / N. parvulus Sign, = N. hydroporoides Bergr., det. A. L. Montandon 1897 (1♀ MNHN); Museum Paris, Madagascar, P. Camboue 173–94 / N. parvulus Sign, det. A. L. Montandon 97 (1♀ MNHN); Museum Paris, Madagascar, P. Camboue 173–94 / N. parvulus Signt, Montand. det. 1897, exchange w/ MNHN (1♀, 1 card-mounted adult UMC).

## Key to the species of Naucoridae of Madagascar

This key is unavoidably heavily reliant on male or female genitalia to distinguish among species of *Macrocoris*, *Temnocoris*, and *Tsingala*. Thus, when collecting, it is best to collect series of specimens to ensure the identifiable sex is available for use with the key. We strongly recommend consulting the figures as referenced in the couplets.

1. Front of head folded posteroventrally so labrum is set back from anterior margin of head (Fig 1). Foreleg pretarsus with two claws. Males with well-developed tomentose patch ventrally on pro- and mesotibiae (on females weakly developed).... ... **Laccocorinae**.. 7
1’. Front of head not folded posteroventrally, so labrum arises near anterior margin of head. Foreleg pretarsus with a single claw. Without tomentose patch on pro- and mesotibiae... 2
2. Females with mediosternite VII (subgenital plate) greatly narrowed in posterior half to form tongue-like lobe. Males with parameres flanking aedeagus, not criss-crossing; aedeagus with complex sclerotized vesica. Body usually large (length ≥ 7.5 mm) and dorsoventrally robust. . . ... .... .... .... .... .... .... .... .... . . ... .... .... .... .. ***Macrocoris***.. 3
2’. Females with mediosternite VII (subgenital plate) at most slightly narrower posteriorly with posterior margin rounded. Males with parameres criss-crossing over aedeagus; aedeagus without sclerotized vesica. Body usually small (length ≤ 8.4 mm) and flattened.... .... .... .... .... .... .... .... .... .... .... .... .... .... .... .... . . .. ***Afronaucoris***.. 6
3. Body length ca. 7.5–8.5 mm. Dorsoventrally robust. Hemelytra with mottled coloration, most evident on clavus (Fig 21A). Phallosoma widening to bulbous distal half (Fig 21B).... .... .... .... .... .... .... .... . . ... .... .... .... .... .... .. ***Macrocoris distinctus***
3’. Body length > 8.5 mm. Dorsoventrally somewhat flattened. Hemelytra usually concolorous brown, grey, or black (Figs 22A, 23A and 24A). Phallosoma continuously slender in distal half (Figs. 22D, 23C and 24C).... .... .... .... .... .... .... .... .... .... .... .... .... .. 4
4. Male left paramere mesal margin in ventral view with a deep concavity at the middle and broadly rounded distally (Fig 22F).... .... .... .... .... .. ***Macrocoris namorona* n.sp.**
4’. Male left paramere mesal margin in ventral view mostly straight distal to the base and tapering apically (Fig 22G–22H). . ... .... .... .... .... .... .... .... .... .... .... . 5
5. Male left paramere mesal margin in ventral view with distal third obtusely angled with base, tapering apically (Fig 22G). Length ca. 13.0–14.5 mm... .... .... ***Macrocoris sikorae***
5’. Male left paramere mesal margin in ventral view with distal third right-angled with base, tapering to a narrowly rounded apex (Fig 22H). Length ca. 12–13 mm... .... .... .... .... .... .... .... .... .... .... .... .... .... .... .... .... ... ***Macrocoris rhantoides***
6. Pronotum with punctures that are clearly dimpled and the entire surface has a matte appearance (Fig 25A).... .... .... .... .... .... .... . ***Afronaucoris madagascariensis***
6’. Pronotum smooth and glossy, indications of punctation are at most negligibly dimpled (Fig 26A).... .... .... .... .... .... .... .... .... .... .... .... .. ***Afronaucoris parvulus***
7. Anterior margin of head angled and without fringe of setae (Fig 1A). Labrum acuminate (Fig 2B). Male pseudoparameres poorly developed (Fig 2C).. .... .... .... .... .... .... .... .... .... .... .... .... .... .... .... ... ***Gonioathrix temnocoroides* n.gen, n.sp.**
7’. Anterior margin of head sharply angled and with fringe of setae (Fig 1B) or broadly rounded dorsoventrally (Fig 1C). Labrum rounded distally. Male pseudoparameres well-developed (e.g., Figs 4B and 15B).. .... .... .... .... .... .... .... .... .... .... .... .. 8
8. Anterior margin of head sharply angled and with fringe of setae (Fig 1B).. ***Temnocoris***.. 9
8’. Anterior margin of head broadly rounded dorsoventrally (Fig 1C).. .... .. . .***Tsingala***. .19
9. Hind trochanters with posterior margin angulate (Figs 8B and 11B).. .... . . ... .... ... . . .10
9’. Hind trochanters with posterior margin convex (e.g., Fig 9B).. .... .... .... .... .... .11
10. Right paramere with anterior margin distinctly sinuate and concave in distal half, apex acuminate (Fig 11F). Aedeagus with apex narrowly rounded on right side (Fig 11D). Female subgenital plate with rigid apical lobes and with lateral margins at angle greater than 45° from long axis of body (sometimes nearly horizontal) (Fig 11G). . . ... .... .... .... .... .... .... .... .... .... .... .... .... .... .... .... .... . ***Temnocoris poissoni* n.sp.**
10’. Right paramere with anterior margin straight to shallowly convex, apex tapered to narrowly rounded tip (Fig 8F). Aedeagus with apex reflexed to right and narrowly rounded (Fig 8D). Female subgenital plate with flap-like apical lobes and with lateral margins convergent at ca. 45° angles (8G).. .... .... .... .... .... .... .... .. ***Temnocoris leachi* n.sp.**
11. Aedeagus with apex truncate (Figs 7C, 12C and 13A).. .... .... .... .... .... .... .... .12
11’. Aedeagus with apex either narrowly rounded, bluntly or sharply hooked, or gradually tapering to a point (e.g., Figs 4C, 5C, 9D and 11D).. .... .... .... .... .... .... .... .. .14
12. Left paramere with rounded lobe on right side in distal third (Fig 12D). Posteromesal corners of pseudoparameres produced posteriorly (Fig 12B).. .... .. ***Temnocoris scarletti***
12’. Left paramere without rounded lobe on right side in distal third (Fig 7D). Posteromesal corners of pseudoparameres narrowly right-angled (Fig 7B) (unknown in *T*. *starmuhlneri*).... .... .... .... .... .... .... .... .... .... .... .... .... .... .... .... .... .... .13
13. Left and right parameres with lateral margins shallowly convex (Fig 7D and 7E). Phallosoma basal to preapical angle widening basally (Fig 7C).... .... .... . ***Temnocoris hungerfordi***
13’. Left and right parameres with lateral margins shallowly concave (Fig 13B and 13C). Phallosoma basal to preapical angle with margins parallel (Fig 13A).... ... ***Temnocoris starmuhlneri***
14. Aedeagus with distal third gradually narrowing to a point (Figs 5C and 10C).... .... .... . 15
14’. Aedeagus with apex either narrowly rounded, or bluntly or sharply hooked (Figs 4C, 6B, 9D and 14C).. .... .... .... .... .... .... .... .... .... .... .... ... . .. . ... .... .... 16
15. Aedeagus with distal third gradually narrowing to a point in a sinuate fashion (Fig 5C). Pseudoparameres with posterior margins straight (Fig 5B)... . . .***Temnocoris andringitrae***
15’. Aedeagus with distal third gradually narrowing to a point while consistently curving to the right (Fig 10C). Pseudoparameres with posterior margins convex (Fig 10B).... .... .... .... .... .... .... .... .... .... .... .... .... .... .... .... .. . .***Temnocoris perplexus***
16. Right paramere slender, almost boomerang-like in shape (Figs 4E and 14E).... .... .... . 17
16’. Right paramere not slender, similar in shape to left paramere (Figs 6C, 9E and 9F)... . ... .. 18
17. Aedeagus with apical notch and pronounced subapical lobe on right side (Fig 4C). Pseudoparameres with posterolateral corners right-angled (Fig 4B). Female subgenital plate lateral margins straight or shallowly concave, apex truncate (Fig 4F).. .... .... .... .... .... .... .... .... .... .... .... .... .... .... .... .... .... .. ***Temnocoris ambositrae***
17’. Aedeagus with apical blunt production on right side (Fig 14C). Pseudoparameres with posterolateral corners produced laterally (Fig 14B). Female subgenital plate lateral margins distinctly concave, apex with median notch (Fig 14F).. ...***Temnocoris translucidus***
18. Aedeagus apex sharply hooked and with deep subapical notch and pronounced bulge on right side (Fig 9D). Left paramere angled at midlength on lateral margin (Fig 9E); right paramere shallowly convex on distal margin (Fig 9F)... . . .***Temnocoris montandoni* n.sp.**
18’. Aedeagus apex sharply hooked and with shallow subapical concavity and subtle bulge on right side (Fig 6B). Left paramere rounded on lateral margin, right paramere straight or shallowly concave on distal margin (Fig 6C).... .... .... .... .... .***Temnocoris dubius***
19. Eyes noticeably convergent anteriorly. Body length ≤8.5 mm.... .. ***Tsingala nossibeanus***
19’. Eyes parallel or only slightly convergent anteriorly. Body length variable... .... .. . .20
20. Female subgenital plate posterior margin with three lobes (Fig 20B).... .... .... .... .... .... .... .... .... .... .... .... .... .... .... .... .... .... ***Tsingala trilobata* n.sp.**
20’. Female subgenital plate posterior margin straight, angled, or rounded (e.g., Figs 17D, 18B and 19B).. .... .... .... .... .... .... .... .... .... .... .... .... .... .... .... .... .21
21. Female subgenital plate posterior margin wide and truncate (Fig 19B). Hemelytra mostly concolorous dark-brown or black (all known specimens are submacropterous) (Fig 19A).... .... .... .... .... .... .... .... .... .... .... .... .... .... . ***Tsingala spatulata* n.sp.**
21’. Female subgenital plate posterior margin angled or rounded (e.g., Figs 17D and 18B). Hemelytra macropterous or submacropterous, concolorous, speckled, or mottled... .... 22
22. Female subgenital plate posterior margin rounded; posterolateral corners of mediosternite VI rounded (Figs 16C and 17B).... .... .... .... .... .... .... .... .... .... .... .... 23
22’. Female subgenital plate posterior margin angled; posterolateral corners of mediosternite VI narrowly rounded or angulate (Figs 15F and 18B).... .... .... .... .... .... .... .... .. 24
23. Left paramere of male nearly half as wide as long (Fig 16F). Ground color orangish-brown, especially evident ventrally (Fig 16A and 16C).... .... .... ... . . .***Tsingala humeralis***
23’. Left paramere of male nearly as wide as long (Fig 17E). Ground color mostly dark-brown (Fig 17A and 17D).. .... .... .... .... .... .... .... .... .... .... ***Tsingala latiforma* n.sp.**
24. Female subgenital plate posterior margin truncate medially, angled at ca. 45°, then straight or shallowly concave to base (Fig 15F).... .... .... .... .... .. ***Tsingala angulata* n.sp.**
24’. Female subgenital plate posterior margin truncate medially, then shallowly convex to base (Fig 18B).. .... .... .... .... .... .... .... .... .... .... .... .***Tsingala naucoroides***
